# C6orf223 promotes colorectal cancer growth and metastasis by facilitating PRMT5-MEP50 multiprotein complex assembling

**DOI:** 10.1172/JCI186052

**Published:** 2025-10-15

**Authors:** Yufeng Qiao, Zhenzhen Wu, Peng Wang, Yiliang Jin, Furong Bai, Fei Zhang, Yunhe An, Meiying Xue, Han Feng, Yong Zhang, Yaxin Hou, Junfeng Du, Huiyun Cai, Guizhi Shi, Bing Zhou, Pu Gao, Jizhong Lou, Peng Zhang, Kelong Fan, Jinbo Liu, Pengcheng Bu

**Affiliations:** 1 Key Laboratory of Epigenetic Regulation and Intervention, Institute of Biophysics, Chinese Academy of Sciences, Beijing, China.; 2 College of Life Sciences, University of Chinese Academy of Sciences, Beijing, China.; 3 State Key Laboratory of Biomacromolecules, Institute of Biophysics, Chinese Academy of Sciences, Beijing, China.; 4 Institute of Analysis and Testing, Beijing Academy of Science and Technology (Beijing Center for Physical and Chemical Analysis), Beijing, China.; 5 State Key Laboratory of Stem Cell and Reproductive Biology, Institute of Zoology, Chinese Academy of Sciences, Beijing, China.; 6 Department of General Surgery, the Seventh Medical Center, Chinese PLA General Hospital, Beijing, China.; 7 University of Chinese Academy of Sciences, Beijing, China.; 8 Beijing Key Laboratory for Genetics of Birth Defects, Beijing Pediatric Research Institute, MOE Key Laboratory of Major Diseases in Children, Rare Disease Center, Beijing Children’s Hospital, Capital Medical University, National Center for Children’s Health, Beijing, China.; 9 Department of Colorectal Surgery of the First Affiliated Hospital of Zhengzhou University, Zhengzhou, China.

**Keywords:** Cell biology, Oncology, Colorectal cancer

## Abstract

Protein arginine methyltransferase 5 (PRMT5) complexes with methylosome protein 50 (MEP50) play crucial roles in tumor progress. However, the regulatory mechanism of governing the PRMT5-MEP50 hetero-octameric complex remains unclear. Here, we demonstrate that C6orf223, to our knowledge an uncharacterized protein, facilitates PRMT5-MEP50 multiprotein complex assembling, thereby promoting colorectal cancer (CRC) growth and metastasis. C6orf223 forms dimers through disulfide bonds, with its N-terminal arginine-enriched region binding to the C-terminal negatively charged groove of PRMT5, thus stabilizing PRMT5-MEP50 multiprotein and enhancing PRMT5 methyltransferase activity. Consequently, PRMT5-mediated H4R3me2s substantially decreases the expression of the tumor suppressor GATA5, leading to the upregulation of multiple oncogenic target genes including WWTR1, FGFR1, and CLU. Targeting C6orf223 using siRNAs encapsulated in ferritin protein shells effectively suppresses CRC tumor growth and metastasis. Collectively, our findings characterize the role of C6orf223 in facilitating PRMT5-MEP50 hetero-octameric complex assembling and suggest that C6orf223 could serve as a potential therapeutic target for CRC.

## Abstract 2

C6orf223 significantly promote CRC growth and metastasis through elevating PRMT5 activity which decreases the expression of the tumor suppressor GATA5.

## Introduction

Colorectal cancer (CRC) is the third most diagnosed and second most fatal cancer globally (1). Although surgical resection has a potentially curative impact for early-stage CRC, the majority of CRC is diagnosed in the middle or late stages, along with liver metastasis, which is the key cause of the high fatality rate (2, 3). The fact that current CRC treatments do not explicitly target liver metastases places an emphasis on understanding the mechanism and targets of CRC metastasis.

Dysregulation of histone modification such as lysine and arginine methylation is a crucial characteristic of CRC (4). Protein arginine methyltransferase 5 (PRMT5) is the major symmetric arginine dimethyltransferase in mammalian cells, catalyzing diverse substrates including histone H4 (5), thereby regulating the expression of genes involved in mRNA splicing, translation, and growth factor–associated signaling and modulating a variety of cellular processes, such as DNA damage response, tumor immunity, and cell cycle progression (6–14). The potent role of PRMT5 makes it a promising therapeutic target for cancer treatment, and efforts are being made to understand its regulation mechanisms (15, 16). Genetic alterations are rare in the *PRMT5* gene (17). Thus, studies have focused on the regulation of PRMT5 expression, such as transcriptional activation and protein stability, or partners PRMT5 interacts with, such as methylosome protein 50 (MEP50) (18–24). Four PRMT5 monomers form a hetero-octameric complex with 4 other MEP50 monomers (25, 26). The hetero-octameric complex is essential for the arginine dimethyltransferase activity of PRMT5 and seems to be dynamically assembled among the PRMT5 monomer, tetramer, and hetero-octamer (26, 27). However, the regulatory mechanism of the hetero-octameric complex remains to be elucidated.

PRMT5-mediated symmetric demethylation of H4R3 (H4R3me2s) transcriptionally represses a series of tumor suppressors (28–30). GATA binding protein 5 (GATA5) is a transcription factor, originally identified as essential for cardiovascular and genitourinary development (31, 32). A growing body of evidence has shown that GATA5 plays an important role in cancer progression as well. The GATA5 level is clearly low in CRC and renal cell cancer tissues compared with normal tissues, making it a potential biomarker for the detection and prognosis of these cancer types (33–35). In addition, low expression of GATA5 has been reported to be involved in the progression of breast cancer and prostate cancer (36, 37). In contrast to the important function of GATA5, the mechanisms that GATA5 is involved in biological processes remain to be investigated. The studies on the regulation of GATA5 expression have mostly concentrated on the methylation of its promoter, and the direct targets of GATA5 remain largely unclear.

In this study, we identified a protein — to our knowledge previously uncharacterized — encoded by the *C6orf223* gene, which binds to PRMT5 to facilitate the PRMT5-MEP50 hetero-octameric complex assembly, thereby increasing PRMT5 activity and the enrichment of H4R3me2s in a series of genes. In contrast to the higher expression level of C6orf223 in liver metastases relative to primary CRC, PRMT5 has comparable expression levels in primary CRC and liver metastases, whereas PRMT5 activity is enhanced in liver metastases. An orthotopic mouse model demonstrated that C6orf223 promoted CRC growth and metastasis through elevating PRMT5 activity. We identified PRMT5-mediated increased enrichment level of H4R3me2s on GATA5 promoter and enhancer regions and 3 GATA5 targets (WWTR1, CLU, and FGFR1), which responded to the accelerated effect of the C6orf223/PRMT5 axis on CRC growth and metastasis. Using siRNAs encapsulated in a ferritin protein shell to target C6orf223 effectively inhibited CRC tumor growth and metastasis.

## Results

### PRMT5 expression level is not related to CRC liver metastasis.

**Figure 1:**
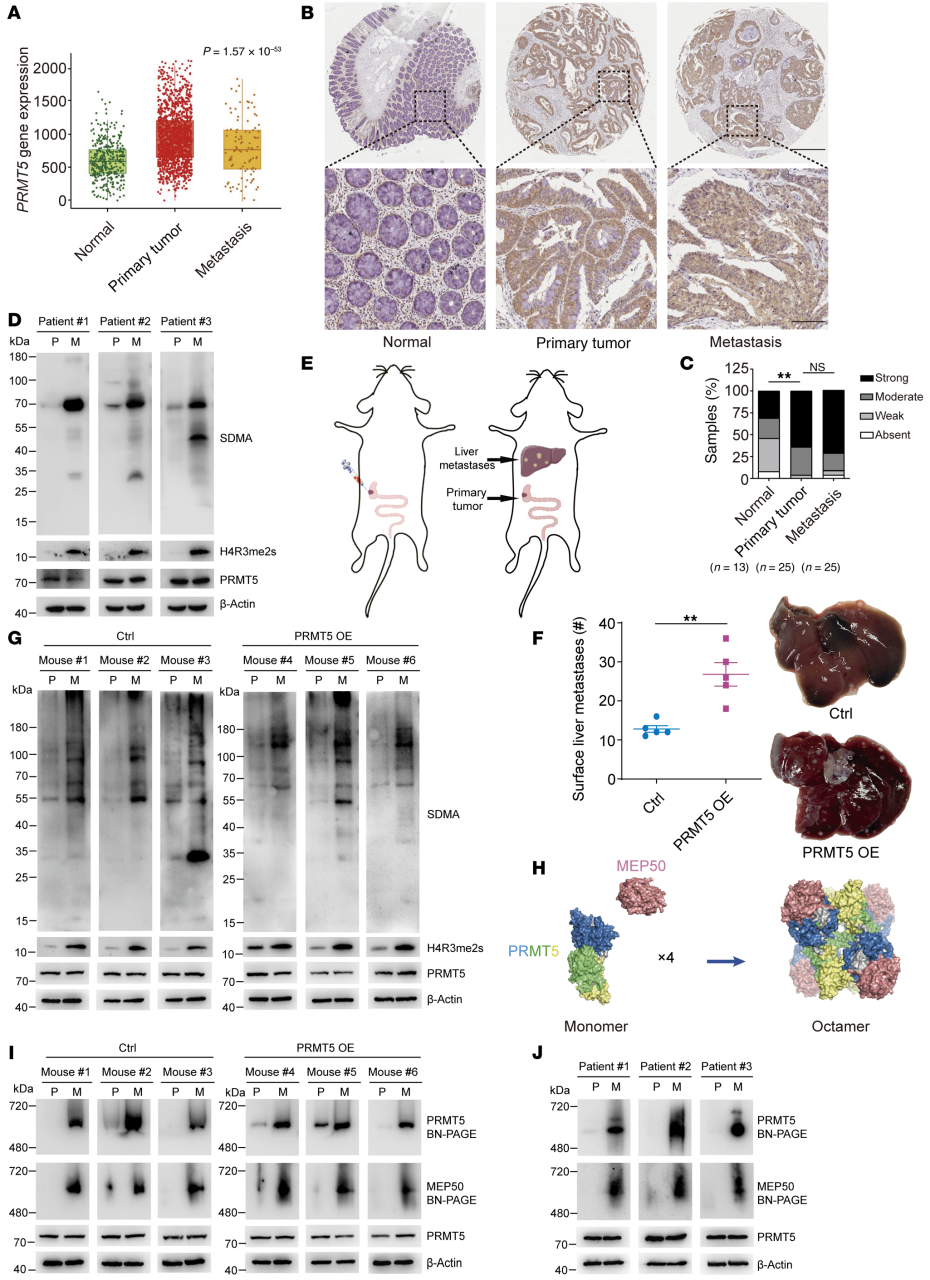
Increased PRMT5-MEP50 hetero-octamer level and PRMT5 methyltransferase activity in CRC liver metastases. (**A**) Comparison of the levels of *PRMT*5 between pericarcinomatous tissue (normal, *n* = 377), primary CRC (tumor, *n* = 1,450) and liver metastatic samples (metastasis, *n* = 99) using TNMplot. *P* value was calculated by Kruskal-Wallis test. (**B** and **C**) Representative IHC images (**B**) and quantification (**C**) of PRMT5 levels on a tissue microarray containing 13 paired pericarcinomatous tissue samples, primary CRC and liver metastatic samples, and 12 paired primary colon tumor and liver metastatic samples. Upper scale bar: 500 μm; lower scale bar: 100 μm. *P* value was calculated by χ^2^ test (NS, **P < 0.01). (**D**) Western blot showing SDMA and PRMT5 levels in paired primary CRC (P) and liver metastatic samples (M). (**E**) Schematic of the CRC orthotopic mouse model. (**F**) Representative bright-field images (right) and quantification (left) of the metastases on the liver surface in NCG mice with cecum injection of HT29 cells stably expressing PRMT5 or not. Data are shown as the mean ± SEM of 5 mice per group. *P* value was calculated by 2-tailed paired Student’s *t* test (**P < 0.01). (**G**) Western blot showing SDMA, H4R3me2s and PRMT5 levels in paired primary tumor (P) and liver metastasis (M) of NCG mice with cecum injection of HT29 cells stably expressing PRMT5 or not. (**H**) Schematic of the process of PRMT5 monomer and MEP50 monomer forming PRMT5-MEP50 hetero-octamer. MEP50 monomers are colored pink. N-term, middle; C-term of PRMT5 are colored blue, yellow, and green, respectively. (**I**) Blue native PAGE showing the levels of PRMT5 hetero-octamer in paired primary tumor (P) and liver metastasis (M) of NCG mice with cecum injection of HT29 cells stably expressing PRMT5 or not. (**J**) Blue native PAGE showing the levels of PRMT5 hetero-octamer in paired primary tumor (P) and liver metastases (M) of patients with CRC.

Cancer metastasis often arises due to elevated expression of certain oncogenic genes (38). To investigate whether PRMT5 potentially associates with CRC metastasis, we analyzed its expression in 377 normal colon, 1,450 primary CRC, and 99 CRC liver metastatic samples on TNMplot (39). We found that *PRMT5* had similar expression levels in primary CRC and liver metastases, although its expression was significantly elevated in CRC samples compared with normal colon tissues (Figure 1A). We further examined PRMT5 expression on a tissue array that contained 13 normal colon tissues and 25 pair-matched primary CRC and liver metastatic samples. Consistently, the PRMT5 expression level was elevated in CRC tissues, while comparable in primary and liver metastatic sites (Figure 1, B and C). Moreover, we compared PRMT5 protein levels between pair-matched primary tumors and liver metastatic samples of patients with CRC, and found that primary tumors and liver metastases still had comparable PRMT5 levels (Figure 1D and Supplemental Figure 1A; supplemental material available online with this article; https://doi.org/10.1172/JCI186052DS1). These data indicate that PRMT5 expression level is not correlated with CRC liver metastasis.

### PRMT5 promotes CRC liver metastasis.

To directly investigate the influence of PRMT5 on CRC metastasis, we used an orthotopic tumor-bearing mouse model (40, 41). We ectopically expressed PRMT5 in CRC cell line HT29 and injected the cells into the terminal cecum walls of NOD/ShiLtJGpt-*Prkdc^em26Cd52^Il2rg^em26Cd22^*/Gpt (NCG) mice (Supplemental Figure 1C and Figure 1E). About 4 weeks later when the mice appeared moribund, all mice were euthanized and dissected. The weight of the primary tumor, the degree of liver metastasis, and PRMT5 expression in the tumor tissues were then evaluated. Notably, we found that PRMT5 substantially promoted primary tumor growth and liver metastasis (Figure 1F and Supplemental Figure 1, D and E), and its expression levels were comparable in primary tumors and liver metastases in the mice (Figure 1G and Supplemental Figure 1F). To clarify the direct roles of PRMT5 in liver metastasis in CRC, we used a spleen injection mouse model by injecting control and PRMT5-overexpressed HT29 cells into the spleen. The model also demonstrated that PRMT5 overexpression enhanced liver metastasis (Supplemental Figure 1, G and H). Thus, we conclude that PRMT5 promotes CRC liver metastasis.

### CRC liver metastases have elevated PRMT5 activity and PRMT5-MEP50 multiprotein complex level.

As a type II arginine methyltransferase, PRMT5 catalyzes symmetric dimethylation of arginine (SDMA) in diverse substrates (42). We therefore speculated that PRMT5 has higher methyltransferase activity in liver metastases, which contributes to CRC metastasis. We then evaluated the methyltransferase activity of PRMT5 by using an antibody that specifically recognizes endogenous proteins in which arginine is symmetrically dimethylated (anti-SDMA) (7, 43). SDMA levels were indeed elevated in the liver metastasis compared with the primary tumors collected from CRC orthotopic tumor-bearing mice (Figure 1G). Consistently, CRC liver metastases had more SDMA relative to the pair-matched primary CRC tissues collected from patients with CRC (Figure 1D, and Supplemental Figure 1, A and B). Thus, PRMT5 had much higher arginine methyltransferase activity in CRC liver metastases. In addition to PRMT5, which produces SDMA, PRMT9 also catalyzes SDMA. We next examined the RNA levels of *PRMT9* in normal tissues, primary CRC, and metastases using The Cancer Genome Atlas (TCGA) database (Supplemental Figure 1I). Moreover, we measured the protein levels of PRMT9 in the pair-matched primary CRC tissues and liver metastases collected from patients with CRC (Supplemental Figure 1, J and K). The results showed that the *PRMT9* RNA level slightly decreased in liver metastases compared with primary CRC (Supplemental Figure 1I). However, Western blot showed that the PRMT9 protein level was similar in primary CRC and liver metastases (Supplemental Figure 1, J and K).

Biochemical and structural evidence shows that PRMT5 and MEP50 form a hetero-octamer with 4 monomers of each, and the hetero-octameric complex is essential for PRMT5 activity (Figure 1H) (6, 26). Since PRMT5 activity is elevated in CRC liver metastases, we speculated that the level of PRMT5-MEP50 hetero-octamer might be higher in CRC liver metastases as well. Therefore, we examined the levels of PRMT5-MEP50 multiprotein complex in pair-matched primary tumors and liver metastases collected from orthotopic tumor-bearing mice and patients with CRC. We performed blue native PAGE using the cell lysates, which enables high-resolution separation of native multimers without disrupting the interaction between proteins (44). After measuring PRMT5 and MEP50 by Western blot, we found that the levels of PRMT5-MEP50 multiprotein complex in liver metastases were substantially increased both in the orthotopic mouse models and CRC clinical samples (Figure 1, I and J). Taken together, these results show that CRC liver metastases have higher PRMT5 activity than primary tumors, likely due to elevated PRMT5-MEP50 multiprotein complex levels.

### C6orf223 facilitates PRMT5-MEP50 multiprotein complex assembling by interacting with PRMT5.

**Figure 2:**
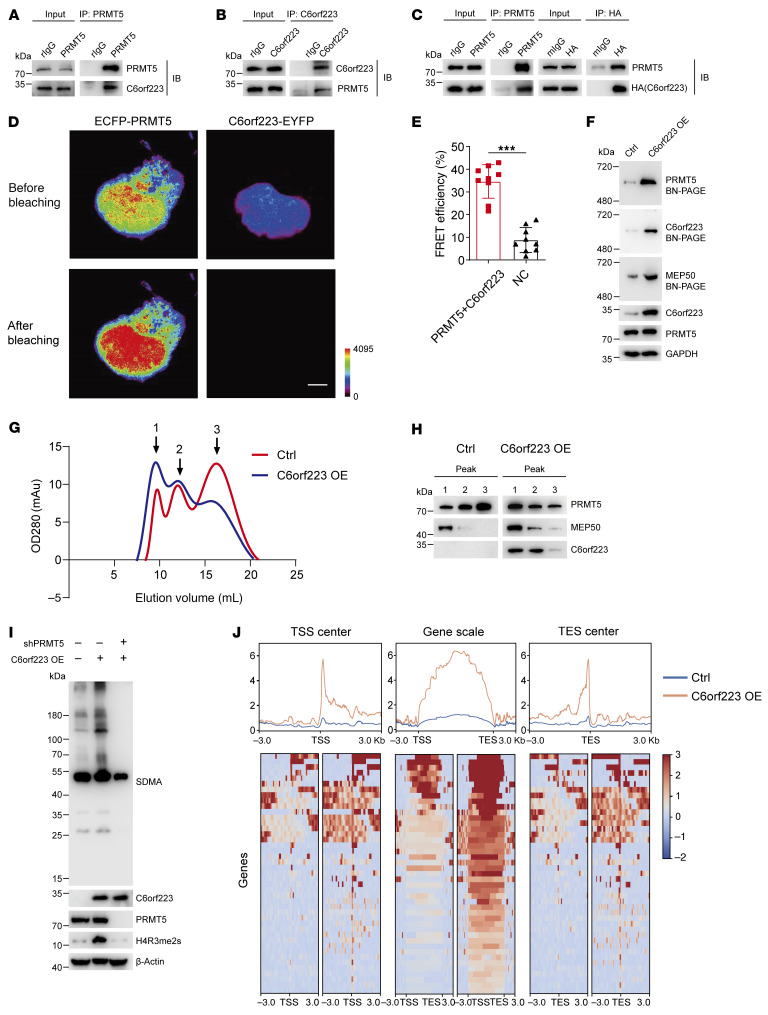
C6orf223 facilitates PRMT5-MEP50 hetero-octamer assembling. (**A**) Co-IP with anti-PRMT5 antibody in HCT116 cell lysates, followed by Western blot for PRMT5 and C6orf223. rIgG was the control. (**B**) Co-IP with anti-C6orf223 antibody in HCT116 cell lysates, followed by Western blot for PRMT5 and C6orf223. rIgG was the control. (**C**) Co-IP with anti-PRMT5 or anti-HA antibody in cell lysates of HEK293T cells transfected with PRMT5 and C6orf223 with HA-tag, followed by Western blot for PRMT5 and HA. (**D**) FRET microscopy analysis of HEK293T cells with ectopic ECFP-PRMT5 and C6orf223-EYFP expression. FRET images are represented in pseudo-color mode. Images represent the fluorescence intensity before (upper) or after (lower) acceptor photobleaching. Scale bar: 5 μm. (**E**) FRET efficiency of **D**. Data are shown as the mean ± SD of 9 repeats per group. *P* value was calculated by 2-tailed paired Student’s *t* test (***P < 0.001). (**F**) Blue native PAGE showing the levels of PRMT5-MEP50 hetero-octamer in HT29 cells with empty vector (Ctrl) or ectopic C6orf223 expression (C6orf223 OE). The membrane was blotted for PRMT5, C6orf223, and MEP50. (**G**) Size exclusion chromatography elution profiles for PRMT5-MEP50 hetero-octamer in 293-F cells with empty vector (Ctrl) or ectopic C6orf223 expression (C6orf223 OE). Peaks 1, 2, and 3 represent octamer, tetramer, and monomer, respectively. OD280 denotes absorbance at 280 nm. (**H**) SDS-PAGE of fractions collected from peaks 1, 2, and 3 of the 2 groups. (**I**) Western blot showing the levels of SDMA and H4R3me2s in HT29 cells with empty vector, ectopic C6orf223 expression, and ectopic C6orf223 expression together with PRMT5 knockdown. (**J**) Genic distribution of H4R3me2s in HT29 cells with empty vector (Ctrl) or ectopic C6orf223 expression (C6orf223 OE). Genic regions with upregulated H4R3me2s under ectopic C6orf223 expression are depicted in transcription start site–centered, gene-scaled, and transcription enhancer site–centered regions in the heatmap (bottom panel) and summarized profile (top panel).

To understand the mechanism that facilitates PRMT5-MEP50 multiprotein complex assembling, we first performed co-IP–mass spectrometry to identify proteins interacting with PRMT5 in CRC cells (Supplemental Figure 2A). We then analyzed the upregulated genes in liver metastasis compared with primary CRC tissues in the NCBI’s Gene Expression Omnibus (GEO) database. Subsequently, we identified genes upregulated in liver metastases and interacting with PRMT5 through integrated analysis of GEO database screening and co-IP–mass spectrometry data. Since the modification of H4R3 to H4R3me2s occurs in the nucleus, we further screened genes enriched in the nucleus. Finally, C6orf223 emerged as the top candidate protein (Supplemental Figure 2, B–D). In addition, we also found that the expression of C6orf223 was aberrantly upregulated in various types of CRC (Supplemental Figure 2E). C6orf223 is annotated as the lincRNA *LINC03040* in the NCBI’s Gene database. We further analyzed the gene structure of *LINC03040* and identified an open reading frame (ORF) using the NCBI’s ORF Finder tool. The ORF presents an ATG start codon and a TAA end codon. The putative small peptide consists of 242 amino acids, which is consistent with the amino acid sequence of C6orf223, indicating that the small peptide encoded by *LINC03040* is C6orf223 (Supplemental Figure 2F). Co-IP with C6orf223 or PRMT5 confirmed that C6orf223 and PRMT5 indeed interacted in HCT116 CRC cells (Figure 2, A and B), which was further validated by a pulldown assay in HEK293T cells with ectopically expressed PRMT5 and HA-tagged C6orf223 (Figure 2C). We further verified the interaction of C6orf223 and PRMT5 using fluorescence resonance energy transfer (FRET), an approach for measuring direct interaction in live cells (Figure 2, D and E). To examine whether C6orf223 interacts with MEP50, we expressed PRMT5, His-tagged MEP50, and HA-tagged C6orf223 in HEK293T cells or His-tagged MEP50 with HA-tagged C6orf223 and without PRMT5 in HEK293T cells. Pulldown with anti-HA or anti-His antibodies showed that C6orf223 complexed with MEP50 when PRMT5 was present, while C6orf223 or MEP50 could not pull down each other when PRMT5 was absent (Supplemental Figure 3, A and B), indicating the indirect interaction between C6orf223 and PRMT5. Additionally, we performed co-IP–mass spectrometry to determine whether any other PRMTs interacted with C6orf223. The results showed that PRMT5 was the only member of the PRMT family that interacted with C6orf223 (Supplemental Figure 3C).

We then sought to find out whether the interaction between C6orf223 and PRMT5 influences PRMT5-MEP50 multiprotein complex assembling. We ectopically expressed C6orf223 in HT29 cells, a CRC cell line with a relatively low expression level of C6orf223 (Figure 2F and Supplemental Figure 3D), and knocked down C6orf223 in HCT116 cells with a relatively high expression level of C6orf223 (Supplemental Figure 3E). Blue native PAGE showed that PRMT5-MEP50 multiprotein complex was increased when C6orf223 was ectopically expressed and reduced when C6orf223 was knocked down (Figure 2F and Supplemental Figure 3E). To further validate the observations, we performed size exclusion chromatography to measure the influence of C6orf223 on PRMT5-MEP50 multiprotein complexes. We coexpressed PRMT5, MEP50 with C6orf223, or an empty expression vector in 293-F cells. The complexes were purified and then applied to size exclusion chromatography. The OD280 of the effluent was monitored to measure protein levels. Three peaks were observed and likely represented PRMT5-MEP50 hetero-octamer, PRMT5/C6orf223 tetramer, and PRMT5 monomer (Figure 2G). Moreover, ectopic expression of C6orf223 increased the PRMT5-MEP50 hetero-octamer and PRMT5/C6orf223 tetramer, while decreasing the PRMT5 monomer fraction (Figure 2H). Taken together, the results show that C6orf223 interacts with PRMT5 and facilitates PRMT5-MEP50 multiprotein complex assembling.

### C6orf223 increases PRMT5 methyltransferase activity.

Consistent with the observation that C6orf223 increases PRMT5-MEP50 multiprotein complex, we found that ectopic expression of C6orf223 upregulated SDMA and H4R3me2s levels in HT29 CRC cells, and the effects were abrogated when PRMT5 was knocked down (Figure 2I). Conversely, C6orf223 knockdown substantially reduced SDMA and H4R3me2s levels (Supplemental Figure 3E). The results suggest that C6orf223 enhanced PRMT5 methyltransferase activity, particularly histone H4 methylation. To globally explore the genes associated with C6orf223-mediated enrichment changes of H4R3me2s, we overexpressed C6orf223 in HT29 cells and then performed ChIP-Seq for H4R3me2s. We found that C6orf223 indeed increased enrichment of H4R3me2s associated with dozens of genes. Moreover, most of the increased enrichment of H4R3me2s occurred near transcription start sites, transcription enhancer sites, and in the gene body region (Figure 2J).

### C6orf223 is dimerized and facilitates PRMT5-MEP50 multiprotein complex assembling through interaction between its N-terminal arginine-enriched region and PRMT5 C-terminal negatively charged groove.

**Figure 3:**
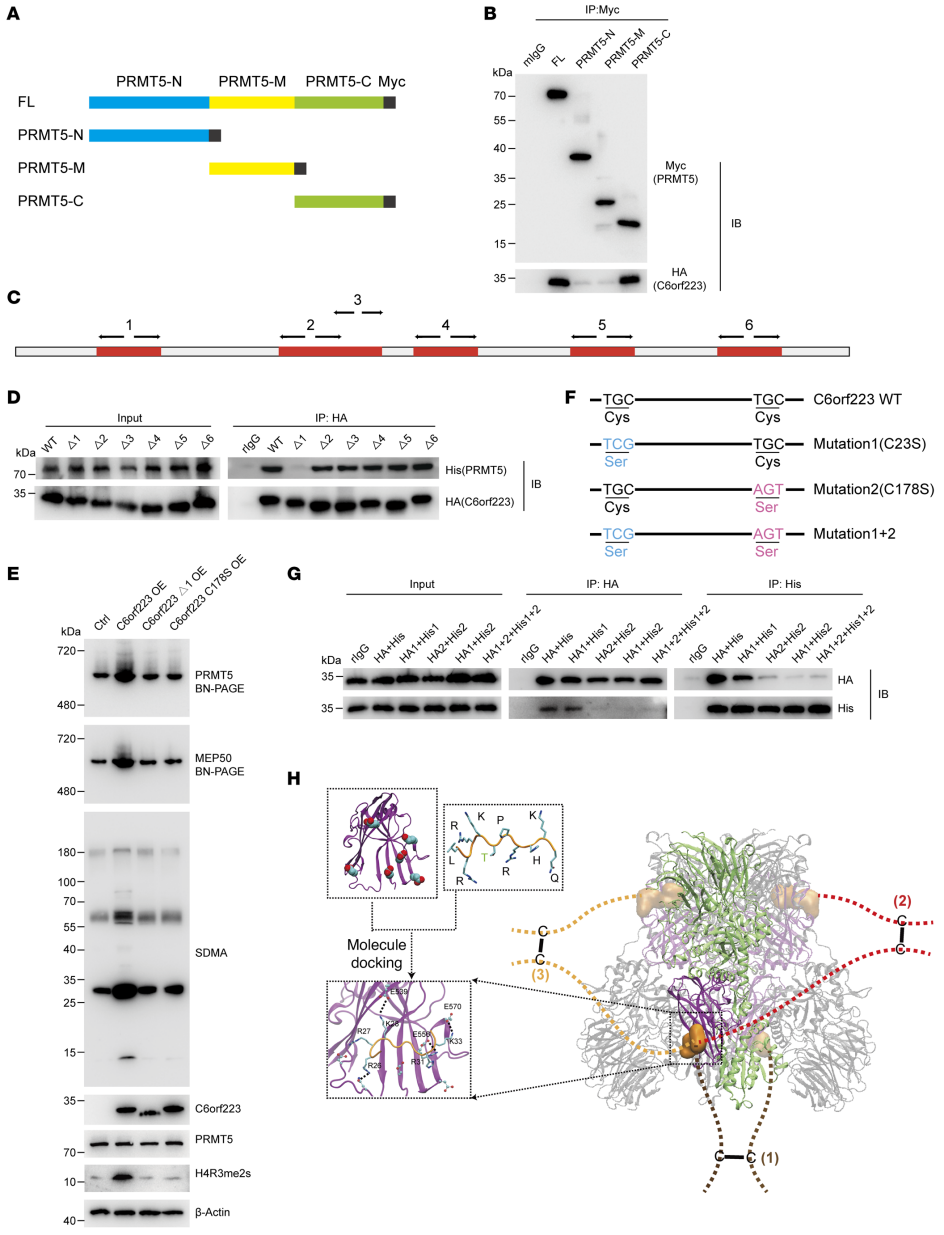
Molecular mechanism of C6orf223-mediated PRMT5-MEP50 hetero-octamer assembling. (**A**) Schematic diagram of the full-length (FL) and different truncates of PRMT5 tagged with Myc-tag. (**B**) IP with anti-Myc antibody in HEK293T cells transfected with HA-tagged C6orf223 together with Myc-tagged FL or different truncates of PRMT5, followed by Western blot for HA and Myc. (**C**) Schematic of 6 arginine-enriched domains of C6orf223 (red). (**D**) IP with anti-HA antibody in HEK293T cells transfected with His-tagged PRMT5 together with WT or HA-tagged C6orf223 truncates (Δ1, Δ2, Δ3, Δ4, Δ5, Δ6), followed by Western blot for HA and Myc. (**E**) Blue native PAGE showing the levels of PRMT5-MEP50 hetero-octamer and Western blot showing the levels of SDMA in HT29 cells transfected with C6orf223 or different C6orf223 mutants. (**F**) Schematic of C6orf223 mutants (cysteine to serine) tagged with HA-tag or His-tag. (**G**) IP with anti-HA or anti-His antibody in HEK293T cells transfected with different C6orf223-HA or C6orf223-His mutants, followed by Western blot for HA and His. (**H)** Schematic of C6orf223 facilitating PRMT5-MEP50 hetero-octamer assembling. Dashed lines in brown, red, and yellow are 3 possibilities that C6orf223 dimer interacts with PRMT5. Highlights in orange denote the positively charged amino acid enrichment domains of C6orf223. Highlights in violet denote the negatively charged domains in the C-terminal of PRMT5. Predicted molecule docking between C6orf223 and PRMT5 is shown on the left.

To investigate how C6orf223 facilitates PRMT5-MEP50 multiprotein complex assembling, we first ascertained the interacting region in PRMT5. PRMT5 was truncated into 3 distinct domains, namely the PRMT5-N (aa 37–290), PRMT5-M (aa 294–464 containing the enzyme active site), and PRMT5-C (aa 467–635) (Figure 3A). HA-tagged C6orf223 was then expressed in HEK293T cells together with 3 different Myc-tagged truncated PRMT5. Co-IP with anti-Myc antibody showed that only PRMT5-C retained the interaction with C6orf223 (Figure 3B). PRMT5-C has a negatively charged amino acid residue–rich groove exposed on the surface, while C6orf223 has a number of positively charged amino acid residues. The arginine content was as high as 16.9% (Supplemental Figure 3F). Therefore, we speculated that the interaction between C6orf223 and PRMT5 may depend on electrostatic attraction. We then focused on the 6 arginine-rich regions in C6orf223 (around 12 aa each region) (Figure 3C and Supplemental Figure 3F). We deleted each region separately and coexpressed HA-tagged C6orf223 mutants with His-tagged PRMT5 in HEK293T cells. Co-IP showed that only the mutant lacking the N-terminal arginine-rich region failed to interact with PRMT5 (Figure 3D). In line with this observation, C6orf223 failed to increase PRMT5-MEP50 multiprotein complex and SDMA level when the N-terminal arginine-enriched region was deleted (Figure 3E). Taken together, these results suggest that the N-terminal arginine-enriched region of C6orf223 interacts with the PRMT5 C-terminal negatively charged groove.

Additionally, we found that C6orf223 was dimerized through disulfide bonds (Supplemental Figure 3, G and H), which likely facilitates the assembly of the PRMT5-MEP50 hetero-dimer into the PRMT5-MEP50 hetero-octamer. We then investigated how the disulfide bonds are formed. C6orf223 contains 2 cysteines (C23 and C178). We mutated the cysteines separately or the 2 cysteines together (Figure 3F). The mutants were tagged with HA (HA1, HA2, and HA1+2) or His (His1, His2, and His1+2) and coexpressed in HEK293T. Co-IP showed that C23 mutation (HA1 or His1) did not influence C6orf223 dimerization. In contrast, C6orf223-HA and C6orf223-His failed to interact after C178 was mutated (HA2 or His2) (Figure 3G). Thus, C6orf223 is dimerized through C178-mediated disulfide bonds. We further observed that C178 mutation failed to increase PRMT5-MEP50 multiprotein complex and SDMA level (Figure 3E), suggesting that C6orf223 facilitates PRMT5-MEP50 multiprotein complex formation dependent on C6orf223 dimerization through disulfide bonds.

On the basis of the above observations and molecular docking, we constructed a model demonstrating how C6orf223 facilitates PRMT5-MEP50 multiprotein complex assembling (Figure 3H). In the illustrated model, the PRMT5-MEP50 hetero-octamer model is shown on the right and the putative molecular docking model between C6orf223 and PRMT5 is shown on the left (Figure 3H). In the hetero-octamer, one PRMT5 monomer is shown in green; the violet part is the region of negatively charged residues in the C-terminal of PRMT5, and the orange part is the arginine-rich region with a strong positive charge of C6orf223. The lines 1, 2, and 3 represent 3 possibilities that C6orf223 dimers bind to the PRMT5 to facilitate PRMT5-MEP50 hetero-octamer assembling. Thus, C6orf223 dimer binds to the negatively charged groove of the C-terminal of PRMT5 through its N-terminal arginine-rich region, thereby strengthening the PRMT5 complex (Figure 3H).

### C6orf223 promotes CRC metastasis.

**Figure 4:**
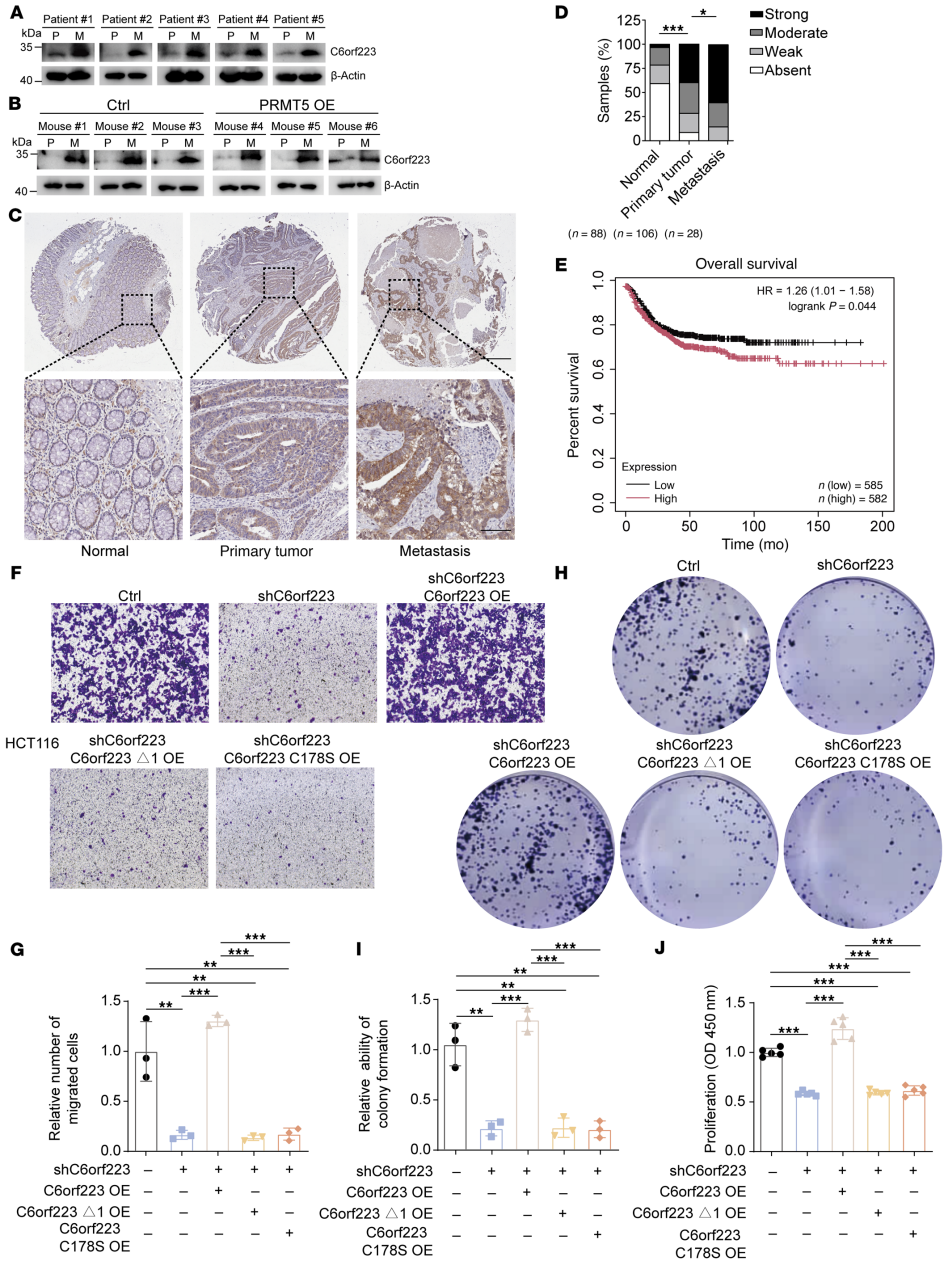
C6orf223 promotes the proliferation and migration of CRC cells in vitro. (**A**) Western blot showing the expression levels of C6orf223 in paired primary CRC (P) and liver metastatic samples (M). (**B**) Western blot showing the expression levels of C6orf223 in paired primary tumors (P) and liver metastases (M) from NCG mice orthotopically injected with HT29 cells with empty vector (Ctrl) or ectopic PRMT5 expression (PRMT5 OE). (**C** and **D**) Representative IHC images (**C**) and quantification (**D**) of C6orf223 levels in a tissue microarray. *P* value was calculated by χ^2^ test (***P < 0.001; *P < 0.05). Upper scale bar: 500 μm; lower scale bar: 100 μm. (**E**) Kaplan-Meier analysis of survival curve of patients with CRC with tumors expressing low (*n* = 471) and high (*n* = 126) C6orf223 expression characterized by TCGA database. *P* value was calculated by log-rank test. (**F** and **G**) Migration assay of HCT116 cells with C6orf223 knockdown rescued by full-length C6orf223, C6orf223 lacking N-terminal arginine-enriched region (C6orf223 Δ1), or C6orf223 with C178 mutation (C6orf223 C178S). Data are shown as the mean ± SD of 3 independent repeated experiments. *P* value was calculated by 2-way ANOVA (**P < 0.01, ***P < 0.001). (**H** and **I**) Colony formation of HCT116 cells with C6orf223 knockdown rescued by full-length C6orf223, C6orf223 lacking N-terminal arginine-enriched region (C6orf223 Δ1), or C6orf223 with C178 mutation (C6orf223 C178S). Data are shown as the mean ± SD of 3 independent repeated experiments. *P* value was calculated by 2-way ANOVA (**P < 0.01, ***P < 0.001). (**J**) Proliferation of HCT116 cells with C6orf223 knockdown rescued by full-length C6orf223, C6orf223 lacking N-terminal arginine-enriched region (C6orf223 Δ1), or C6orf223 with C178 mutation (C6orf223 C178S). Data are shown as the mean ± SD of 3 independent repeated experiments. *P* value was calculated by 2-way ANOVA (***P < 0.001).

We next investigated C6orf223 expression in normal colon, primary CRC, and CRC liver metastases. We found that C6orf223 expression levels were substantially higher in tumor tissues compared with normal colon tissues (Supplemental Figure 4, A and B). In addition, C6orf223 expression was marked elevated in liver metastases collected from patients with CRC and in the orthotopic mouse model, consistent with the observations that higher levels of PRMT5-MEP50 multiprotein complex in liver metastases and C6orf223 facilitate PRMT5-MEP50 formation (Figure 4, A and B). We further examined C6orf223 expression levels on the tissue array containing 25 pair-matched primary CRC and liver metastatic samples. Consistently, we found that C6orf223 expression levels were much higher in liver metastases compared with primary CRC (Figure 4, C and D). In addition, the survival curve generated by the Kaplan-Meier plotter, which integrates data from 17 GEO datasets, showed that high expression level of C6orf223 was associated with poor prognosis of patients with CRC as well (Figure 4E) (45).

**Figure 5:**
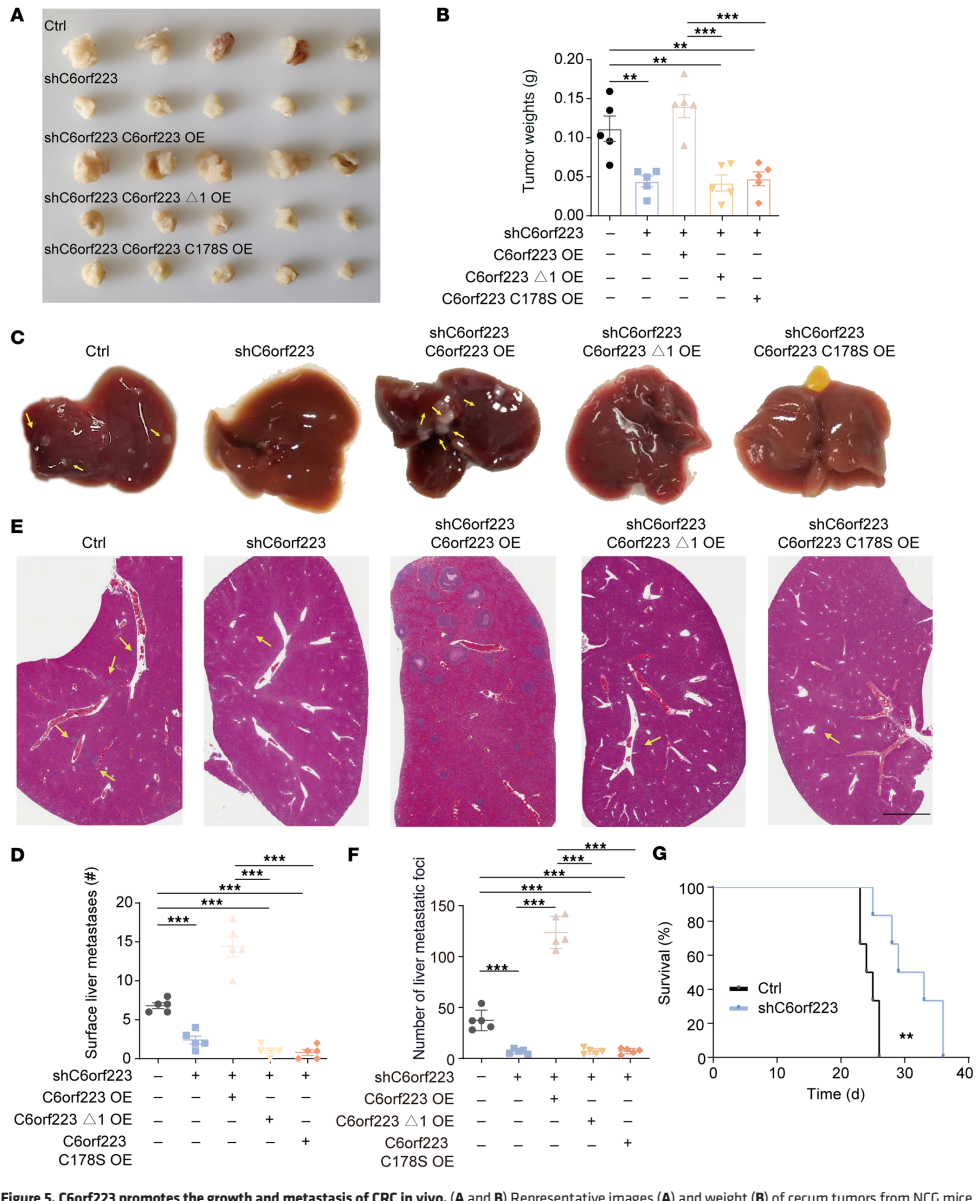
C6orf223 promotes the growth and metastasis of CRC in vivo. (**A** and **B**) Representative images (**A**) and weight (**B**) of cecum tumors from NCG mice orthotopically injected with HCT116 cells. Data are shown as the mean ± SEM of 5 mice per group. *P* value was calculated by 2-way ANOVA (***P < 0.001, **P < 0.01). (**C** and **D**) Representative images (**C**) and quantification (**D**) of the liver metastases in NCG mice orthotopically injected with HCT116 cells. Less conspicuous liver metastases are indicated by yellow arrows. Data are shown as the mean ± SEM of 5 mice per group. *P* value was calculated by 2-way ANOVA (***P < 0.001). (**E** and **F**) Representative H&E staining (**D**) and quantification (**F**) of the liver metastases in NCG mice orthotopically injected with HCT116 cells. Less conspicuous liver metastases are indicated by yellow arrows. Data are shown as the mean ± SEM of 5 mice per group. Scale bar: 2 mm. *P* value was calculated by 2-way ANOVA (***P < 0.001). (**G**) Survival curve analysis of NCG mice with cecum injection of HCT116 cells. *P* value was calculated by log-rank test.

We then investigated the influence of C6orf223 on CRC progression. We observed that ectopic C6orf223 expression in HT29 cells enhanced migration and colony formation of the CRC cells, which was abrogated when the N-terminal arginine-rich region of C6orf223 was deleted, or with a C178 mutation in C6orf223, or when PRMT5 was knocked down (Supplemental Figure 4, C–G). We further introduced full-length C6orf223, C6orf223 lacking the N-terminal arginine-enriched region (C6orf223 Δ1), or C6orf223 with a C178 mutation (C6orf223 C178S) into HCT116 cells with C6orf223 knockdown. Knockdown of C6orf223 suppressed cell migration, colony formation, and proliferation, which was reversed by restoring full-length C6orf223 but not C6orf223 Δ1 and C6orf223 C178S mutants (Figure 4, F–J). However, C6orf223 knockdown or overexpression did not change the location of PRMT5 in CRC cells (Supplemental Figure 5, A and B). An orthotopic mouse model further showed that C6orf223 promoted CRC growth and liver metastasis through its N-terminal arginine-enriched region and C178 (Figure 5, A–F, and Supplemental Figure 6, A and B). In addition, the spleen injection model showed that knockdown of C6orf223 suppressed liver metastasis, which was reversed by restoring full-length C6orf223 but not C6orf223 Δ1 and C6orf223 C178S mutants (Supplemental Figure 5, C and D). Moreover, knockdown of C6orf223 prolonged the survival of tumor-bearing mice, and C6orf223 overexpression shortened the survival of tumor-bearing mice (Figure 5G and Supplemental Figure 5E).

### C6orf223 suppresses GATA5 expression through PRMT5-mediated H4R3me2s.

**Figure 6:**
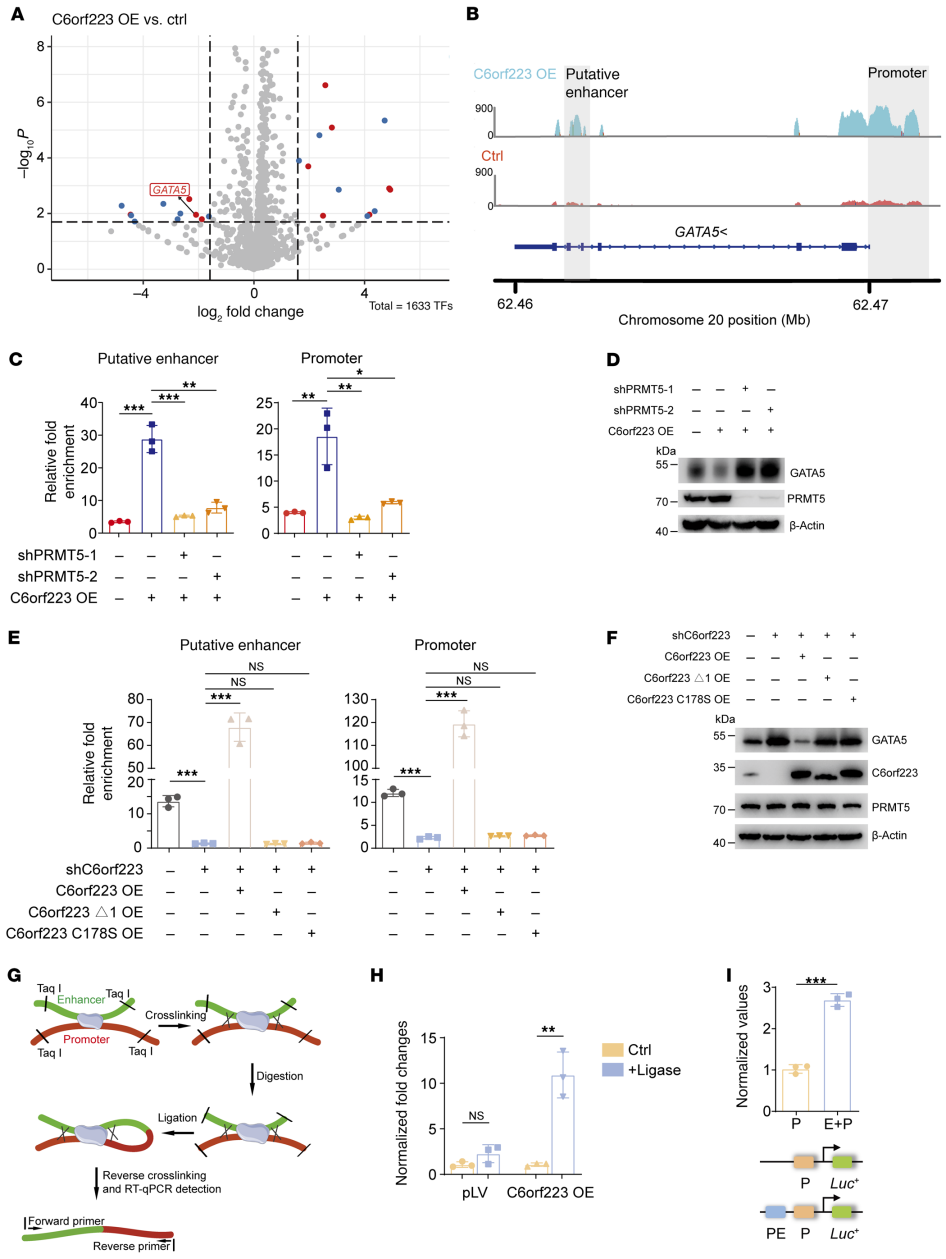
C6orf223 suppresses GATA5 expression through PRMT5-mediated H4R3me2s. (**A**) A volcano plot showing differentially expressed transcription factors in HT29 cells with empty vector (Ctrl) or ectopic C6orf223 expression (C6orf223 OE). Genes marked with blue are not associated with cancer or logically ill conceived; genes marked with red are cancer-related genes. (**B**) Genome browser snapshots of H4R3me2s at the loci of the promoter and the putative enhancer of *GATA5* in HT29 cells. (**C** and **D**) ChIP-qPCR showing the enrichment of H4R3me2s (**C**) and Western blot showing the levels (**D**) of GATA5 in HT29 cells with ectopic C6orf223 expression and/or PRMT5 knockdown. Data are shown as the mean ± SD of 3 independent repeated experiments. *P* value was calculated by 2-way ANOVA (*P < 0.05, **P < 0.01, ***P < 0.001). (**E** and **F**) ChIP-qPCR showing the enrichment of H4R3me2s (**E**) and Western blot showing the levels (**F**) of GATA5 in HCT116 cells with C6orf223 knockdown rescued by C6orf223 mutants. Data are shown as the mean ± SD of 3 independent repeated experiments. *P* value was calculated by 2-way ANOVA (NS, ***P < 0.001). (**G**) Schematic of chromatin conformation capture experiment (3C). (**H**) Interactions between putative enhancer and promoter of *GATA5* detected by 3C in HT29 cells with or without ectopic C6orf223 expression. Data were presented as fold enrichment over 3C samples without adding T4 ligase after normalization to input. Data are shown as the mean ± SD of 3 independent repeated experiments. *P* value was calculated by 2-tailed paired Student’s *t* test (NS; **P < 0.01). (**I**) Luciferase reporter activities driven by promoter and putative enhancer of *GATA5*. HT29 cells were transfected with vector containing promoter only (P) and/or putative enhancer (PE). Data are shown as the mean ± SD of 3 independent repeated experiments. *P* value was calculated by 2-tailed paired Student’s *t* test (***P < 0.001).

We showed that C6orf223 enhanced PRMT5-mediated enrichment of H4R3me2s (Figure 2, F and I, and Supplemental Figure 3E), while the enrichment of H4R3me2s often causes downregulation of the associated genes (42). Therefore, we speculated that H4R3me2s-associated genes might contribute to C6orf223/PRMT5 axis–mediated CRC progression. To investigate the target genes, we performed RNA-Seq by ectopically expressed C6orf223 in HT29 cells. Although ectopic C6orf223 expression affected RNA alternative splicing to some degree, it markedly downregulated the expression of many transcription factors. (Figure 6A and Supplemental Figure 7A). We speculate that C6orf223/PRMT5 promotes tumor growth and metastasis by regulating multiple genes, likely through transcription factors, rather than just one gene. This may explain the strong promoting effect of C6orf223/PRMT5 on CRC growth and metastasis. Considering that the increased enrichment of H4R3me2s mediated by C6orf223 leads to the suppression of gene expression, we focused on 3 transcription factors, HOXD13, GATA5, and ELF5, whose expression levels were reduced after C6orf223 overexpression. ChIP-Seq results showed that the enrichment of H4R3me2s on GATA5 was substantially increased with C6orf223 overexpression, while the enrichment of H4R3me2s on the other 2 candidate genes remained unchanged (Figure 6B and Supplemental Figure 7, B and C). Therefore, we focused on GATA5, a tumor suppressive transcription factor, although its targets remain elucidated. ChIP-Seq of H4R3me2s revealed that C6orf223 enriched H4R3me2s in both the promoter and gene body region of *GATA5* (Figure 6B). ChIP-qPCR further confirmed that C6orf223 enhanced the enrichment of H4R3me2s on *GATA5* (Figure 6C and Supplemental Figure 7, D and E). Furthermore, the effect of C6orf223 required PRMT5 (Figure 6C). Consistently, C6orf223 through PRMT5 suppressed GATA5 expression in CRC cells (Figure 6D and Supplemental Figure 7F). Considering that C6orf223 strengthens PRMT5-MEP50 multiprotein complex and activity through its N-terminal arginine-enriched region and C178-mediated disulfide bonds (Figure 3, E and H), we then ectopically expressed C6orf223, C6orf223 lacking the N-terminal arginine-enriched region (C6orf223 Δ1), and C6orf223 with a C178 mutation (C6orf223 C178S) into C6orf223-knocked-down CRC cells. ChIP-qPCR showed that C6orf223 Δ1 and C6orf223 C178S failed to increase the enrichment of H4R3me2s on *GATA5* (Figure 6E), in contrast to C6orf223, which markedly enriched H4R3me2s levels (Figure 2I and Figure 3E). Furthermore, C6orf223 Δ1 and C6orf223 C178S failed to downregulate GATA5 expression as well (Figure 6F).

H4R3me2s in the promoter region has been well studied (28, 29, 46, 47), whereas H4R3me2s in the gene body regions remains largely unknown but likely acts as an enhancer. C6orf223 enhanced the enrichment of H4R3me2s on the *GATA5* gene body region (putative enhancer) in addition to its promoter (Figure 6B). We performed chromatin conformation capture (3C) and found that the putative enhancer region was sterically bound to *GATA5* promoter (Figure 6, G and H). We further cloned the putative enhancer region into pGL3, a commonly used vector for the dual-luciferase reporter system. Compared with the plasmid containing only the promoter, the putative enhancer region significantly increased luciferase activity (Figure 6I). Taken together, the results show that C6orf223 suppresses GATA5 expression through PRMT5-mediated H4R3me2s.

### C6orf223 promotes CRC metastasis through manipulating GATA5 and its target genes.

**Figure 7:**
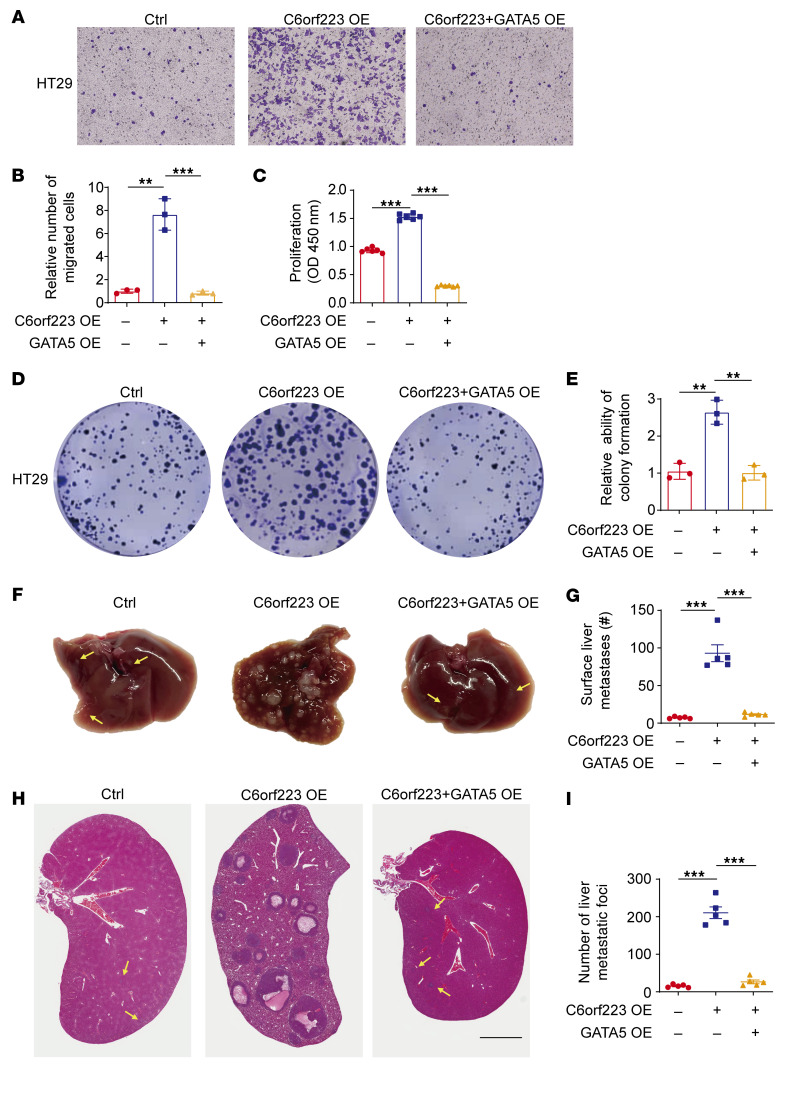
C6orf223 promotes CRC growth and metastasis through GATA5. (**A** and **B**) Representative image (**A**) and quantification (**B**) of migration assay of HT29 cells with ectopic expression of C6orf223 or C6orf223 together with GATA5. Data are shown as the mean ± SD of 3 independent repeated experiments. *P* value was calculated by 1-way ANOVA (**P < 0.01, ***P < 0.001). (**C**) Proliferation of HT29 cells with ectopic expression of C6orf223 or C6orf223 together with GATA5. Data are shown as the mean ± SD of 3 independent repeated experiments. *P* value was calculated by 1-way ANOVA (***P < 0.001). (**D** and **E**) Representative image (**D**) and quantification (**E**) of colony formation of HT29 cells with ectopic expression of C6orf223 or C6orf223 together with GATA5. Data are shown as the mean ± SD of 3 independent repeated experiments. *P* value was calculated by 1-way ANOVA (**P < 0.01). (**F** and **G**) Representative images (**F**) and quantification (**G**) of the metastases in NCG mice orthotopically injected with HT29 cells. Less conspicuous metastases are indicated by yellow arrows. Data are shown as the mean ± SEM of 5 mice per group. *P* value was calculated by 1-way ANOVA (***P < 0.001). (**H** and **I**) Representative H&E staining (**H**) and quantification (**I**) of CRC liver metastases in NCG mice orthotopically injected with HT29 cells. Less conspicuous liver metastases are indicated by yellow arrows. Data are shown as the mean ± SEM of 5 mice per group. Scale bar: 2 mm. *P* value was calculated by 1-way ANOVA (***P < 0.001).

To investigate whether suppressing GATA5 expression accounts for C6orf223/PRMT5 axis–enhanced CRC growth and metastasis, we ectopically expressed both C6orf223 and GATA5 in CRC cells. We found that C6orf223 upregulated CRC cell migration, proliferation, and colony formation, which were abrogated by restoring GATA5 expression (Figure 7, A–E). Moreover, the orthotopic mouse model showed that ectopic GATA5 expression inhibited C6orf223-enchanced CRC growth and metastasis (Figure 7, F–I, and Supplemental Figure 7, G and H).

**Figure 8:**
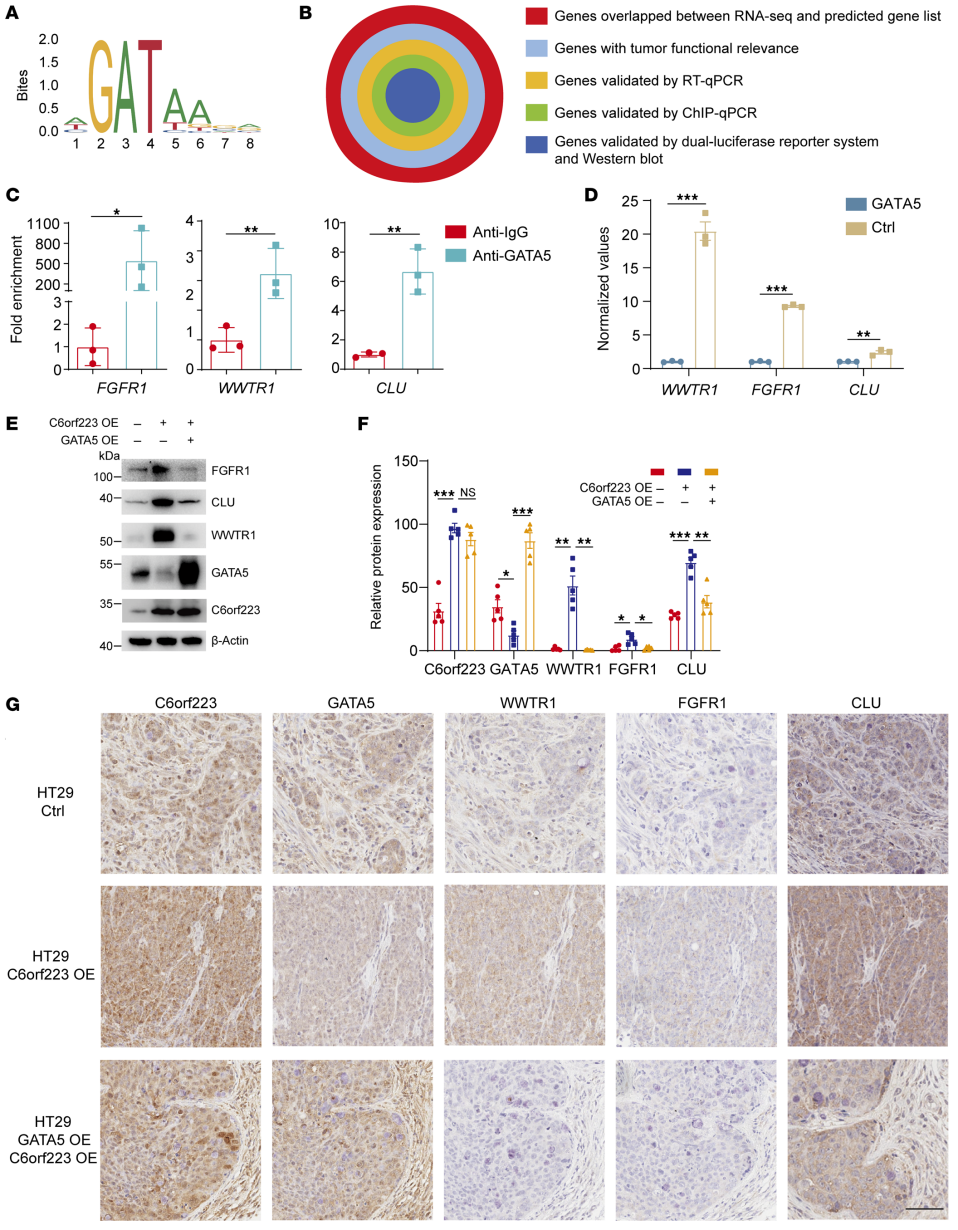
Identification of GATA5 target genes in response to C6orf223-mediated CRC growth and metastasis. (**A**) Binding motif of GATA5 analyzed by JASPAR. (**B**) Schematic of the strategy to screen the target genes of GATA5. (**C**) ChIP-qPCR showing the enrichment of GATA5 binding in the promoter of the putative target genes in HT29 cells with ectopic GATA5 expression. Data are shown as the mean ± SD of 3 independent repeated experiments. *P* value was calculated by 2-way ANOVA (*P < 0.05, **P < 0.01). (**D**) Luciferase reporter assay of the putative GATA5 target genes. HEK293T cells were transfected with luciferase vector containing promoter sequence of the putative GATA5 target genes together with GATA5 plasmids or empty vector, followed by luciferase activity measurement. Data are shown as the mean ± SD of 3 independent repeated experiments. *P* value was calculated by 2-way ANOVA (**P < 0.01, ***P < 0.001). (**E**) Western blot showing the expression levels of FGFR1, CLU, and WWTR1 in HT29 cells with ectopic expression of C6orf223 or C6orf223 together with GATA5. (**F** and **G**) Quantification (**F**) and representative IHC image (**G**) showing the expression level of C6orf223, GATA5, WWTR1, FGFR1, and CLU in primary cecum tumors from mice orthotopically injected with HT29 cells. Scale bar: 100 μm. Data are shown as the mean ± SEM of 5 mice per group. *P* value was calculated by 2-way ANOVA (NS; *P < 0.05, **P < 0.01, ***P < 0.001).

**Figure 9:**
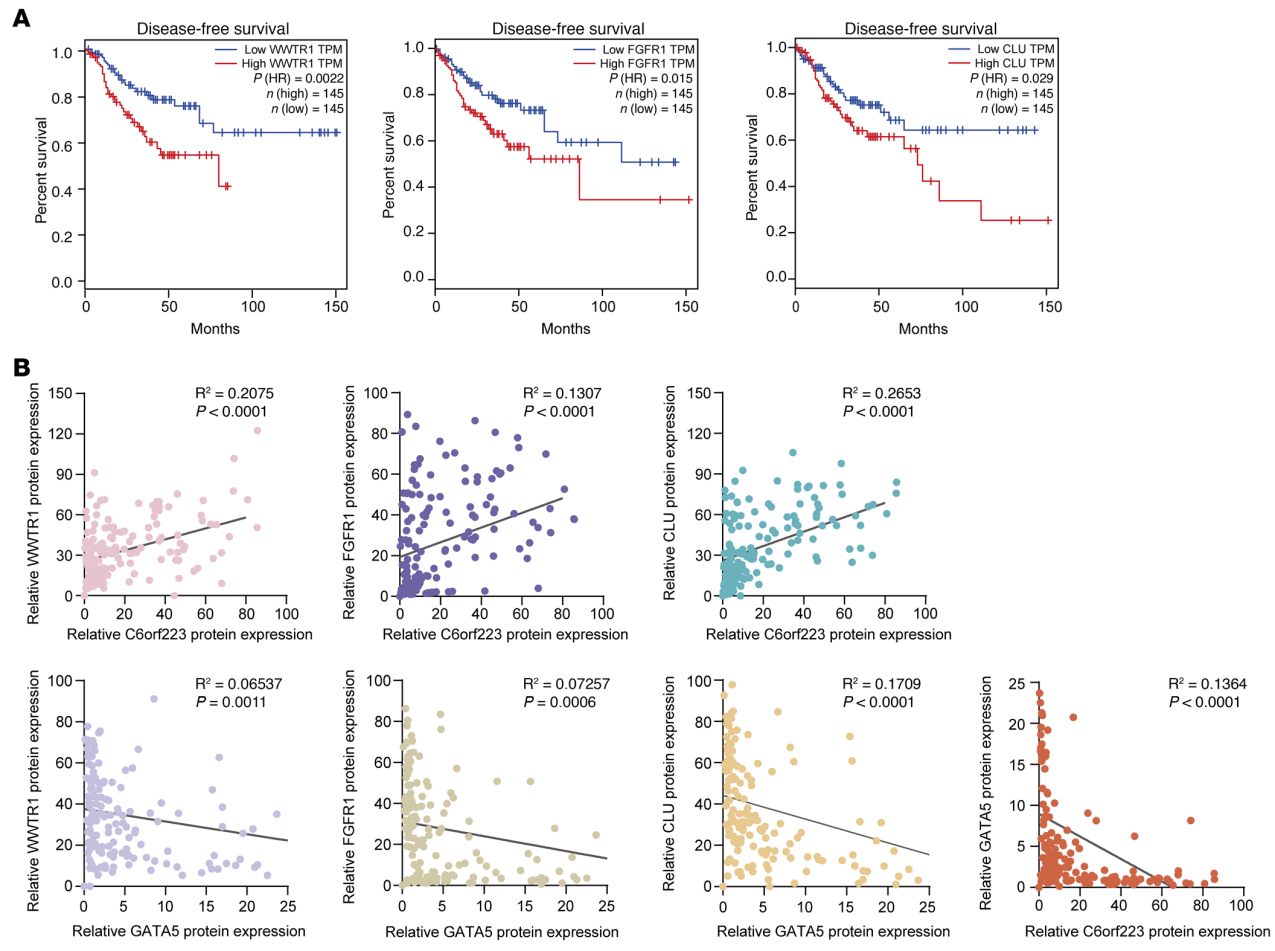
Survival analysis of GATA5 target genes and their correlation with C6orf223 and GATA5. (**A**) Kaplan-Meier analyses of disease-free survival curve of patients with CRC with tumors expressing low and high WWTR1, FGFR1, or CLU levels characterized by TCGA database. Patient data were analyzed by GEPIA. *P* value was calculated by log-rank test. (**B**) The correlation of the expression of C6orf223, GATA5, and the target genes (FGFR1, WWTR1, CLU) of GATA5 in a tissue array containing paired adenocarcinoma and metastasis of patients with CRC. *P* value was calculated by Pearson’s correlation analysis.

The above results showed that GATA5 had a conspicuous function of inhibiting CRC metastasis, although its downstream target genes have not been reported yet. GATA5 has a unique binding motif like other GATA members (Figure 8A). We then explored GATA5 target genes and its mechanism of tumor suppression through the binding motif–based prediction and series of experimental validation (Figure 8B). By performing integrated analysis of the target genes predicted by a previous method of identifying transcription binding sites and the results in the TF2DNA database (48, 49), we acquired 20 potential GATA5 target genes, which were crossed with C6orf223-regulated genes obtained by RNA-Seq. After assessment of the tumor-associated functional relevance and validation by RT-qPCR, 6 cancer-associated genes were confirmed to be regulated by GATA5 (Supplemental Figure 8A). ChIP-qPCR further confirmed that GATA5 could bind to the promoter of 5 candidates, and the luciferase reporter assay showed that 3 of them (*WWTR1*, *FGFR1*, and *CLU*) were regulated by the binding of GATA5 to their promoters (Figure 8, C and D, and Supplemental Figure 8, B and C). WWTR1, FGFR1, and CLU are well-known oncogenes that promote progression of various cancers (50–55). To investigate whether C6orf223 regulates the expression of the 3 genes through GATA5, we ectopically expressed C6orf223 and/or GATA5 in CRC cells. Western blot showed that WWTR1, FGFR1, and CLU were upregulated by C6orf223, which was reversed by overexpression of GATA5 (Figure 8E and Supplemental Figure 9A). Consistently, IHC staining showed that C6orf223/GATA5 regulated the expression of WWTR1, FGFR1, and CLU in CRC tumors as well (Figure 8, F and G, and Supplemental Figure 9, B and C). Expression levels of the genes were also strongly associated with clinical outcome. TCGA showed that patients with CRC with high expression of CLU, FGFR1, and WWTR1 had significantly shorter disease-free survival compared with patients with low expression of the genes (Figure 9A) (56). Together, the results show that oncogenes WWTR1, FGFR1, and CLU are targeted by GATA5 and upregulated by the C6orf223/GATA5 axis.

### The correlation between the levels of C6orf223 and GATA with WWTR1, FGFR1, and CLU in CRC samples.

We then investigated the correlation between the expression levels of C6orf223 and GATA5 with WWTR1, FGFR1, and CLU in clinical CRC samples. By analyzing primary CRC tumors and metastasis tissues via tissue arrays, we found that the expression of C6orf223 and GATA5 showed a converse correlation in the CRC tissues (Figure 9B). Further analyses showed that C6orf223 and WWTR1, FGFR1, and CLU were positively correlated in CRC tissues, while GATA5 and WWTR1, FGFR1, and CLU were conversely correlated in CRC tissues (Figure 9B). Taken together, C6orf223 expression is conversely associated with GATA5 and positively associated with WWTR1, FGFR1, and CLU expression in clinical CRC samples.

### Targeting C6orf223 suppresses CRC tumor growth and metastasis.

**Figure 10:**
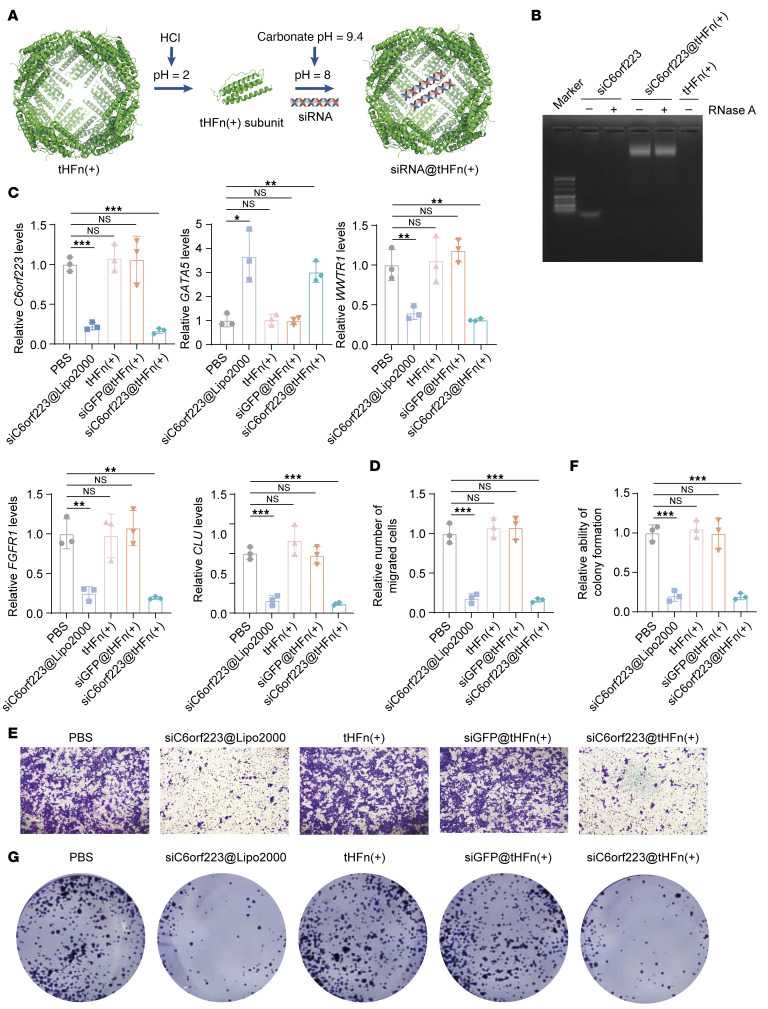
Targeting C6orf223 suppresses the proliferation and migration of CRC cells in vitro. (**A**) Schematic of the process that C6orf223 siRNAs are encapsulated in tHFn(+) protein shell via changing pH. (**B**) Agarose gel electrophoresis validating C6orf223 siRNAs encapsulated inside tHFn(+) protein shell. (**C**) RT-qPCR showing the expression levels of C6orf223, GATA5, WWTR1, FGFR1, and CLU in HCT116 cells after treatment with PBS, tFHn(+), siGFP@ tHFn(+), siC6orf223@tHFn(+), or siC6orf223@Lipo2000. Data are shown as the mean ± SD of 3 independent repeated experiments. *P* value was calculated by 2-way ANOVA (NS; *P < 0.05, **P < 0.01, ***P < 0.001). (**D** and **E**) Quantification (**D**) and representative images (**E**) of migration assay of HCT116 cells after treatment with PBS, tFHn(+), siGFP@tHFn(+), siC6orf223@tHFn(+), or siC6orf223@Lipo2000. Data are shown as the mean ± SD of 3 independent repeated experiments. *P* value was calculated by 2-way ANOVA (NS; ***P < 0.001). (**F** and **G**) Quantification (**F**) and representative images (**G**) of colony formation of HCT116 cells after treatment with PBS, tFHn(+), siGFP@tHFn(+), siC6orf223@tHFn(+), or siC6orf223@Lipo2000. Data are shown as the mean ± SD of 3 independent repeated experiments. *P* value was calculated by 2-way ANOVA (NS; ***P < 0.001).

Based on the aforementioned findings, we hypothesized that targeting C6orf223 could represent a unique strategy for CRC treatment. To explore this possibility, we first analyzed the expression of C6orf223 in normal tissues in the GEPIA2 database (Supplemental Figure 10A). C6orf223 was negative in most normal tissues and had low expression in the lung and kidney (Supplemental Figure 10A). But C6orf223 was highly expressed in several tumors, especially CRC (Supplemental Figure 10B). We then developed siRNAs specific to C6orf223 and encapsulated them within ferritin shells by adjusting the pH (Figure 10, A and B, and Supplemental Figure 10C) (57). Ferritin, a prominent iron storage protein, features a hollow interior cavity that makes it suitable for drug delivery. The ferritin nanocage offers significant advantages, such as low toxicity and immunogenicity due to its natural occurrence in humans. Moreover, ferritin exhibits high affinity for transferrin receptor 1 (TfR1), which is often overexpressed in cancer cells (58). We validated that TfR1 was indeed highly expressed in cancer cells (Supplemental Figure 10D) and 50 μg/mL C6orf223 siRNA-encapsulated with ferritin protein shell (siC6orf223@tHFn(+)) decreased the expression of C6orf223 (Supplemental Figure 10E). As expected, siC6orf223@tHFn(+) decreased the expression of WWTR1, FGFR1, and CLU and increased the expression of GATA5 (Figure 10C). Functionally, siC6orf223@tHFn(+) suppressed migration and colony formation of CRC cells in vitro (Figure 10, D–G).

**Figure 11:**
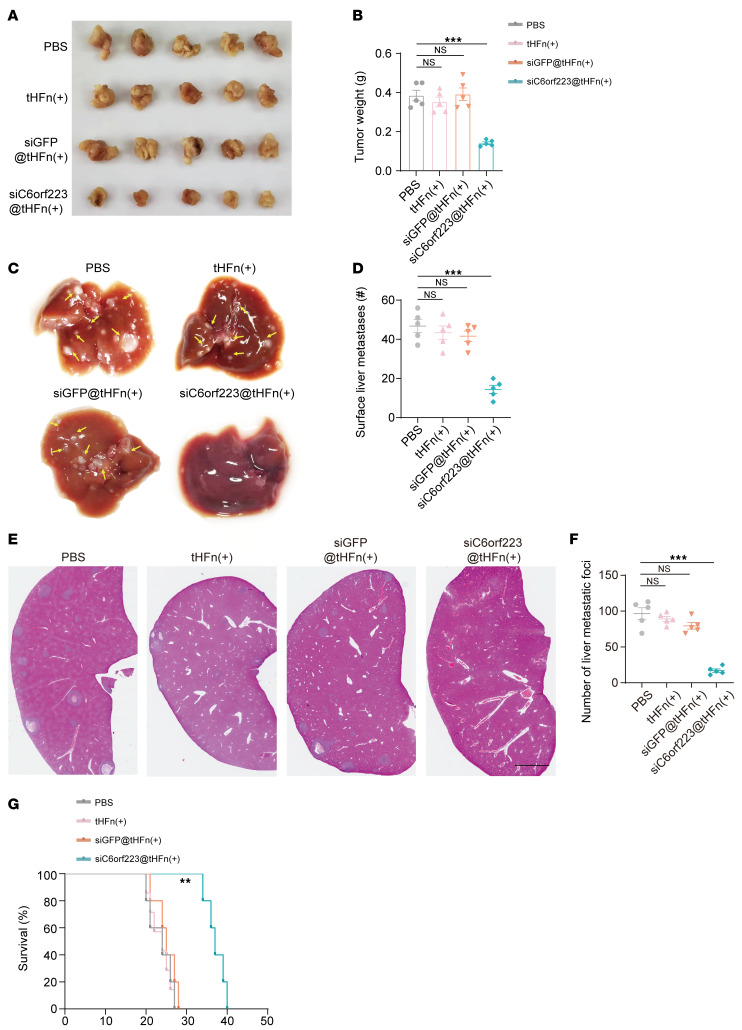
Targeting C6orf223 suppresses CRC tumor growth and metastasis in vivo. (**A** and **B**) Representative images (**A**) and weight (**B**) of cecum tumors in NCG mice orthotopically injected with HCT116 cells. The mice were treated with PBS, tHFn(+), siGFP@tHFn(+), or siC6orf223@tHFn(+). Data are shown as the mean ± SEM of 5 mice per group. *P* value was calculated by 2-way ANOVA and 2-tailed paired Student’s *t* test (NS, ***P < 0.001). (**C** and **D**) Representative bright-field images (**C**) and quantification (**D**) of the metastases in NCG mice with cecum injection of HCT116 cells. The mice were treated with PBS, tHFn(+), siGFP@tHFn(+), or siC6orf223@tHFn(+). Data are shown as the mean ± SEM of 5 mice per group. *P* value was calculated by 2-way ANOVA and 2-tailed paired Student’s *t* test (NS, ***P < 0.001). (**E** and **F**) Representative H&E staining (**E**) and quantification (**F**) of the metastases in NCG mice with cecum injection of HCT116 cells. The mice were treated with PBS, tHFn(+), siGFP@tHFn(+), or siC6orf223@tHFn(+). Data are shown as the mean ± SEM of 5 mice per group. Scale bar: 2 mm. *P* value was calculated by 2-way ANOVA and 2-tailed paired Student’s *t* test (NS, ***P < 0.001). (**G**) Survival curve analysis of NCG mice with cecum injection of HCT116 cells. The mice were treated with PBS, tHFn(+), siGFP@tHFn(+), or siC6orf223@tHFn(+). *P* value was calculated by log-rank test.

Next, we investigated the biodistribution and biosafety of siC6orf223@tHFn(+). We intravenously injected PBS, tHFn(+), cy5-siGFP@tHFn(+), and cy5-siC6orf223@tHFn(+) into HCT116-bearing mice. Ex vivo fluorescent images showed a significant fluorescence signal in tumors of the mice treated with cy5-siGFP@tHFn(+) and cy5-siC6orf223@tHFn(+), suggesting siC6orf223@tHFn(+) accumulated in the tumors (Supplemental Figure 10F). In addition, the potential toxic effects of siC6orf223@tHFn(+) on normal tissues were evaluated in healthy BALB/c mice. The major organs in mice treated with siGFP@tHFn(+) and siC6orf223@tHFn(+) maintained typical physiologic morphology and did not manifest significant histopathological abnormalities or lesions (Supplemental Figure 11). Taken together, the results demonstrate that the siC6orf223@tHFn(+) nanocarrier accumulates in the tumor and exhibits excellent biosafety. The orthotopic mouse model showed that siC6orf223@tHFn(+) decreased CRC growth and liver metastasis in vivo (Figure 11, A–F). IHC staining showed that knockdown of C6orf223 upregulated the expression of GATA5 and downregulated the expression of WWTR1, FGFR1, and CLU in tumors (Supplemental Figure 12, A and B). Moreover, siC6orf223@tHFn(+) treatment prolonged the survival of tumor-bearing mice (Figure 11G).

## Discussion

In this study, we discovered that C6orf223, to our knowledge an uncharacterized protein, plays an important role in CRC growth and metastasis. The expression level of C6orf223 is low in normal colon tissues and gradually becomes elevated from primary CRC to liver metastases. C6orf223 enhances CRC growth and metastases through suppression of the transcription factor GATA5. Mechanistically, C6orf223 binds to PRMT5 to stabilize PRMT5-MEP50 hetero-octameric complex, thereby increasing PRMT5 activity and the enrichment of H4R3me2s on *GATA5*. PRMT5-MEP50 hetero-octameric complex is essential for the arginine dimethyltransferase activity of PRMT5, and the complex is dynamically altered from PRMT5 monomer, PRMT5 tetramer, and PRMT5-MEP50 hetero-octamer. Although C6orf223 might not be required for PRMT5-MEP50 hetero-octamer assembling, it helps to stabilize the PRMT5-MEP50 hetero-octamer, which we concluded by a series of experiments. First, we verified that C6orf223 interacts with PRMT5 but not its partner MEP50 by co-IP. The interaction of C6orf223 and PRMT5 was further validated in living cells by FRET assay. Second, truncation analysis revealed that C6orf223 interacts with PRMT5 through its N-terminal arginine-rich region and PRMT5 C-terminal negatively charged groove. Third, C6orf223-enhanced PRMT5-MEP50 hetero-octamer requires dimerization of C6orf223, and we identified the cysteine residues required for the formation of the intermolecular disulfide bond of C6orf223. Last, C6orf223-enhanced PRMT5-MEP50 hetero-octamer increases the arginine dimethyltransferase activity of PRMT5, thereby suppressing GATA5 expression. To understand the mechanism in more detail, we tried to acquire the structure of PRMT5/MEP50/C6orf223 complex by expressing the proteins in either prokaryotic or eukaryotic cells. Unfortunately, C6orf223 is difficult to purify from either cell likely due to lack of stability of the protein. PRMT5 is the major symmetric arginine dimethyltransferase in mammalian cells, catalyzing various diverse substrates, including arginine residues within histone proteins and nonhistone proteins (5, 42). In addition to PRMT5-mediated H4R3me2s and H3R8me2s, which mainly causes transcriptional suppression of genes, PRMT5-mediated arginine symmetrical dimethylation on H3R2 is generally associated with transcriptional activation of genes (59, 60). In addition, some nonhistone proteins, including tumor suppressors or oncogenes, have been reported as PRMT5 substrates (61–64). In this study, we revealed that C6orf223 enhances the arginine dimethyltransferase activity of PRMT5, which likely results in arginine methylations in other histone proteins and nonhistone proteins in addition to H4R3me2s. Although we cannot rule out the possibility that other methylated histone or nonhistone proteins contribute to C6orf223-mediated tumor growth and metastasis, we found that C6orf223 enhances the enrichment of H4R3me2s on a series of genes by stabilizing the PRMT5-MEP50 hetero-octameric complex. Among these genes, C6orf223 enhances the enrichment of H4R3me2s on the *GATA5* promoter and enhancer though PRMT5, thereby suppressing GATA5 expression. GATA5 is a tumor suppressor; its CpG island has been reported to be methylated in serval cancer types (35, 65–67). PRMT5-mediated H4R3me2s also links with DNA methylation, which recruits DNMT3A through its PHD motif, thereby silencing gene expression (46). We have not investigated *GATA5* CpG island methylation in this study, and it is possible that *GATA5* CpG methylation is enhanced by C6orf223-mediated H4R3me2s. GATA5 is also a transcription factor (31, 68). Therefore, C6orf223 likely regulates several genes associated with CRC progression through GATA5, which might explain the potent cancer-promoting function of C6orf223. In this study, we indeed identified 3 previously unreported targets of GATA5 — WWTR1, CLU, and FGFR1 — which are important oncogenes and upregulated by the C6orf223/GATA5 axis. Besides regulating the expression of genes via PRMT5-mediated H4R3me2s, overexpression of C6orf223 also affects the alternative splicing events in CRC cells, indicating that C6orf223 takes part in the alternative splicing function of PRMT5. In summary, C6orf223 promotes CRC liver metastasis by various mechanisms that need to be studied in the future.

In previous studies, PRMT5 reportedly regulated AKT and TGF signaling via methylation to promote tumor metastasis (69, 70). PRMT5 also plays a role in RNA alternative splicing (8, 71). However, the regulatory mechanism of methyltransferase activity of PRMT5 in tumor metastasis is not clear. In contrast to previous studies, we revealed a mechanism by which C6orf223 enhances the methyltransferase activity of PRMT5 via facilitating PRMT5-MEP50 multiprotein complex assembling, which is supplementary to the upstream regulation of PRMT5 in tumor metastasis. Furthermore, there is an increasing interest in the pharmacological targeting of PRMT5 in tumor treatment. PRMT5 is expressed in various normal tissues and plays an important role in the normal physiology; inhibition of PRMT5 in normal tissues may lead to side effects, such as thrombocytopenia, anemia, and neutropenia (72). C6orf223 is highly expressed in several tumors, especially in CRC, is negative in most normal tissues, and has very low expression in the lung and kidney. Thus, C6orf223 could serve as a potential therapeutic target for CRC. In this study, we investigated the biosafety of siC6orf223@tHFn(+) for 8 weeks, but long-term biosafety still needs to be investigated.

## Methods

### Sex as a biological variable.

Our study examined female mice, and sex was not considered as a biological variable.

### Antibodies.

The antibody against C6orf223 was generated in a rabbit using the synthetic peptide VSGRKNSTSKDLVT as an antigen. The antibody used for IP and IHC was custom-made by GL Biochem. Antibodies specific for PRMT5 (ab109451), H4 (ab177840), MEP50 (ab190361), Myc-tag (rabbit, ab9106), and FGFR1 (ab76464) were purchased from Abcam. The C6orf223 antibody (HPA046117) used for Western blot was from Sigma-Aldrich. Antibodies specific for WWTR1 (catalog 72804), FGFR1(catalog 9740), HA-tag (mouse, 2367), and symmetric dimethyl arginine motif (sdme-RG; 13222) were obtained from Cell Signaling Technology. Antibodies specific for His-tag (AE003), Myc-tag (mouse, AE010), β-actin (AC026), GAPDH (AC033), and DDDDK (AE063) were purchased from ABclonal. The antibody against β-tubulin(A01030) was from Abbkine. The antibody specific for GATA5 (AF2170) was obtained from R&D Systems. The antibody specific for H4R3me2s (catalog 61988) was from Active Motif. The antibody specific for CLU (D262659) was obtained from Sangon. The antibody specific for anti–HA-tag (rabbit, 30702ES60) was obtained from Yeasen.

### Mice.

All animal studies in this study were approved by the Biomedical Research Ethics Committee of the Institute of Biophysics, Chinese Academy of Sciences. The experimental procedures were performed by following the relevant ethical regulations regarding animal research. NOD/ShiLtJGpt-*Prkdc^em26Cd52^Il2rg^em26Cd22^*/Gpt (NCG) mice were purchased from Gem Pharmatech Corporation.

### Cell culture, lentiviral vector, and infection.

Human CRC cell lines HCT116, HT29, SW480, SW620, and DLD1 were obtained from American Type Culture Collection (ATCC). Patient-derived xenograft CRC cell lines CRC57 and CRC119 were obtained as described previously (73). All CRC cell lines were cultured in RPMI medium 1640 basic (C11875500BT, Gibco) supplemented with 10% FBS (ST30-3302, PAN-Seratech) and 1% penicillin-streptomycin solution (SV30010, Cytiva). HEK293T cells were cultured in DMEM basic (C11965500BT, Gibco) supplemented with 10% FBS and 1% penicillin-streptomycin solution. Gibco FreeStyle 293-F cells from Thermo Fisher Scientific were cultured in OPM-293 CD05 medium (81075-001) supplemented with 1% penicillin-streptomycin solution. All adherent cell lines were cultured at 37°C with 5% CO_2_ in a cell incubator, and 293-F cells were cultured in 37°C incubator containing a humidified atmosphere of 8% CO_2_ on an orbital shaker platform rotating at 135 rpm.

shRNAs against PRMT5 and C6orf223 were cloned into the lentiviral vector pLKO.1. WT, and the different truncates of PRMT5 and C6orf223 were cloned into the lentiviral vector pCDH. The lentiviral constructs were cotransfected with helper plasmids into HEK293T cells. The virus was collected 48 hours after transfection and used to infect the cells. shRNA sequences are shown in Supplemental Table 1. siRNAs against GFP or C6orf223 were synthesized by the T7 RNAi Transcription kit (Vazyme Biotech). siRNA sequences are shown in Supplemental Table 1.

### Patient specimens.

The primary and liver metastatic CRC specimens were obtained from the Seventh Medical Center of PLA General Hospital (Beijing, China) with informed consent from all donors. The patient information is shown in Supplemental Table 2.

### Statistics.

Statistical analyses were performed with GraphPad Prism 10.0. Animal and clinical data are shown as the mean ± SEM; other data are shown as the mean ± SD. Comparisons between groups, whose distribution was determined as the normal distribution, were performed using 2-tailed Student’s *t* tests. Comparisons between 3 groups were assessed using 1-way ANOVA. Comparisons between more than 3 groups were assessed using 2-way ANOVA. Survival curves were analyzed using Kaplan-Meier analysis or a log-rank test. Pearson’s correlation analysis was conducted to test the relationship between the expression levels of C6orf223 and GATA5 with WWTR1, FGFR1, and CLU in clinical CRC samples. Each experiment was conducted with biological replicates and repeated no less than 3 times. Mice were randomly allocated to experimental groups. *P* values less than 0.05 were considered statistically significant.

### Study approval.

All studies were approved by the ethics committee of the Seventh Medical Center of PLA General Hospital and the Institute of Biophysics, Chinese Academy of Sciences.

### Data availability.

The authors declare that all data supporting the findings of this study are available in the main text or the supplemental material, including the Supporting Data Values file. The RNA-Seq and ChIP-Seq data were deposited in the China National Center for Bioinformation (https://ngdc.cncb.ac.cn/gsa-human/browse/HRA010902).

## Author contributions

YQ, ZW, and PW contributed equally to this work. YQ, ZW, JL, and PB came up with the concept, designed the experiments, and wrote the manuscript. YQ, ZW, and PW performed the majority of the experiments. YJ, YH, and KF prepared siC6orf223@ tHFn(+) and siGFP@ tHFn(+). FB assisted with the animal experiments. MX, YA, FZ, PZ, and BZ performed data analysis. HF and PG assisted with the size exclusion chromatography experiment. YZ and JL assisted with structure analysis. GS analyzed the tissue microarray data. JD, HC, and JL provided the clinical samples.

## Supplementary Material

Supplemental data

Unedited blot and gel images

Supporting data values

## Version 1 10/15/2025

Electronic publication
