# Supplementary material for: C6orf223 promotes colorectal cancer growth and metastasis by facilitating PRMT5-MEP50 multiprotein complex assembling
Source: J Clin Invest. 2025 Oct 15;135(20):e186052. doi: 10.1172/JCI186052 (PMC12520688; doi:10.1172/JCI186052)
Supplement: Supplemental data [file jci-135-186052-s330.pdf]

Supplemental Information

C6orf223 promotes colorectal cancer growth and metastasis by facilitating PRMT5/MEP50 multiprotein complex assembling

Yufeng Qiao,[1,2,10] Zhenzhen Wu,[1,2,10*] Peng Wang,[1,2,10] Yiliang Jin,[2,3] Furong Bai,[1,2] Fei Zhang,[1,2] Yunhe An,[4] Meiying Xue,[2,5] Han Feng,[2,3] Yong Zhang,[1,2] Yaxin Hou,[2,3] Junfeng Du,[6] Huiyun Cai,[6] Guizhi Shi,[7] Bing Zhou,[2,5] Pu Gao,[2,3] Jizhong Lou,[1,2] Peng Zhang,[8] Kelong Fan[2,3*] Jinbo Liu[9*] and Pengcheng Bu[1,2,11*]

Inventory for Supplemental Information

I. Supplemental methods

II. References

III. Supplemental Figure

IV. Supplemental Table

Supplemental methods

Orthotopic injection mouse models and treatment

6 weeks old female NCG mice were used in this model. The cecum injection model of colorectal cancer was generated as described previously (1, 2). 2 x 10^6 HCT116 cells were injected into the wall of the cecum with insulin syringe carefully. Randomized groups of mice were treated with PBS, tHFn(+), siGFP@tHFn(+) or siC6orf223@tHFn(+) supplement one week after the inoculation. 1 mg/kg siGFP or siC6orf223 contained in tHFn(+), tHFn(+) or PBS was injected into mice every other day through i.v..

Spleen injection mouse models

6 weeks old female NCG mice were used in this model. Mice were anesthetized via isoflurane and the surgical site was sterilized with 75% ethanol. After draping with gauze, a small surgical incision through the skin and peritoneum was made to access the abdominal cavity and expose the spleen using slight pressure. 5 x 10^5 HCT116 or 1 x 10^5 HT29 cells in 50 μL phosphate-buffered saline (PBS) were injected into spleen with insulin syringe carefully. After grafting, the peritoneal muscle and skin were surgically closed.

Quantification of metastasis

Metastatic foci on the liver surface were qualified after the whole liver tissue was collected. And then mouse livers were fixed in 4% paraformaldehyde solution overnight and then transferred to 70% ethanol for paraffin-embedded sections. Paraffin-embedded liver tissues were serially sectioned at 5 μm thickness. Five sections containing individual liver lobe were stained with hematoxylin and eosin (H&E). Metastatic foci were qualified on the base of the H&E-stained sections. The sum of the number of metastatic foci on each liver lobe was counted as the total number of metastatic foci in individual mouse.

IVIS Imaging

HCT116 tumor-bearing animals were analyzed under isoflurane anesthesia using the IVIS Lumina 3. imaging system Cy5-labled siGFP or siC6orf223 was wrapped by tHFn(+). Mice were injected intravenously with 1 mg/kg siGFP or siC6orf223 contained in tHFn(+), tHFn(+) or PBS. At predetermined intervals (1, 3, 6 hours from the dose) the mice were anesthetized with isoflurane gas and imaged for fluorescence.

***In vivo* biosafety of siC6orf223@tHFn(+)**

The potential toxic effects of siC6orf223@tHFn(+) on normal tissues were evaluated in healthy BALB/c mice. Mice were treated with PBS, tHFn(+), siGFP@tHFn(+) or siC6orf223@tHFn(+) supplement twice every week for 8 weeks. To assess histopathological changes, major organs of mice in each group, including the heart, liver, lung, spleen, kidney and colon, were embedded, cut into slices and stained by H&E to examine the effects of siC6orf223@tHFn(+) on normal tissues.

RNA isolation and RT-qPCR

RNA was purified using TRIzol reagent (15596, Thermo Fisher) or Rapure Total RNA Kit (R4011-03, Magen) and cDNA was synthesized using HiScript IIQ Select RT SuperMix (R223, Vazyme). Real-time quantitative reverse transcription-PCR was performed using Fast SybrGreen PCR Master reagent (Q311-02, Vazyme) on the real-time PCR instrument. *β-actin* was amplified as a control. Primer sequences are listed in Supplemental Table 3. All primers were validated by amplifying a series of dilution of template cDNA.

Western Blot

Cells or homogenized tissue were lysed in RIPA lysis buffer supplemented with protease inhibitors (04693132001, Roche). After centrifugation at 13,000 rpm for 10 mins, the supernatants were collected and 5×SDS-PAGE sample buffer (E153-01, GenStar) was added to them. Protein samples were separated by 10 % SDS-PAGE and transferred to PVDF membranes. Membranes were blocked with 5% skim milk in PBS for 1 h at room temperature and incubated with primary antibodies on an orbital shaker overnight at 4 °C. After washed with PBST three times, membranes were incubated with horseradish peroxidase (HRP)-conjugated secondary antibodies for 1 h at room temperature. The target proteins were detected by West Dura Extended Duration Substrate (34076, Thermo Fisher) with chemiluminescene imaging system.

Blue Native-PAGE

The BN-PAGE assay was adapted from the user guide of Native PAGE Novex Bis-Tris Gel System (Thermo Fisher). Cells or homogenized tissue were lysed in 1×NativePAGE Sample Buffer (BN2003, Thermo Fisher) supplemented with 1% Digitonin and protease inhibitors. After centrifugation at 13,000 rpm for 30 mins at 4 °C, the supernatants were collected and UltraNuclease (20156ES25, YEASEN) was added to eliminate nucleic acid. Samples were added with $MgCl_2$ to a final concentration of 2 mM and then incubated at 37 °C for 30 mins.

Protein concentrations were determined with the Pierce BCA Protein Assay Kit (23225, Thermo Fisher). Before running gels, 5% Coomassie Brilliant Blue G-250 buffer were added to the sample to a final concentration of 0.25%. BN-PAGE was conducted in 3-12% gradient Native Page precast gels (BN1003, Thermo Fisher). All of the above procedures should be performed on ice or at 4 °C unless otherwise noted.

For Western Blot, proteins were transferred to PVDF membranes in 1×NuPAGE Transfer Buffer (NP0006-1, Thermo Fisher) after electrophoresis. Transfer was performed at 25V constant for 3 h at 4 °C. After transfer, the membranes were incubated with 8% acetic acid for 15 mins then rinsed with deionized water and air-dry the membranes. The air-dried membranes were rewet with methanol then rinse with deionized water prior to immunodetection. The following procedures were consistent with the aforementioned details.

For Coomassie Staining, the gels were place in Fix solution (40% methanol, 10% acetic acid) and microwave for 45s after electrophoresis. After solution was discarded, Coomassie Brilliant Blue R-250 solution (DE0702, BioDee) was decanted and microwave for 2 mins. After staining, destaining solution (30% ethanol, 10% acetic acid) were used and the gels were shaken on an orbital shaker until the desired background is obtained.

Immunohistochemistry

Human or mouse tissues were fixed in 4% paraformaldehyde solution overnight and then transferred to 70% ethanol and processed to paraffin embedding. Slides were deparaffinized, rehydrated and retrieved antigen through boiled for 3 mins in 10 mM pH 6.0 citrate buffer (ZLI-9064, Zsbio). Slides were blocked in 5% goat serum for 1 h at room temperature. Slides were incubated with primary antibodies overnight at 4 °C. The following day, slides were incubated with the appropriate anti-species HRP-conjugated secondary antibodies (ZSGB-BIO) for 40minsand then were stained with DAB Kit (ZLI-9017, ZSCB-BIO) and hematoxylin. Finally, slides were dehydrated and mounted with neutral resin.

Co-Immunoprecipitation

HEK293T cells were transfected with tagged proteins using Vigofect (T001, Vigorous) and harvested after 36-48 h. After cells were lysed, cell lysates were precleared with Pierce Protein A/G Magnetic Beads (88803, Thermo). For immunoprecipitation, equal amounts of cell lysates were incubated with primary antibodies overnight at 4°C, after which magnetic beads were

added and incubated for 4 h at 4°C. The magnetic beads were then boiled with 1×SDS-PAGE sample buffer. The following procedures of western blot were consistent with the aforementioned details.

Sliver staining

A gradient gel of 4-12% (P41211, LABLEAD) was used for electrophoresis. After electrophoresis, the gels were fixed in Fixative (40% ethanol, 10% acetic acid) until the nonspecific background disappeared, in which the Fixative should be substituted at set intervals. Then 30% ethanol were used to wash the gels for 10mins. The destained gels were sensitized in sensitizing solution (30% ethanol, 3.14% (w/v) sodium thiosulfate pentahydrate, 11.28% (w/v) sodium acetate trihydrate) for 30 mins. After the gels were washed three times with deionized water for 5 mins, 0.25% (w/v) $AgNO_3$ solution supplemented with 0.04% formaldehyde freshly was used to stain the gels for 20 mins. Then the gels were incubated with 2.5% (w/v) Na_2CO_3 solution supplemented with 0.02% formaldehyde until desired contrast is obtained. Finally, the reaction was quenched by washing the gels with 18.6% (w/v) ethylenediaminetetraacetic acid disodium salt solution.

Fluorescence Resonance Energy Transfer (FRET)

ECFP and EYFP are the most commonly used FRET pairs. ECFP was fused at the N-terminal of PRMT5 while EYFP was fused at the C-terminal of C6orf223. HEK293T cells were transfected with vectors expressing fusion protein. 48 h after transfection, the cells were fixed by 4% PFA in PBS at RT for 10 mins. Cells were observed using Olympus FV1000 confocal laser scanning biological microscope. The efficiency of FRET was quantified on the basis of the acceptor photobleaching as mentioned in previous articles (3) and was assessed by measuring average donor fluorescence intensities before and after photobleaching of the acceptor. The calculation formula was FRET efficiency (E) = 1-Prebleaching/Postbleaching, where Prebleaching is defined as the average fluorescence intensity of 5 pre-images of the ROI, and Postbleaching is defined as the average intensity of 5 post-images.

Size exclusion chromatography (SEC)

PRMT5 with an aminol terminal tobacco etch virus (TEV) cleavage site (GAAAACCTGTATTTTCAGGGC) and His tag (CATCATCATCATCATCAT) was cloned into pCDH vector. This plasmid was expressed in HEK293F cells with or without full-length

C6orf223 expression plasmid. Purification was conducted at 4 °C after 48-72 h. Frozen pellets were resuspended in balancing buffer (50 mM Tris, 300 mM NaCl, 10 mM imidazole, 10% glycerol with protease inhibitors) and sonicated on ice for 30 min. The homogenate was clarified by ultracentrifugation for 2 h at 36000 rpm. The protein was applied to a Ni Sepharose™ 6 Fast Flow (GE Healthcare) column and eluted with 50 mM Tris, 300 mM NaCl, 500 mM imidazole. The purified fusion protein was cleaved TEV proteinase at 4 °C overnight meanwhile the protein mixture was dialysed against 50 mM Tris, 300 mM NaCl. Then the protein mixture was concentrated to 1 mL using Ultra-15 10 K Centrifugal Filter Devices (UFC903096, Merck Millipore). SEC of the protein mixture was carried out using Superdex 200 10/300 GL gel filtration column (GE Healthcare) equilibrated in PBS at the recommended flow rate of between 0.3 mL/min and 0.5 mL/min, meanwhile the A_{280nm} was continuously monitored. The eluate at each step was identified by Coomassie bright blue to ensure the presence of the target protein.

Trans-well migration assay and treatment

4×10^4 HCT116, HT29 or SW480 cells were resuspended in serum-free medium and seeded in the upper chamber (MAMIC8S10, Merck Millipore). The lower chamber was filled with medium containing 10% FBS. 24 h later, non-migrated cells on the upper side of the filter were wiped with a swab dipped in 75% ethanol. The migrated cells on the lower side of the filter were fixed with 4% PFA then were stained with 0.5% crystal violet for 15 min. After washed with ddH_2O, the number of migrated cells on the filter were counted under a microscope. The cells were treated with 50 ug/ml tHFn (+), 50ug/ml tHFn (+) containing siGFP or siC6orf223, PBS, or lipo2000 wrapped siC6orf223.

Colony formation assay and treatment

1,000 of HCT116, HT29 or SW480 cells resuspended in 1mL growth medium were seeded in 12-well plates. The medium was replaced every three days. Thereafter, the colonies were fixed with 4% PFA for 1 h at 4 °C and stained with 0.5% crystal violet. The colonies were counted directly under a microscope. The cells were treated with 50 μg/ml tHFn (+), 50 μg/ml tHFn (+) containing siGFP or siC6orf223, PBS, or lipo2000 wrapped siC6orf223.

Cross-linking chromatin immunoprecipitation(X-ChIP)

X-ChIP was performed following the official protocol of Abcam. Briefly, the cells were cross-

linked with 1% formaldehyde for 10 min at room temperature and fixation was stopped by adding 0.125 M Glycine, followed by washing with cold PBS. After the cells were lysed, chromatin DNA was sheared to 200-500 bp average in size through sonication. Resultant was immunoprecipitated with control IgG or specific primary antibodies at 4 °C overnight, followed by incubation with Protein A/G Magnetic Beads for an additional 4 h. After washing and elution, the protein-DNA complex was reversed by heating at 65 °C overnight with RNase A added. Thereafter proteinase K was added and the protein-DNA complex was incubated at 60 °C for 1 h. The DNA was purified using MinElute PCR Purification Kit (28006, QIAGEN). ChIP-qPCR primer sequences are listed in Supplemental Table 4. Primers used are specific for regions tested and their sequences are available on request. All ChIP-qPCRs were repeated at least three times and representative results were shown.

Native chromatin immunoprecipitation(N-ChIP)

N-ChIP was performed following the protocol described previously (4). Briefly, cells were harvested and then gently lysed to obtain nuclei with 0.32 M Sucrose, 60 mM KCl, 15 mM Tris pH 7.5,15 mM NaCl, 5 mM $MgCl_2$, 0.1 mM EGTA, 0.2% IGEPAL. After centrifugation at 10,000×g for 20 min to purified nuclei, 0.05% MNase were used to digest chromatin to about 150 bp. After the digestion was terminated by adding EGTA, chromatin was extracted using EDTA at final concentration of 10 mM. The following steps referred to previously the protocol of X-ChIP.

ChIP-seq data processing and analysis

ChIP-seq raw reads were assessed for quality using FastQC, followed by adapter trimming and removal of low-quality reads with Trim Galore. High-quality reads were then aligned to the Ensembl GRCh38 reference genome using Bowtie2, and duplicate reads were removed with Picard to reduce PCR amplification bias. Peak calling was performed using MACS2, incorporating input DNA controls for background correction. For narrow peaks, the –nomodel and --extsize parameters were applied, while broad peaks were identified using the --broadoption. Peaks were assigned to the nearest genes based on the Ensembl GRCh37 annotation using ChIPseeker. Differential binding analysis was conducted with DiffBind, applying normalization and dispersion estimation within a generalized linear model (GLM) framework. Significant differential binding sites were defined by an absolute log2 fold change

greater than one and a P-value below zero point one. ChIP-seq signal intensity was visualized using deepTools, generating heatmaps and metagene profiles to illustrate binding patterns across genomic regions.

RNA sequencing data processing and analysis

RNA sequencing raw data were first assessed for quality using FastQC, followed by adapter trimming and low-quality read filtering with Trim Galore. High-quality paired-end FASTQ files were then processed and quantified using the Salmon software with the Ensembl GRCh38 transcriptome index. To improve quantification accuracy, sequence bias correction (--seqBias), GC content bias correction (--gcBias), and positional bias correction (--posBias) were applied. Quality control was performed on paired-end FASTQ files before transcript-level quantification. The resulting transcript-level quantification data were imported using the Tximport R package and summarized to gene-level counts based on the Ensembl GRCh38 transcript-to-gene mapping. Differential gene expression analysis was conducted using the edgeR package. Genes with a counts-per-million (CPM) greater than one in at least one sample were retained for downstream analysis. Normalization was performed using the trimmed mean of M-values (TMM) method. An experimental design matrix was constructed, and dispersion estimation was carried out within a generalized linear model (GLM) framework. Differential expression analysis was performed to compare HT29_C6orf223 OE and pLV groups, with significance thresholds defined as an absolute log2 fold change greater than one and a P-value below zero point one. To identify differentially expressed transcription factors (TFs), genes were filtered based on the human TF gene list. The expression patterns of differentially expressed TFs were visualized using the EnhancedVolcano package, with a significance threshold set at a log2 fold change greater than log2(3), corresponding to a fold change greater than three, and a P-value below zero point zero two. The final volcano plot was generated to illustrate the distribution of differentially expressed TFs.

Chromatin Conformation Capture(3C)

3C was performed following the protocol described previously (5). Cells were fixed by 1% formaldehyde and the crosslinking was stopped by glycine. Cells were pelleted and resuspended with lysis buffer (10 mM Tris-HCl pH8.0, 10 mM NaCl, 0.2% Igepal CA630, protease inhibitors), homogenized by douncing, and then washed and resuspended in 150μl

NEB buffer Cutsmart. Sodium dodecyl sulphate (SDS) was added and the mixtures were heated at 65 °C for 10 min to remove the protein that were not crosslinked. SDS was then quenched by adding Triton X-100. Chromatin was subsequently digested for 4 h at 65 °C by adding 400 Unites TaqI (R0149, NEB). The restriction enzymes were inactivated by addition of SDS. The digested mixtures were then treated with a ligation cocktail (1% Triton X-100, 50 mM Tris-HCl pH7.5, 10 mM $MgCl_2$, 10 mM DTT, 1 mg/ml BSA, 10 mM ATP) and then ligated with 5000 U of T4 DNA ligase for 4 h at 16 °C. Crosslinked chromatin was reversed by proteinase K and RNase A. Reaction mixtures were cooled to RT and DNA was extracted by using phase lock gel (Heavy, 50 ml, 5 PRIME) following protocol provided by manufacturer.

Purified DNA was used as template for qPCR using primers targeting tested regions. The sequence of qPCR primers was TTCCTCTCCCTCGGGTTCG (forward) and TCTTGGTCTGAGAGTGCGGA (reverse).

Dual-luciferase reporter assay

The pGL3 firefly luciferase reporters containing the promoters of target genes were generated by PCR and confirmed by sequencing. Firefly luciferase reporters were then transfected together with the pRL-TK plasmid containing the Renilla luciferase reporter gene and the plasmids containing target transcription factors. 48 h after transfection, cells were lysed with Passive Lysis Buffer (E1910, Promega). The luciferase activity was measured using a Dual Luciferase Assay system (Promega). Firefly luciferase activity was normalized to Renilla luciferase.

Preparation of siRNA@tHFn(+)

siRNA@tHFn was performed according to pH-mediated disassembly/reassembly of tHFn and prepared following previously reported procedure (6, 7). tHFn in PBS was adjust to disassembly environment of pH 2.0 by adding HCl solution in 4 °C for 30 min. Subsequently, pH 9.4 carbonate buffer premixed with siRNA was added to adjust pH value to 8.0. Protein and siRNA concentration was measured with BCA Protein Assay Kits and Qubit 4 Fluorometer respectively.

References

1. Zhang LW, Zhu ZJ, Yan HW, Wang W, Wu ZZ, Zhang F, et al. Creatine promotes cancer

metastasis through activation of Smad2/3. *Cell Metab.* 2021;33(6):1111-+.
2. Zhang L, and Bu P. Generation of an orthotopic mouse model to study colorectal cancer metastasis. *STAR Protoc.* 2021;2(4):100792.
3. Wouters FS, Bastiaens PI, Wirtz KW, and Jovin TM. FRET microscopy demonstrates molecular association of non-specific lipid transfer protein (nsL-TP) with fatty acid oxidation enzymes in peroxisomes. *EMBO J.* 1998;17(24):7179-89.
4. Alonso A, Bernstein E, and Hasson D. Histone Native Chromatin Immunoprecipitation. *Methods Mol Biol.* 2018;1832:77-104.
5. Dekker J, Rippe K, Dekker M, and Kleckner N. Capturing chromosome conformation. *Science.* 2002;295(5558):1306-11.
6. Zhang B, Chen X, Tang G, Zhang R, Li J, Sun G, et al. Constructing a nanocage-based universal carrier for delivering TLR-activating nucleic acids to enhance antitumor immunotherapy. *Nano Today.* 2022;46:101564.
7. Jin Y, Zhang B, Li J, Guo Z, Zhang C, Chen X, et al. Bioengineered protein nanocarrier facilitating siRNA escape from lysosomes for targeted RNAi therapy in glioblastoma. *Sci Adv.* 2025;11(8):eadr9266.

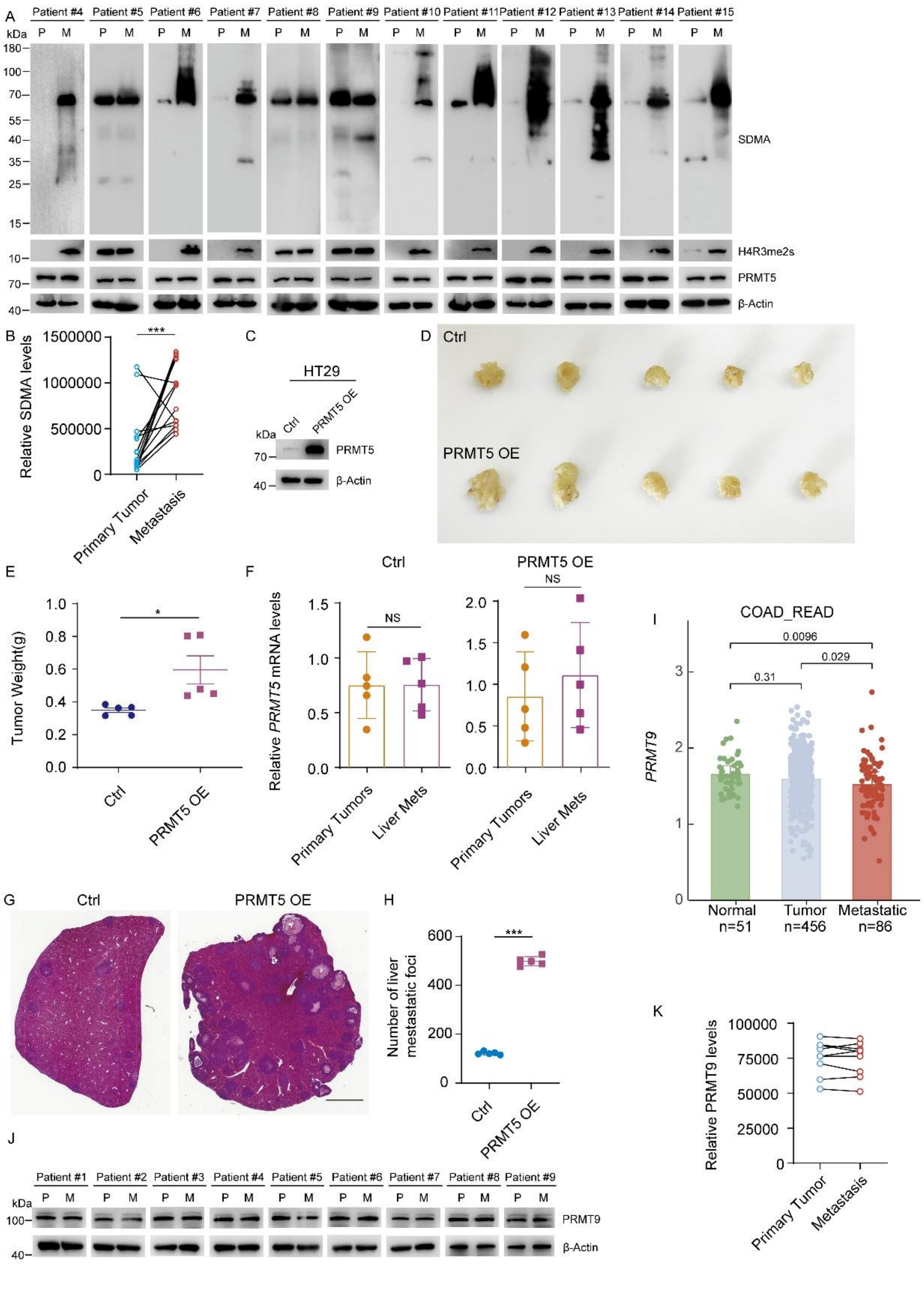

Supplemental Figure 1. PRMT5 promotes CRC growth and metastasis. (**A**) Western blot showing SDMA and PRMT5 expression levels in paired primary CRC (P) and liver metastatic samples (M). (**B**) Quantification of SDMA expression levels in paired primary CRC and liver metastatic samples. (**C**)Western blot showing the expression levels of PRMT5 in HT29 cells stably expressing empty vector or PRMT5 expression plasmids. (**D** and **E**) Representative images (D) and weight (E) of cecum tumors in NCG mice with cecum injection of HT29 cells. Error bars denote

mean ± SEM of five mice per group. *P* value was calculated based on two-tailed paired Student's t test (**P* <0.05). (**F**) RT-qPCR showing the expression levels of PRMT5 in paired primary tumor and liver metastases (Liver Mets) of NCG mice with cecum injection of HT29 cells. Error bars denote mean ± SEM of five mice per group. *P* value was calculated based on two-tailed paired Student's t test (NS, non-significant; **P* <0.05). (**G** and **H**) Representative H&E staining (G) and quantification (H) of the metastases in NCG mice with spleen injection of HT29 cells. Error bars denote mean ± SEM of five mice per group. Scale bar, 100μm. *P* value was calculated based on Student's t test (****P* <0.001). (**I**) TCGA database showing the expression levels of *PRMT9* in CRC. (**J**) Western blot showing PRMT9 expression levels in paired primary CRC (P) and liver metastatic samples (M). (**K**) Quantification of PRMT9 expression levels in paired primary CRC and liver metastatic samples.

A

Gene Name	Coverage [%]	# PSMs	Score Sequest HT
ADAR	43	95	176.98
U2SURP	37	92	169.68
HNRNPA1	70	95	194.17
PRMT5	64	160	239.42
CHD5	10	26	57.12
C6orf223	44	21	48.19
KIF4B	13	25	40.29
EWSR1	26	22	45.93
XRCC1	24	22	33.07
EIF2B4	18	13	29.64

B

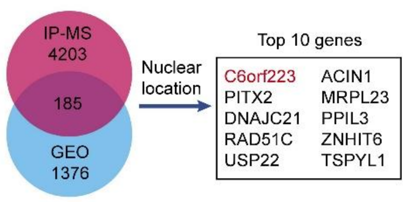

C

Uncharacterized protein c6orf223 OS=Homo sapiens OX=9606 GN=*C6orf223*

Coverage: 43.80%

Found Modifications: Carbamidomethyl(C)

	1	11	21	31
1	MMPLAEAGAL	AQGGGPSATE	WACILRRKTP	RHKQPTLLMV
41	RASRRSGKTS	AVLKAGRQSV	SGRKNSTSKD	LVTLGASSLR
81	EERGHPLHPR	HRKAVHLRTR	GRTRGWVQTL	ARMSRRTRGP
121	VERAAAAAAA	AAGGDAGHAP	FPPPPAADGA	RAPRSPGQVT
161	PRGLRLRLPR	RESLLRGLCR	PLRPLLGFRE	SDSAKPASLR
201	LLQHTPSARR	NYRIAGARLM	RSNYPPPLSS	AALRGAGPTR
241	RN			

D

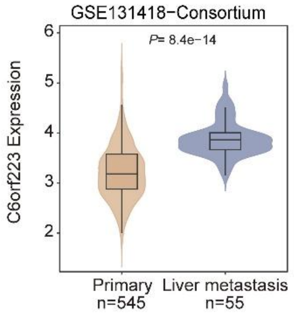

E

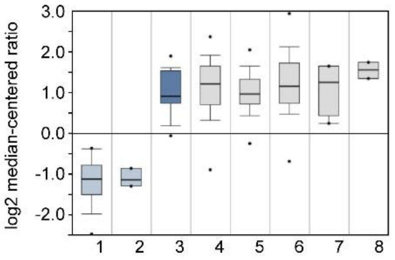

1. Colon(19)
2. Rectum(3)
3. Cecum Adenocarcinoma(22)
4. Colon Adenocarcinoma(101)
5. Colon Mucinous Adenocarcinoma(22)
6. Rectal Adenocarcinoma(60)
7. Rectal Mucinous Adenocarcinoma(6)
8. Rectosigmoid Adenocarcinoma(3)

F

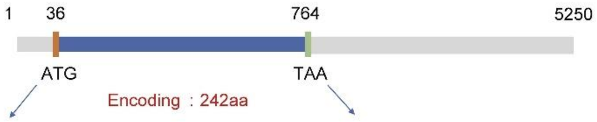

MMPLAEAGALAQGGGPSATEWACILRRKTPRH
KQPTLLMVRASRRSGKTSAVLKAGRQSVSGRK
NSTSKDLVTLGASSLREERGHPLHPRHRKAVHL
RTRGRTRGWVQTLARMSRRTRGPVERAAAAA
AAAAGGDAGHAPFPPPPAADGARAPRSPGQVT
PRGLRLRLPRRESLLRGLCRPLRPLLGFRESDS
AKPASLRLLQHTPSARRNYRIAGARLMRSNYPP
PLSSAALRGAGPTRRN

Supplemental Figure 2. C6orf223 potentially interacts with PRMT5. (**A**) List of the top 10 proteins interacted with PRMT5 identified by mass spectrometry. (**B**) TOP 10 genes after overlapping the results of co-IP-MS and the up-regulated genes in liver metastases. (**C**) Coverage peptides of C6orf223 (green shading) detected by mass spectrometry. (**D**) Expression levels of *C6orf223* between primary CRC and liver metastases in GEO dataset. (**E**) Analysis of the expression levels of C6orf223 in different types of adenocarcinomas in CRC patients and normal colon or

rectum using Oncomine. Error bars denote mean ± SEM of patients. (**F**) Schematic model of LINC03040 information encoding a small peptide with 242 amino acids.

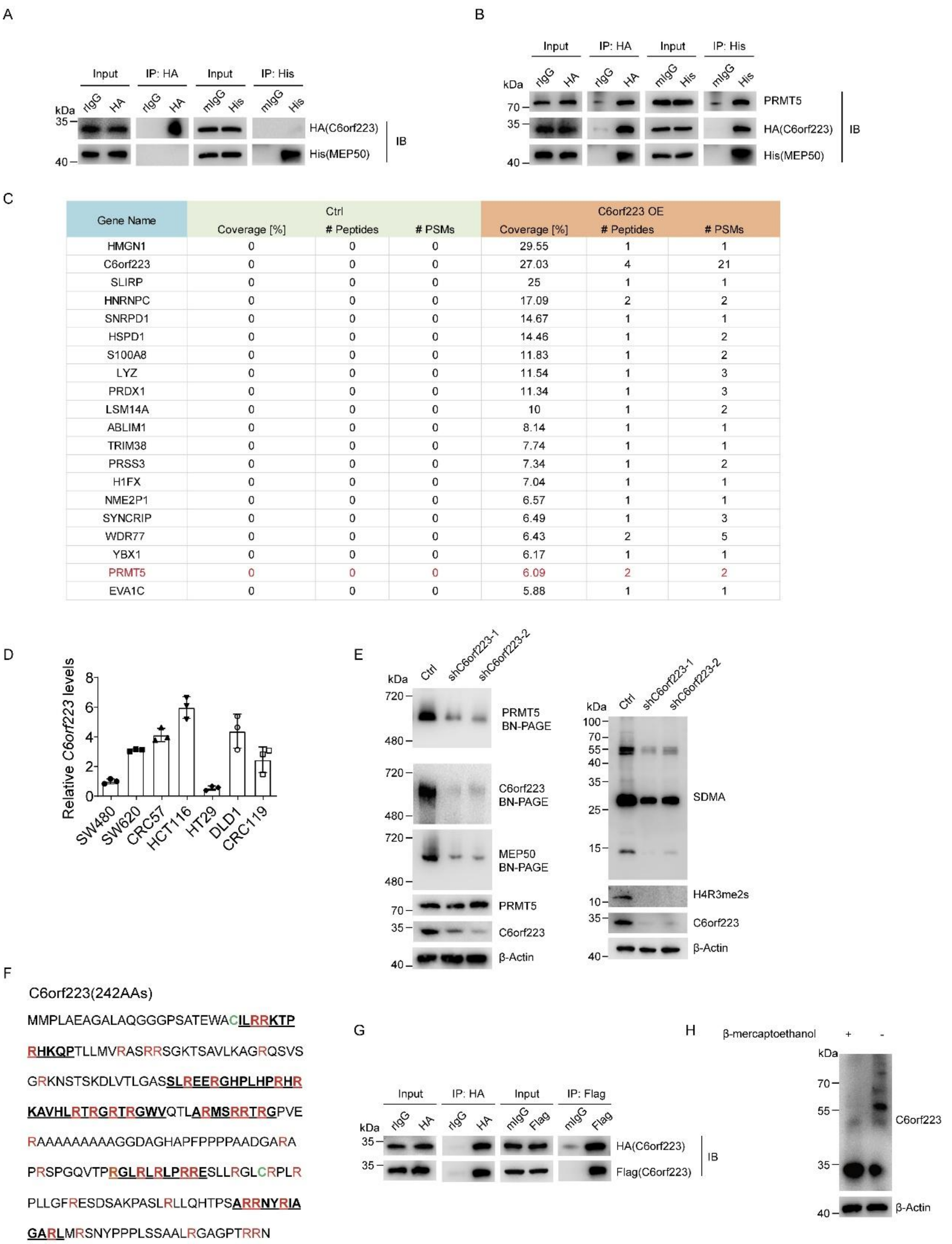

Gene Name	Ctrl Coverage [%]	Ctrl # Peptides	Ctrl # PSMs	C6orf223 OE Coverage [%]	C6orf223 OE # Peptides	C6orf223 OE # PSMs
HMGN1	0	0	0	29.55	1	1
C6orf223	0	0	0	27.03	4	21
SLIRP	0	0	0	25	1	1
HNRNPC	0	0	0	17.09	2	2
SNRPD1	0	0	0	14.67	1	1
HSPD1	0	0	0	14.46	1	2
S100A8	0	0	0	11.83	1	2
LYZ	0	0	0	11.54	1	3
PRDX1	0	0	0	11.34	1	3
LSM14A	0	0	0	10	1	2
ABLIM1	0	0	0	8.14	1	1
TRIM38	0	0	0	7.74	1	1
PRSS3	0	0	0	7.34	1	2
H1FX	0	0	0	7.04	1	1
NME2P1	0	0	0	6.57	1	1
SYNCRIP	0	0	0	6.49	1	3
WDR77	0	0	0	6.43	2	5
YBX1	0	0	0	6.17	1	1
PRMT5	0	0	0	6.09	2	2
EVA1C	0	0	0	5.88	1	1

Supplemental Figure 3. Validation of the interaction between C6orf223 and PRMT5. (**A**) Immunoprecipitation with anti-HA or anti-His antibody in HEK293T cells transfected with C6orf223-HA and MEP50-His, followed by Western blot for HA and His. (**B**) Immunoprecipitation with anti-HA or anti-His antibody in HEK293T cells transfected with C6orf223-HA, MEP50-His

and PRMT5, followed by Western blot for HA and His. (**C**) Top proteins interacted with C6orf223 identified by mass spectrometry. (**D**) RT-qPCR showing the expression levels of *C6orf223* in different CRC cell lines (SW480, SW620, HCT116, HT29 and DLD1) and two patient-derived xenograft (PDX) cell lines (CRC57 and CRC119). Error bars denote mean ± SD. (**E**) Blue native-PAGE showing the levels of PRMT5/MEP50 hetero-octamer and SDMA in HCT116 cells with empty vector (Ctrl) or C6orf223 knockdown (shC6orf223-1 and shC6orf223-2). (**F**) Amino acids sequence of C6orf223 with arginine enriched regions (bold and underlined) where arginine is marked in red. (**G**) Immunoblot analysis of HA-immunoprecipitates and Flag-immunoprecipitates from HEK293T cells expressing HA-tagged C6orf223 and His-tagged C6orf223. (**H**) Western blot showing β-mercaptoethanol destroys C6orf223 dimerization in HEK293T cells with His-tagged C6orf223.

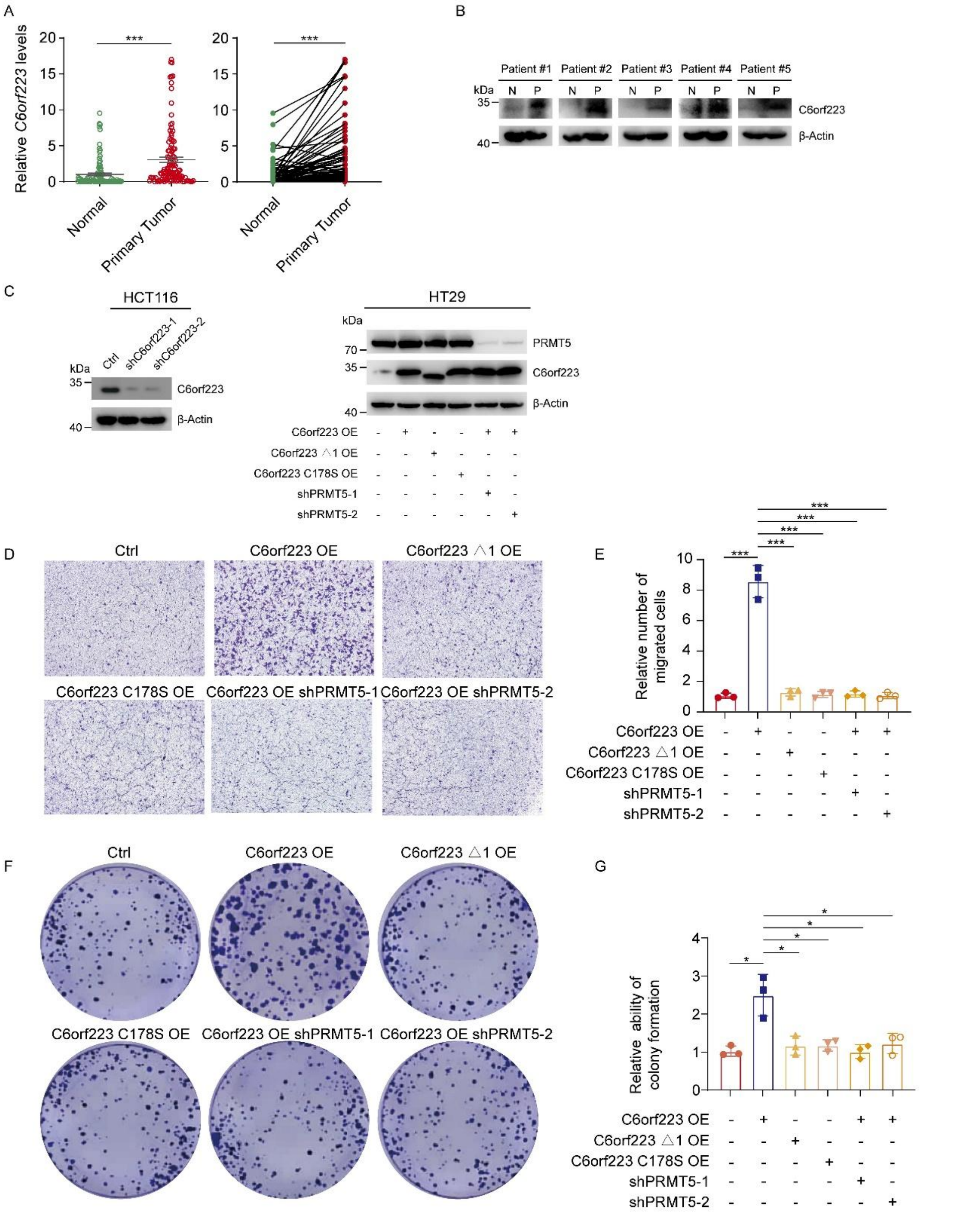

Supplemental Figure 4. C6orf223 promotes the migration and proliferation of CRC cells. (**A**) RT-qPCR showing the expression levels of C6orf223 in paired (right) or unpaired (left) pericarcinomatous tissue (Normal) and primary tumor of CRC patients. Error bars denote mean ± SEM. *P* value was calculated based on two-tailed paired Student's t test (****P* <0.01). (**B**) Western blot showing the expression levels of C6orf223 in paired pericarcinomatous tissue (N) and primary tumor(P) of CRC patients. (**C**) Western blot showing the expression levels of C6orf223 in HCT116

cells in which C6orf223 was knocked down, and the expression levels of C6orf223 and PRMT5 in HT29 cells transfected with full length or different C6orf223 mutations or both C6orf223 and PRMT5 knockdown. (**D** and **E**) Migration assay of HT29 cells with full length or different C6orf223 mutations or both C6orf223 and PRMT5 knockdown. Error bars denote mean ± SD of three independent repeated experiments. *P* value was calculated based on two-way ANOVA (****P* <0.001). (**F** and **G**) Colony formation of HT29 cells with full length or different C6orf223 mutations or both C6orf223 and PRMT5 knockdown. Error bars denote mean ± SD of three independent repeated experiments. *P* value was calculated based on two-way ANOVA (**P* <0.05).

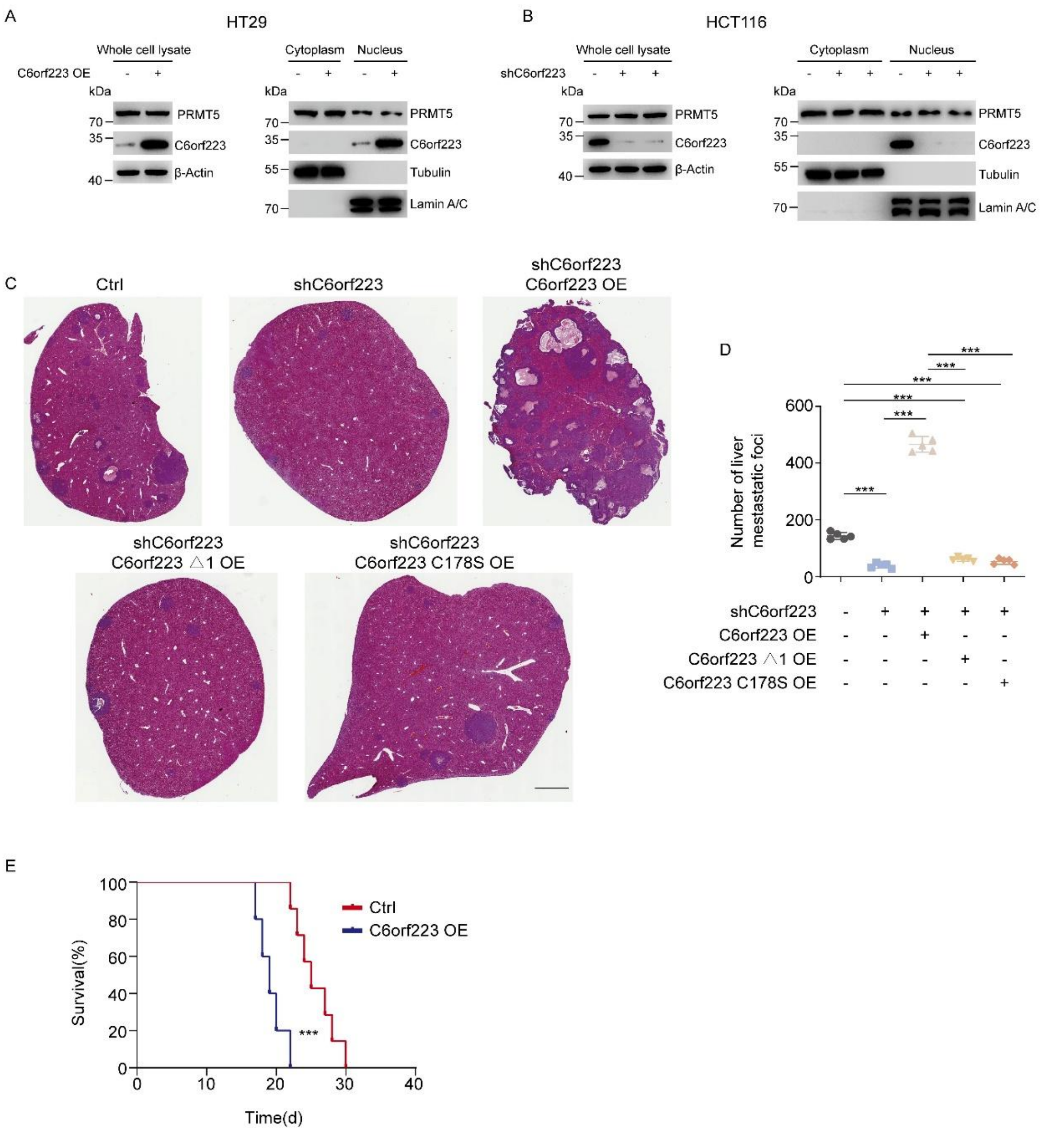

Supplemental Figure 5. C6orf223 promotes CRC metastasis. (**A** and **B**) Western blot analysis of PRMT5 expression in whole cell lysates, nucleus and cytoplasm fractions of HT29 (A) and HCT116

(B) cells. (**C** and **D**) Quantification (C) and representative H&E staining (D) and of the metastases in NCG mice with spleen injection of HCT116 cells. Error bars denote mean ± SEM of five mice per group. Scale bar, 100μm. *P* value was calculated based on two-way ANOVA (****P* <0.001). (**E**) Survival curve analysis of NCG mice with cecum injection of HT29 cells. *P* value was calculated based on log-rank test.

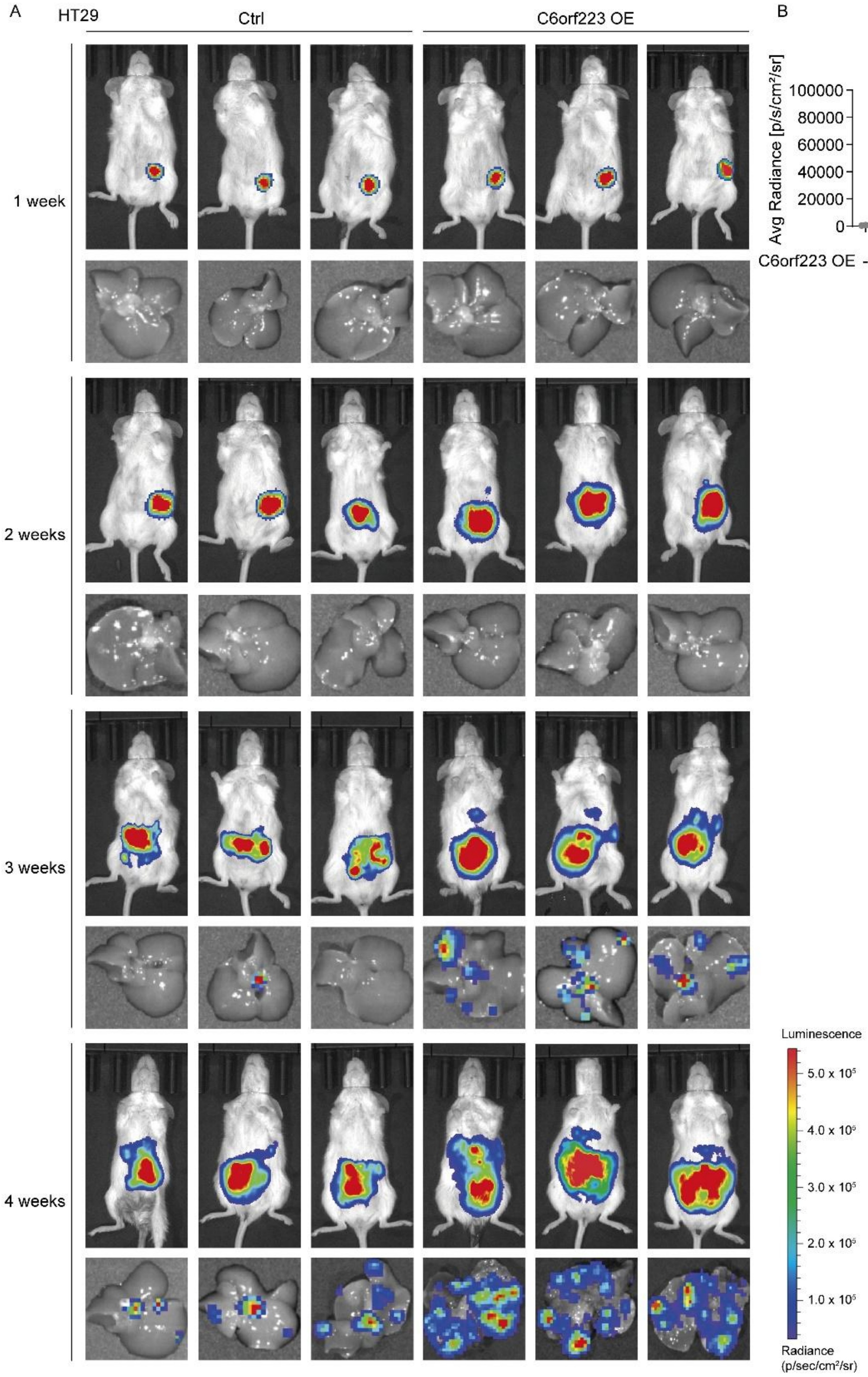

Supplemental Figure 6. The dynamic process of C6orf223-mediating CRC liver metastasis. (**A**) IVIS luciferase images of mice and liver. HT29 cells with ectopic expression of C6orf223 (C6orf223 OE) or not (Ctrl) were orthotopically inoculated in NCG mice. IVIS images of three mice in two groups was captured every week. (**B**) Quantification of the liver metastases in NCG mice with cecum injection of HT29 cells with ectopic expression of C6orf223 (C6orf223 OE) or not (Ctrl) through IVIS. Error bars denote mean ± SEM of three mice per group. *P* value was calculated based on two-way ANOVA (**P* <0.05, ****P* <0.001).

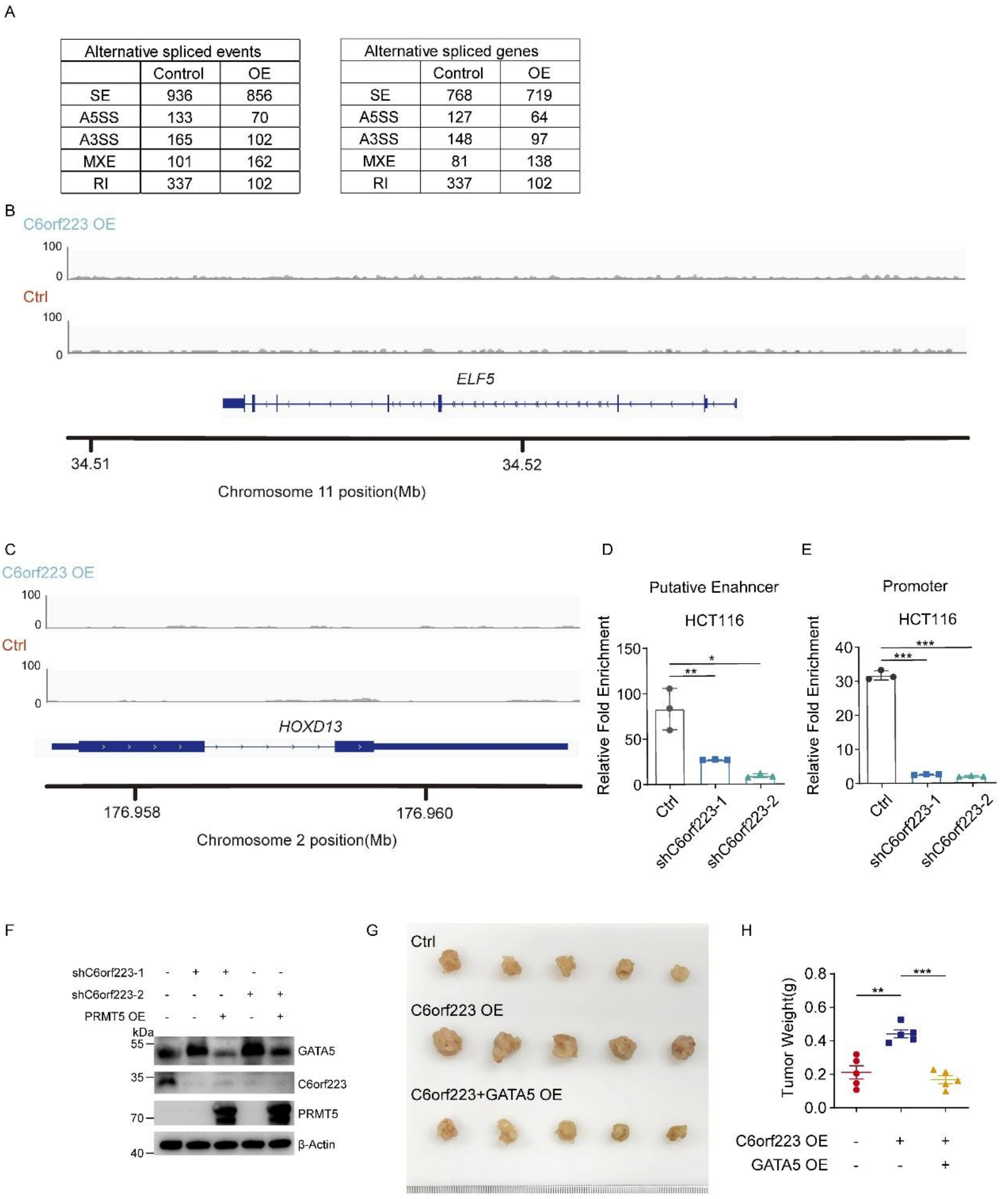

Supplemental Figure 7. C6orf223/PRMT5 axis promotes tumor growth via suppressing GATA5. (**A**) The alternative spliced events and number of genes in HT29 cells with C6orf223 overexpression or not. (**B** and **C**) Representative genome browser snapshots of H4R3me2s at the loci of of *ELF5* (B) and *HOXD13* (C) in HT29 cells with empty vector (Ctrl) or ectopic C6orf223 expression (C6orf223 OE). (**D** and **E**) RT-qPCR showing the enrichment of H4R3me2s in the putative enhancer (D) and promoter (E) of *GATA5* in HCT116 cells with empty vector (Ctrl) or

C6orf223 knockdown. Error bars denote mean ± SD of three independent repeated experiments. *P* value was calculated based on one-way ANOVA (**P* <0.05, ***P* <0.01, ****P* <0.001). (**F**) Western blot showing the expression level of GATA5 in HCT116 cells with C6orf223 knockdown or/and ectopic PRMT5 expression. (**G** and **H**) Representative images (G) and weight (H) of cecum tumors from NCG mice orthotopically injected with HT29 cells. Error bars denote mean ± SEM of five mice per group. *P* value was calculated based on one-way ANOVA (****P* <0.001, ***P* <0.01).

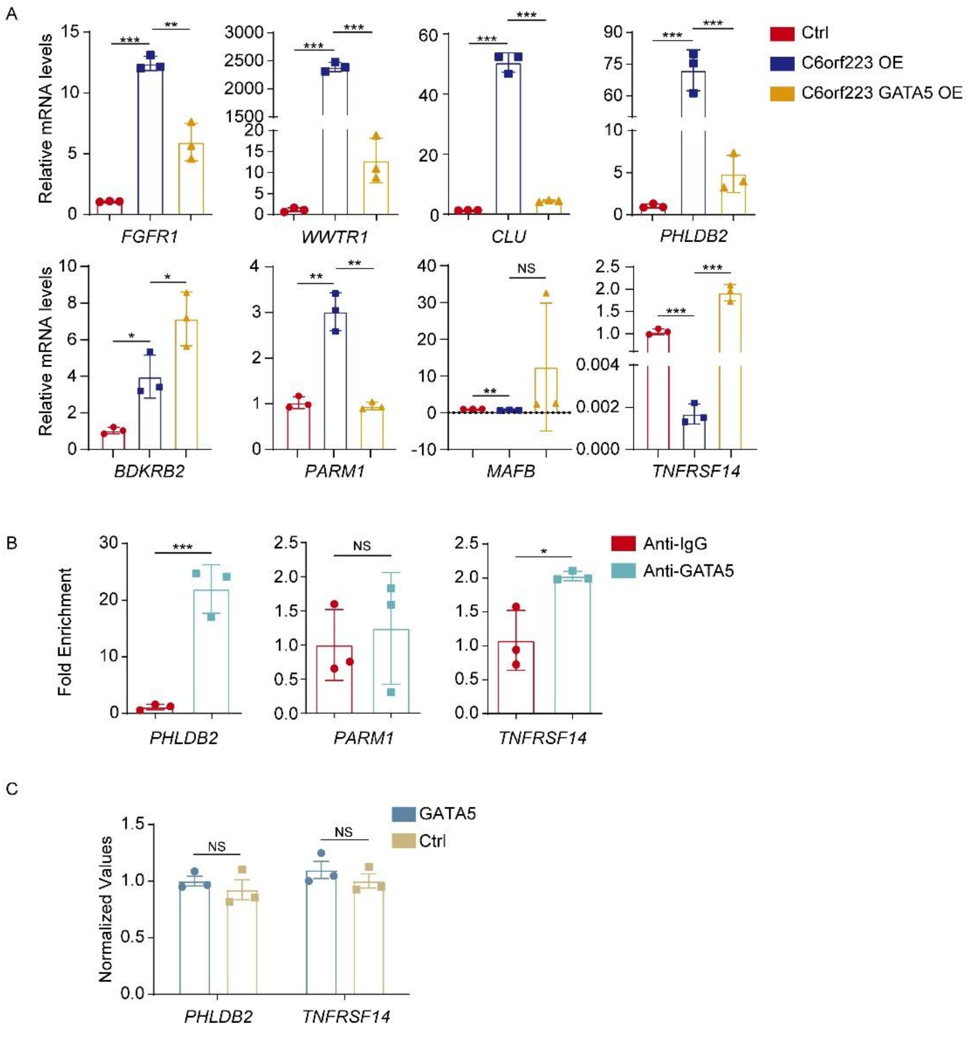

Supplemental Figure 8. Identification of GATA5 target genes. (**A**) RT-qPCR showing the expression levels of the putative GATA5 target genes in HT29 cells with ectopic expression of C6orf223 or C6orf223 together with GATA5. Error bars denote mean ± SD of three independent repeated experiments. *P* value was calculated based on one-way ANOVA (NS, no significant, **P* <0.05, ***P* <0.01, ****P* <0.001). (**B**) ChIP-qPCR showing the enrichment of GATA5 binding in the putative GATA5 target genes in HT29 cells with ectopic GATA5 expression. Error bars denote mean

± SD of three independent repeated experiments. *P* value was calculated based on based on two-way ANOVA (NS, no significant, **P* <0.05, ****P* <0.001). (**C**) Luciferase reporter assay of the putative GATA5 target genes. HEK293T cells were transfected with luciferase vector containing promoter sequence of the putative GATA5 target genes together with GATA5 plasmids or empty vector, followed by luciferase activity measurement. Error bars denote mean ± SD of three independent repeated experiments. *P* value was calculated based on two-way ANOVA (NS, no significant).

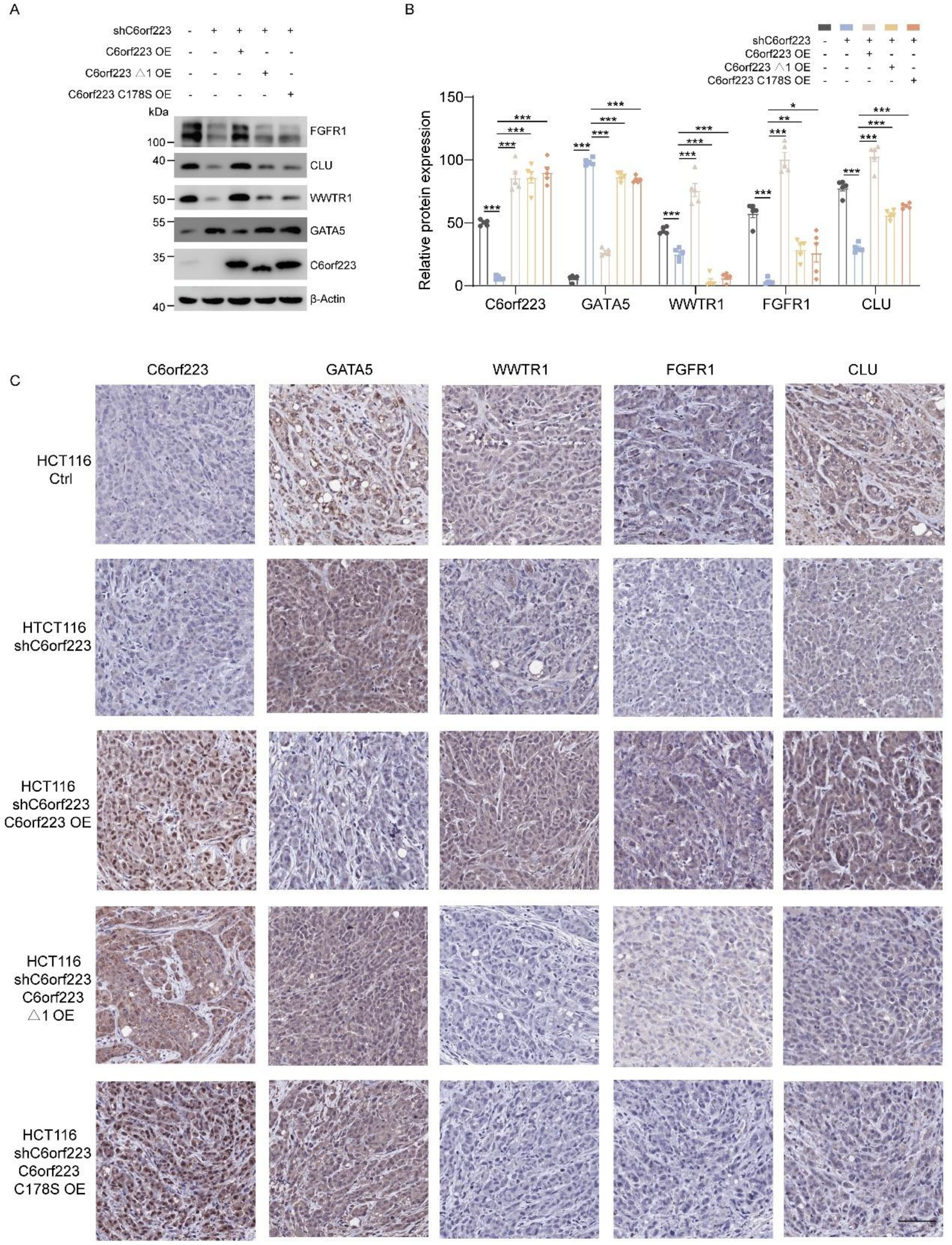

Supplemental Figure 9. Validation of downstream genes-mediated by C6orf223. (**A**) Western blot showing the expression levels of C6orf223, GATA5, FGFR1, CLU, WWTR1 in C6orf223-knocked down HCT116 cells rescued by full-length or different C6orf223 mutations. (**B** and **C**) Quantification (A) and representative immunohistochemistry image (B) showing the expression levels of C6orf223, GATA5, WWTR1, FGFR1 and CLU in primary cecum tumors from mice orthotopically injected HCT116 cells. Scale bar, 100μm. Error bars denote mean ± SEM of five

mice per group. *P* value was calculated based on two-way ANOVA (**P* <0.05, ***P* <0.01, ****P* <0.001).

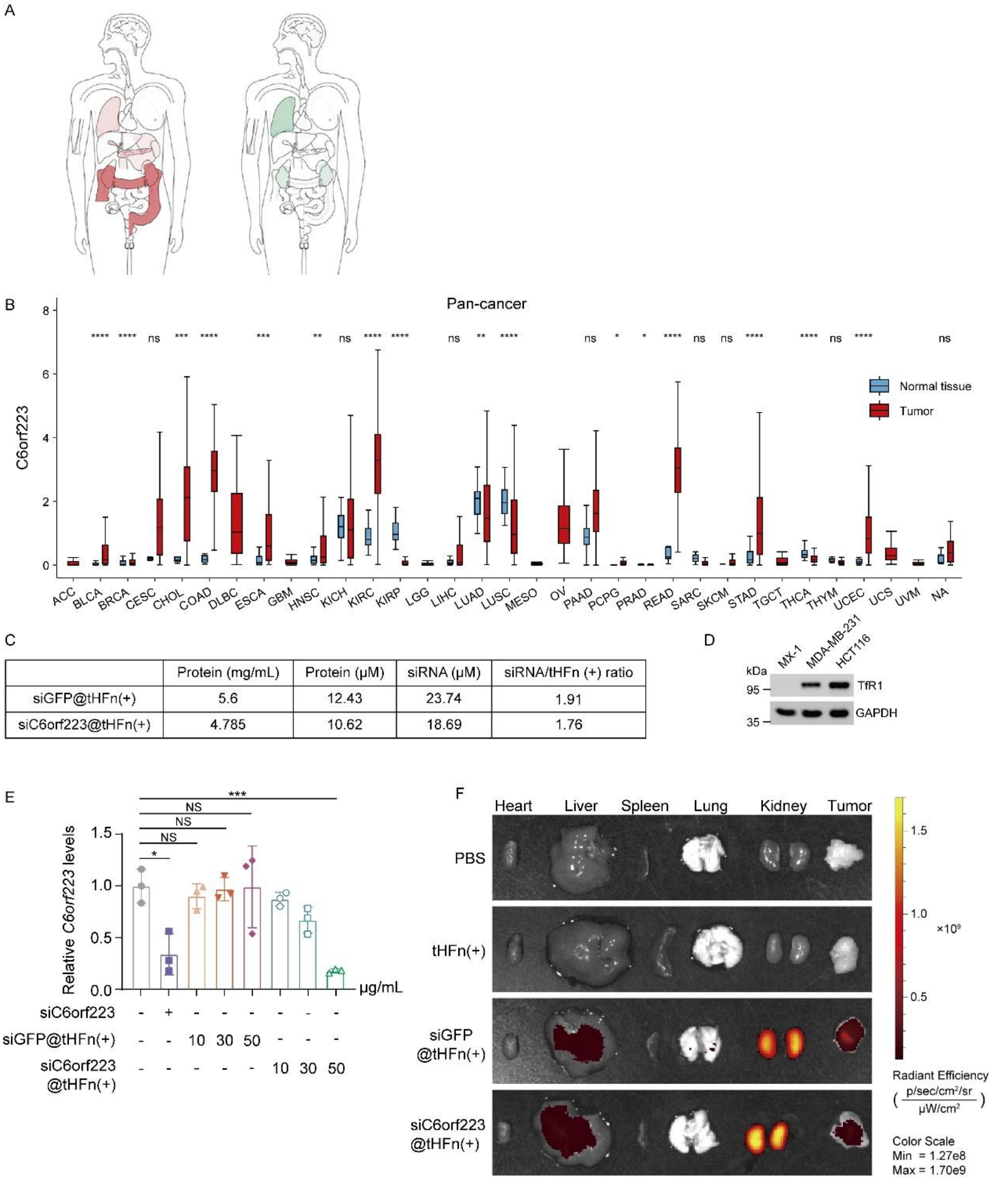

	Protein (mg/mL)	Protein (μM)	siRNA (μM)	siRNA/tHFn (+) ratio
siGFP@tHFn(+)	5.6	12.43	23.74	1.91
siC6orf223@tHFn(+)	4.785	10.62	18.69	1.76

Supplemental Figure 10. siC6orf223@tHFn(+) decreases C6orf223 expression *in vitro*. (**A**) The median expression of *C6orf223* in tumor (red) and normal samples (green) in body map. (**B**) GEPIA2 database showing the expression levels of *C6orf223* in cancer and normal tissues. (**C**) Protein and siRNA concentration in siGFP@tHFn(+) and siC6orf223@ tHFn(+). (**D**) Western blot showing the expression levels of TfR1 in cancer cells. (**E**) RT-qPCR showing the expression levels of C6orf223

in HCT116 cells after treatment with, siGFP@tHFn(+), siC6orf223@tHFn(+) or siC6orf223@Lipo2000. Error bars denote mean ± SD of three independent repeated experiments. *P* value was calculated based on two-way ANOVA (NS, no significant, **P* <0.05, ****P* <0.001). (**F**) *Ex vivo* fluorescent images of tumor and major organs of mice after the treatments of PBS, tHFn(+), siGFP@tHFn(+) and siC6orf223@tHFn(+).

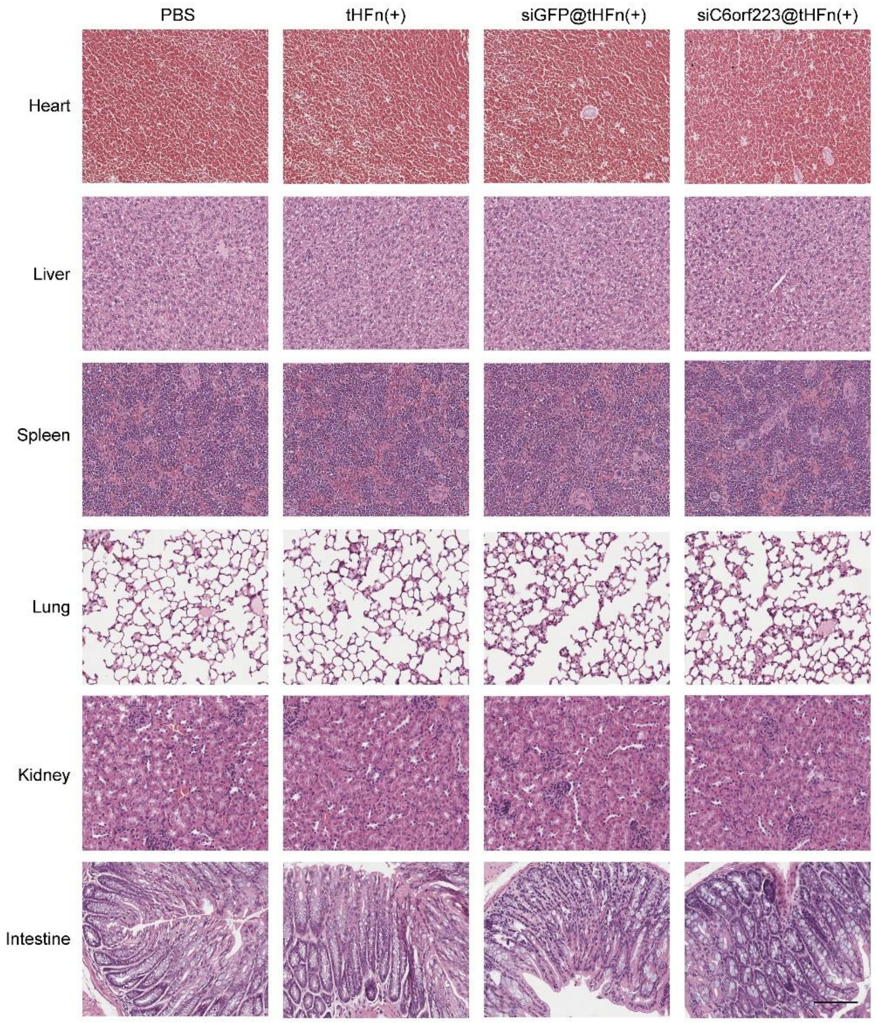

Supplemental Figure 11. siC6orf223@tHFn(+) treatment has no tissue toxicity. Representative H&E staining images of major organs after different treatments. Scale bar, 100μm.

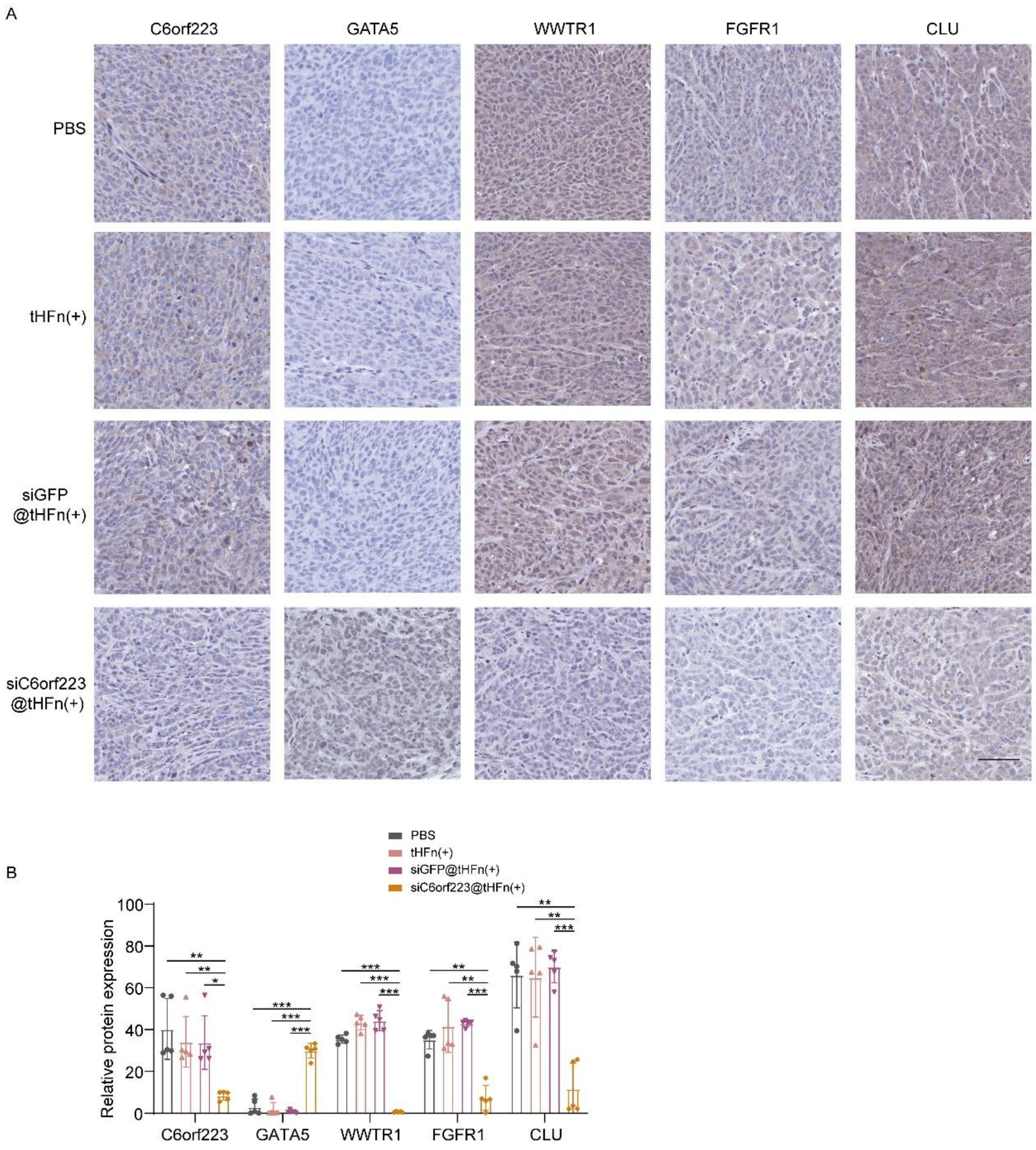

Supplemental Figure 12. Regulation of the expression of downstream targets by siC6orf223@tHFn(+) treatment. (**A** and **B**) Quantification (A) and representative immunohistochemistry image (B) showing the expression levels of C6orf223, GATA5, WWTR1, FGFR1 and CLU in primary tumors from mice orthotopically injected with HCT116 cells. The mice were treated with PBS, tHFn(+), siGFP@tHFn(+) or siC6orf223@tHFn(+). Error bars denote mean ± SEM of five mice per group. Scale bar, 100μm. *P* value was calculated based on two-way ANOVA (**P* <0.05, ***P* <0.01, ****P* <0.001).

Supplemental Table 1. shRNA and siRNA sequences

Human shC6orf233-1	CCGGATCGCTTCGCCTCCTACAACACTCGAGTGTTGTAG GAGGCGAAGCGATTTTTG
Human shC6orf233-2	CCGGCGAGCGCGAGAAGAAATTACACTCGAGTGTAATTT CTTCTCGCGCTCGTTTTTG
Human shPRMT5-1	CCGGGCCCAGTTTGAGATGCCTTATCTCGAGATAAGGCAT CTCAAACTGGGCTTTTTG
Human shPRMT5-2	CCGGGCCCAGTTTGAGATGCCTTATCTCGAGATAAGGCAT CTCAAACTGGGCTTTTTG
siGFP forward	5’CUU CAG CCU CAG CUU GCC GTT3’
siGFP reverse	5’CGG CAA GCU GAC CCU GAA GTT3’
siC6orf233 forward	5’CGAGCGCGAGAAGAAAUUACATT3’
siC6orf233 reverse	5’UGUAAUUUCUUCUCGCGCUCGTT3’

Supplemental Table 2. Clinical sample information

Patients from the 7th Medical Center of PLA General Hospital					
**Patients No.**	**Gender**	**Age**	**Primary site**	**TNM**	**Stage**
P1	Male	58	Colon	pT3N1M1	4
P2	Male	63	Colon	pT2N0M1	4
P3	Female	84	Colon	pT3N1M0	3
P4	Male	65	Colon	pT3N0M0	2
P5	Male	60	Sigmoid colon	pT3N0M0	2
P6	Female	40	Rectum	pT3N0M0	2
P7	Female	46	Rectum	pT3N1M0	3
P8	Male	58	Sigmoid colon	pT3N0M0	2
P9	Female	55	Rectum	pT3N0M0	2
P10	Female	71	Rectum	pT2N1M0	3
P11	Female	47	Sigmoid colon	pT4N2M0	3
P12	Female	42	Sigmoid colon	pT2N0M0	1
P13	Female	63	Rectum	pT3N0M0	2
P14	Male	58	Colon	pT3N1M0	3
P15	Female	77	Sigmoid colon	pT3N0M0	2
P16	Female	56	Rectum	pT3N2M0	3
P17	Male	62	Colon	pT3N1M0	3
P18	Male	72	Colon	pT3N1M0	3
P19	Male	69	Colon	pT3N1M0	3
P20	Male	68	Rectum	pT3N2M1	4
P21	Male	33	Colon	pT4N2M1	4
P22	Male	51	Rectum	pT4N3M0	3
P23	Male	41	Rectum	pT3N0M0	2
P24	Female	63	Rectum	pT1N0M0	1
P25	Male	46	Rectum	pT2N0M0	1
P26	Male	67	Rectum	pT2N1M0	3

P27	Male	60	Rectum	pT3N1M0	3
P28	Male	66	Rectum	pT3N0M0	2
P29	Male	63	Colon	pT0N0M1	4
P30	Male	54	Colon	pT3N0M0	2
P31	Female	65	Colon	pT3N2M0	3
P32	Male	68	Sigmoid colon	pT3N1M0	3
P33	Male	87	Sigmoid colon	pT3N0M0	2
P34	Female	44	Rectum	pT3N2M0	3
P35	Male	71	Colon	pT3N1M0	3
P36	Male	68	Sigmoid colon	pT2N0M0	1
P37	Male	60	Colon	pT3N0M0	2
P38	Female	33	Sigmoid colon	pT3N1M0	3
P39	Male	75	Colon	pT3N0M0	2
P40	Female	56	Rectum	pT3N2M0	3
P41	Female	62	Rectum	pT3N0M0	2
P42	Male	56	Sigmoid colon	pT3N1M0	3
P43	Male	74	Sigmoid colon	pT3N1M0	3
P44	Female	62	Rectum	pT3N2M0	3
P45	Female	66	Colon	pT3N1M0	3
P46	Male	65	Rectum	pT3N0M0	2
P47	Male	72	Sigmoid colon	pT3N0M0	2
P48	Female	70	Colon	pT3N1M0	3
P49	Female	39	Rectum	pT3N2M0	3
P50	Female	64	Colon	pT3N0M0	2
P51	Female	78	Colon	pT4N0M0	2
P52	Male	61	Rectum	pT3N1M0	3
P53	Male	65	Sigmoid colon	pT3N0M0	2
P54	Male	65	Rectum	pT3N0M0	2
P55	Female	54	Colon	pT2N0M0	1

P56	Female	70	Colon	pT3N0M0	2
P57	Male	59	Rectum	pT3N1M0	3
P58	Female	81	Colon	pT3N2M0	3
P59	Male	62	Colon	pT3N0M0	2
P60	Female	59	Rectum	pT3N0M0	2
P61	Male	63	Rectum	pT3N0M0	2
P62	Male	53	Rectum	pT2N0M0	1
P63	Male	63	Colon	pT3N2M0	3
P64	Male	63	Rectum	pT3N0M0	2
P65	Female	70	Sigmoid colon	pT3N0M0	2
P66	Male	55	Sigmoid colon	pT3N2M0	3
P67	Female	46	Colon	pT3N0M0	2
P68	Male	73	Rectum	pT3N2M0	3
P69	Male	59	Rectum	pT3N2M0	3
P70	Female	68	Rectum	pT2N0M0	1
P71	Male	37	Colon	pT3N1M0	3
P72	Male	62	Rectum	pT3N1M0	3
P73	Male	93	Colon	pT3N1M0	3
P74	Male	61	Rectum	pT3N2M0	3
P75	Male	60	Colon	pT2N0M0	1
P76	Male	53	Rectum	pT3N2M0	3
P77	Female	68	Colon	pT3N0M0	2
P78	Female	57	Colon	pT3N0M0	2
P79	Male	69	Colon	pT3N0M0	2
P80	Female	52	Sigmoid colon	pT3N2M0	3
P81	Male	55	Sigmoid colon	pT3N1M0	3
P82	Male	80	Sigmoid colon	pT3N0M0	2
P83	Male	51	Sigmoid colon	pT3N1M0	3
P84	Female	72	Colon	pT4N2M0	3

P85	Male	63	Sigmoid colon	pT3N1M0	3
P86	Male	62	Colon	pT3N0M0	2
P87	Male	69	Colon	pT3N1M0	3
P88	Female	82	Colon	pT3N1M0	3
P89	Female	81	Rectum	pT4N0M0	2
P90	Female	52	Rectum	pT3N1M0	3
P91	Female	68	Rectum	pT4N0M0	2
P92	Male	72	Colon	pT3N0M0	2
P93	Male	55	Sigmoid colon	pT4N1M0	3
P94	Male	36	Sigmoid colon	pT2N1M0	3
P95	Female	49	Rectum	pT2N0M0	1
P96	Female	39	Colon	pT3N0M0	2
P97	Female	58	Colon	pT3N0M0	2
P98	Female	70	Sigmoid colon	pT3N0M0	2
P99	Female	79	Rectum	pT3N1M0	3
P100	Male	60	Sigmoid colon	pT3N1M0	3
P101	Male	52	Sigmoid colon	pT4N0M0	2
P102	Male	76	Rectum	pT3N2M0	3
P103	Male	68	Rectum	pT4N0M0	2
P104	Female	58	Rectum	pT3N2M0	3
P105	Male	70	Rectum	pT3N0M0	2
P106	Female	38	Rectum	pT3N0M0	2
P107	Female	85	Colon	pT3N0M0	2
P108	Male	64	Sigmoid colon	pT3N0M0	2
P109	Male	72	Rectum	pT3N1M0	3
P110	Male	48	Rectum	pT3N2M0	3
P111	Female	58	Rectum	pT4N2M0	3
P112	Male	63	Rectum	pT2N0M0	1
P113	Male	77	Rectum	pT2N0M0	1

P114	Male	64	Rectum	pT3N0M0	2
P115	Female	61	Rectum	pT3N0M0	2
P116	Male	54	Rectum	pT4N1M0	3
P117	Male	29	Colon	pT3N0M0	2
P118	Male	53	Colon	pT3N2M0	3
P119	Female	77	Colon	pT3N0M0	2
P120	Female	60	Colon	pT3N0M0	2
P121	Male	55	Colon	pT3N1M0	3
P122	Female	63	Sigmoid colon	pT3N0M1	2
P123	Female	53	Colon	pT4N2aM1	2
P124	Male	61	Sigmoid colon	pT3N0M1	2
P125	Female	66	Colon	pT3N1bM1	2
P126	Male	60	Sigmoid colon	pT3N0M1	2
P127	Male	73	Sigmoid colon	pT3N1aM1	2
P128	Male	61	Colon	pT3N0M1	2
P129	Male	47	Colon	pT3N0M1	2
P130	Male	50	Sigmoid colon	pT3N0M1	2
P131	Male	64	Colon	pT2aN0M1	2
P132	Male	71	Sigmoid colon	pT3N0M1	2
P133	Female	65	Sigmoid colon	pT3N0M1	2
P134	Female	50	Colon	pT3N0M1	2
P135	Female	55	Colon	pT3N0M1	3
P136	Male	73	Sigmoid colon	pT3N0M1	1
P137	Male	47	Colon	pT3N0M1	3
P138	Male	63	Colon	pT3N0M1	2
P139	Male	46	Rectum	pT3N1bM1	2
P140	Male	67	Rectum	pT3N2aM1	2
P141	Male	72	Rectum	pT3N0M1	2
P142	Male	59	Rectum	pT3N1bM1	3

P143	Male	59	Rectum	pT3N0M1	3
P144	Male	50	Rectum	pT3N1M1	2
P145	Male	63	Rectum	pT3N1aM1	2
P146	Female	53	Rectum	pT3N2aM1	2
P147	Male	62	Rectum	pT3N1bM1	2
P148	Female	66	Rectum	pT3N0M1	2
P149	Male	60	Rectum	pT3N0M1	2

Supplemental Table 3. RT-qPCR primer sequences

Gene symbol	Primer forward	Primer reverse
PRMT5	GCTTCTGGGCTCATTTGCTG	CAGCCGTACCACATAAGGCA
C6orf223	CTTCAGCAACGGAGTGGG	GACTGCTTTCCTATGTCTGGG
GATA5	GCTCGTTCGGCCTCAGAAG	ATGGTCTTTGGCTTCCGCTT
CLU	GTCGCTGAGAGGTTGACCAG	GTCCGAGTCAGAAGTGTGGG
PHLDB2	CTTCGTTGCTCGAACTCCCTG	TGCTCTTCCATAATCTTGCTGG
BDKRB2	TGCTAGTCCTGGTTGTGCTG	TCACGTACACCAGTGGGTTG
PARM1	CTGTCAGTCCGCAAACTCCT	TAGTCCAGATGGTGGTCGGT
TNFRSF14	AGCCTCGTCATCGTCATTGT	GGGTATTGTCTCCTCCACGG
FGFR1	TTTGAGACCGCACAGGAGTG	TAGCGCAGTCTTTGGGGAAA
WWTR1	CATGGCAGTATCCCAGCCAA	CTGGATTCTCTGAAGCCGCA
MAFB	GGTGAGAAGGGATCGCAGTT	TGATGCAAAATGCCCGGAAC

Supplemental Table 4. ChIP-qPCR primer sequences

Gene symbol	Primer forward	Primer reverse
GATA5	GCCGAGGCATTCCTTGTG	GACCGTGAGGAGTCTCCAAACC
WWTR1	ATCAATCCTTAATTTCCTCTACGGG	AATCCTCCCACAACCCTCAC
TNFRSF14	TGACAAGCTGTGCCTCAGAA	CTTTGCCTGGACAGCTCCT
CLU	AACATCACACTGATGGGGGTC	CACCCTGCTTGGTTGGGTTC
FGFR1	CACTACCTTTCATAGTGACTTCCA	CAGCACTTGGTCTGAAGGATTC
PHLDB2	AGATTTCCCTCTGAGATTGCTGA	AGAAAGCACAGTCTGATAGCCA
PARM1	TCACTCAGGAAACGTCAGCC	GTGAGCACATCTCTGCCCTT
