# Supplementary material for: C6orf223 promotes colorectal cancer growth and metastasis by facilitating PRMT5-MEP50 multiprotein complex assembling
Source: J Clin Invest. 2025 Oct 15;135(20):e186052. doi: 10.1172/JCI186052 (PMC12520688; doi:10.1172/JCI186052)

Full unedited blot/gel for Figure 1D

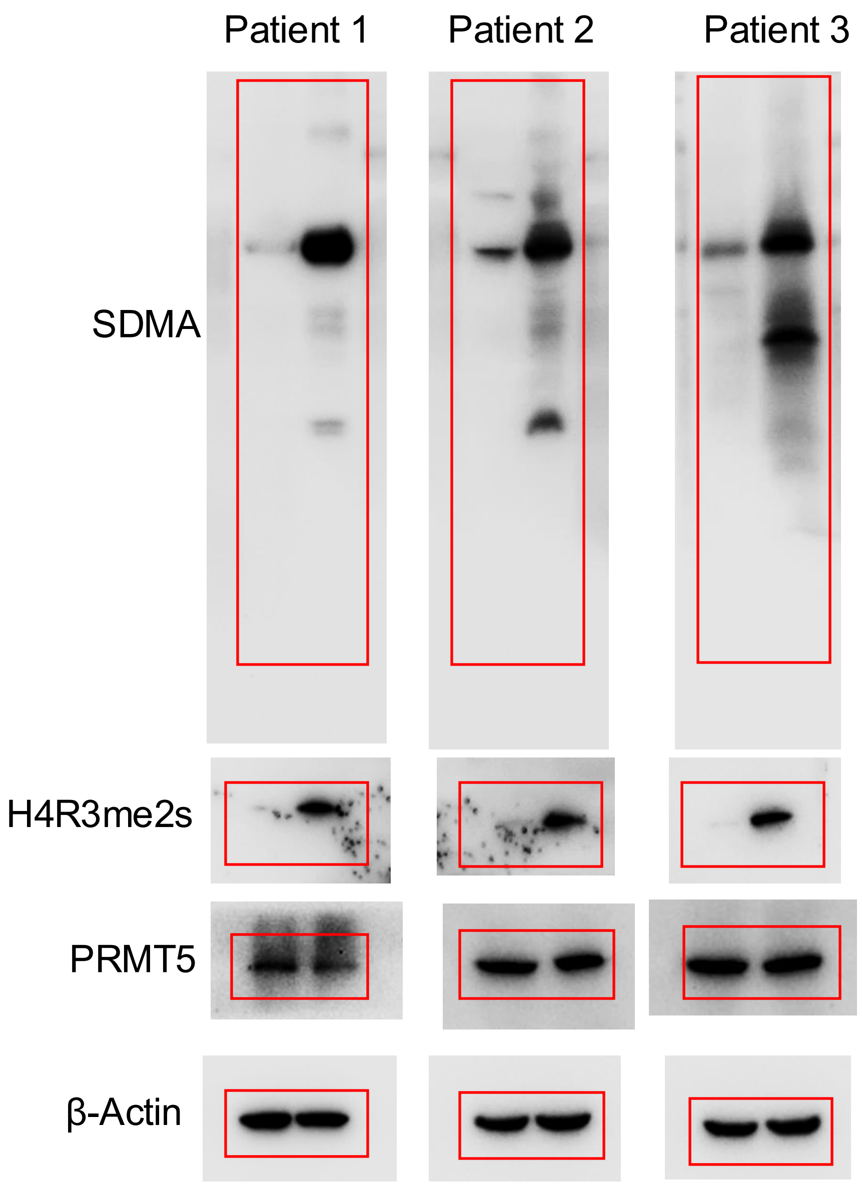

Full unedited blot/gel for Figure 1G

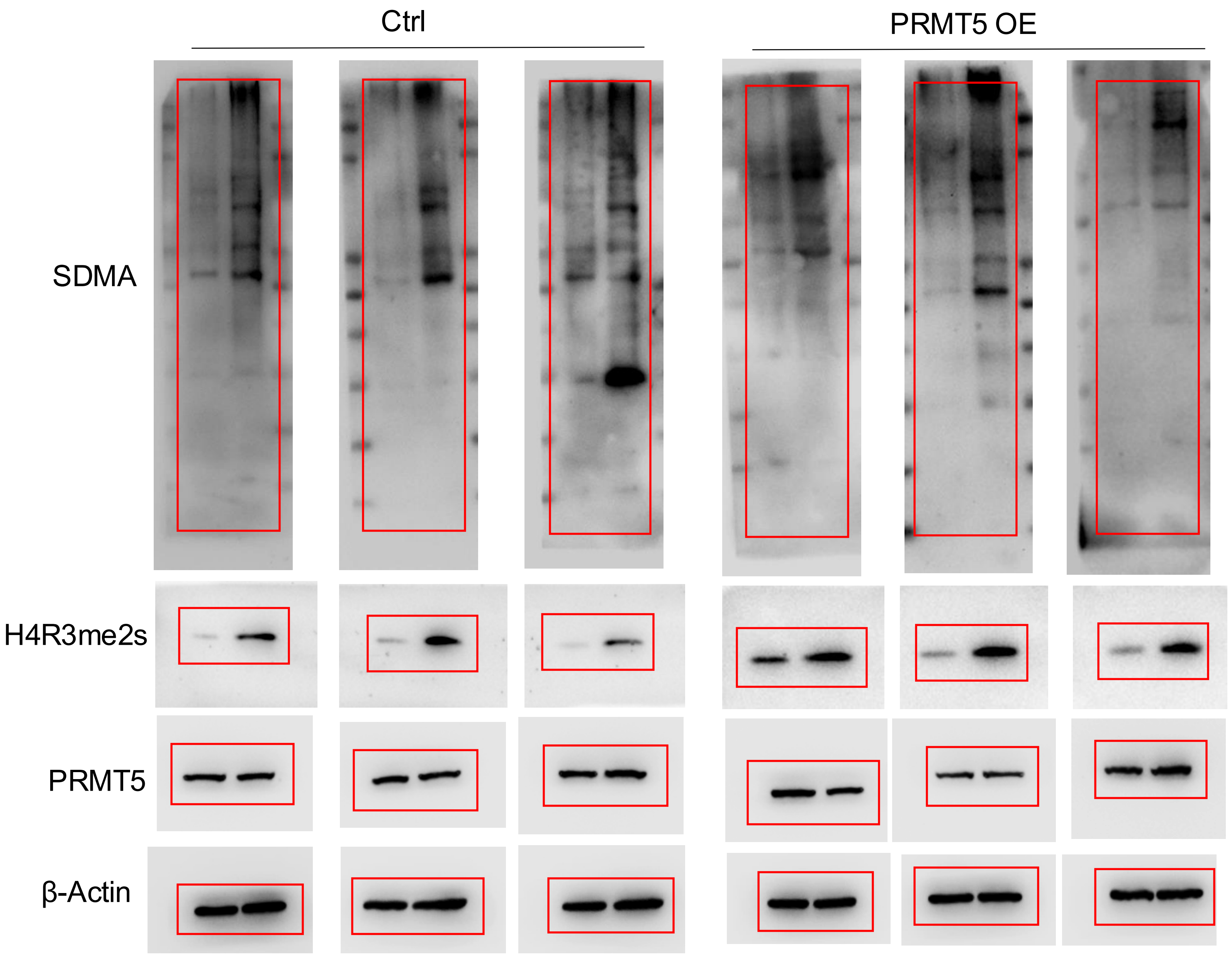

Full unedited blot/gel for Figure 1I

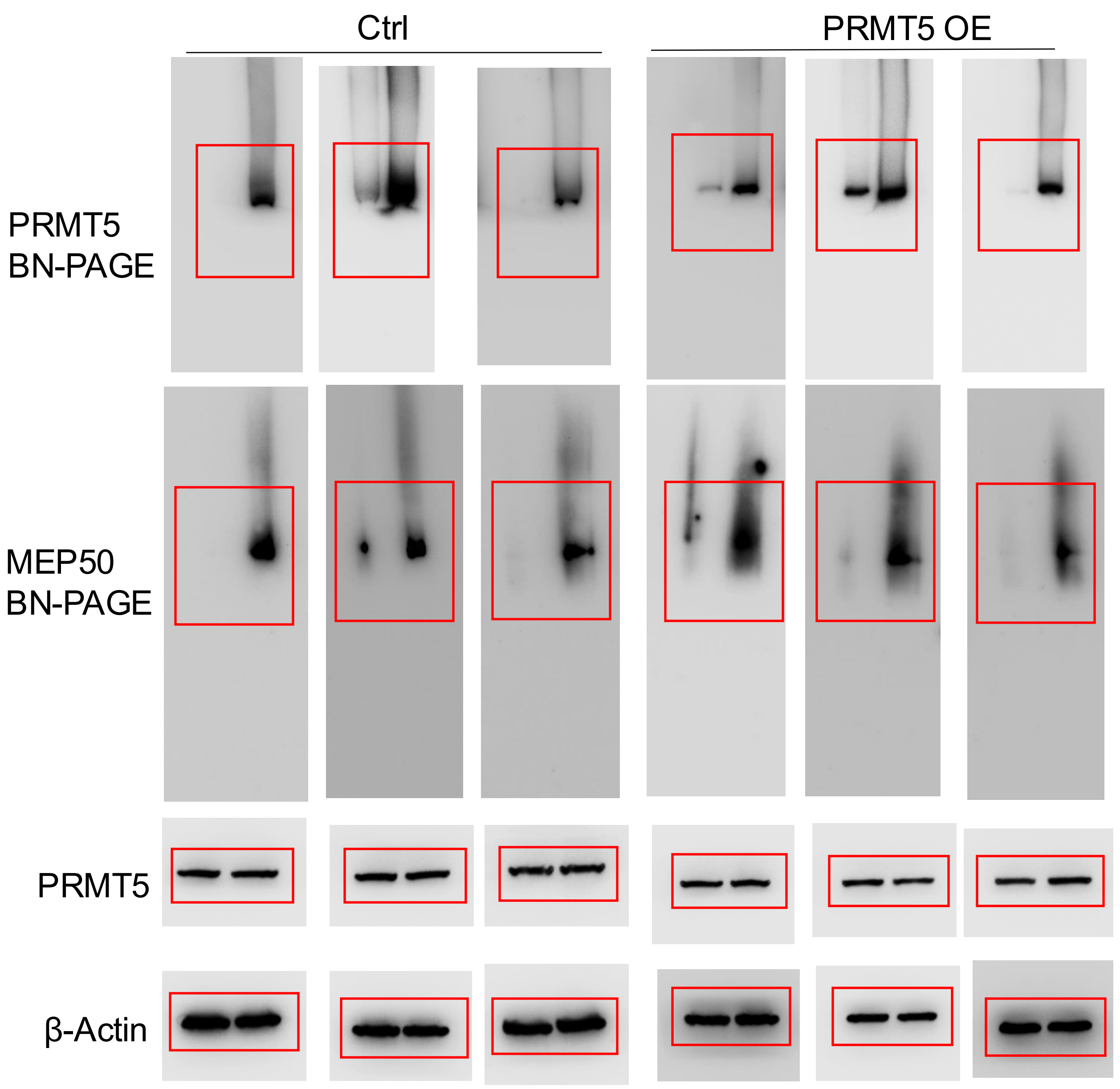

Full unedited blot/gel for Figure 1J

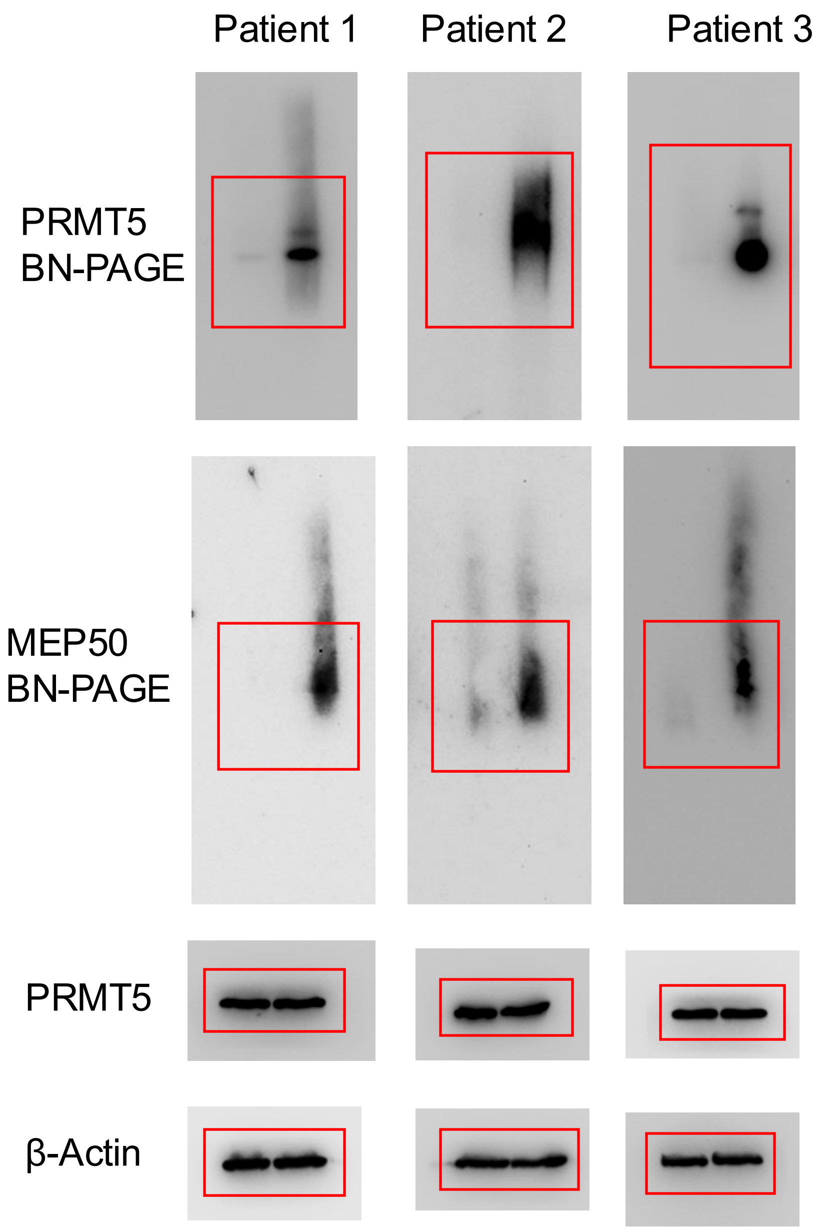

Full unedited blot/gel for Figure 2A

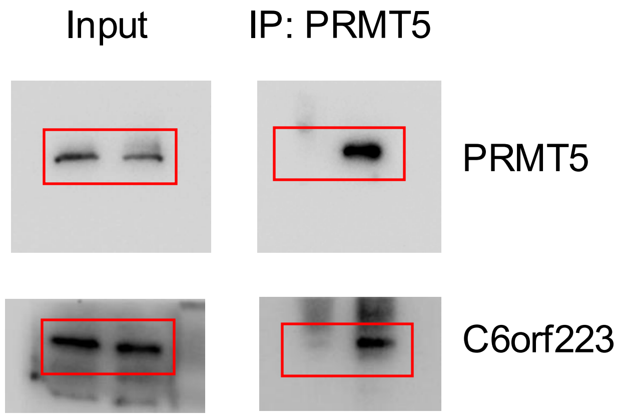

Full unedited blot/gel for Figure 2B

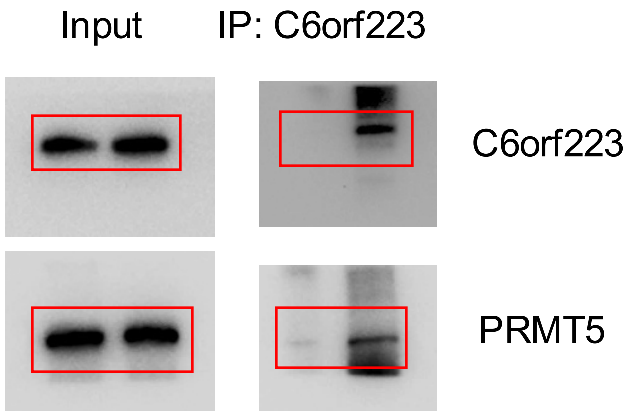

Full unedited blot/gel for Figure 2F

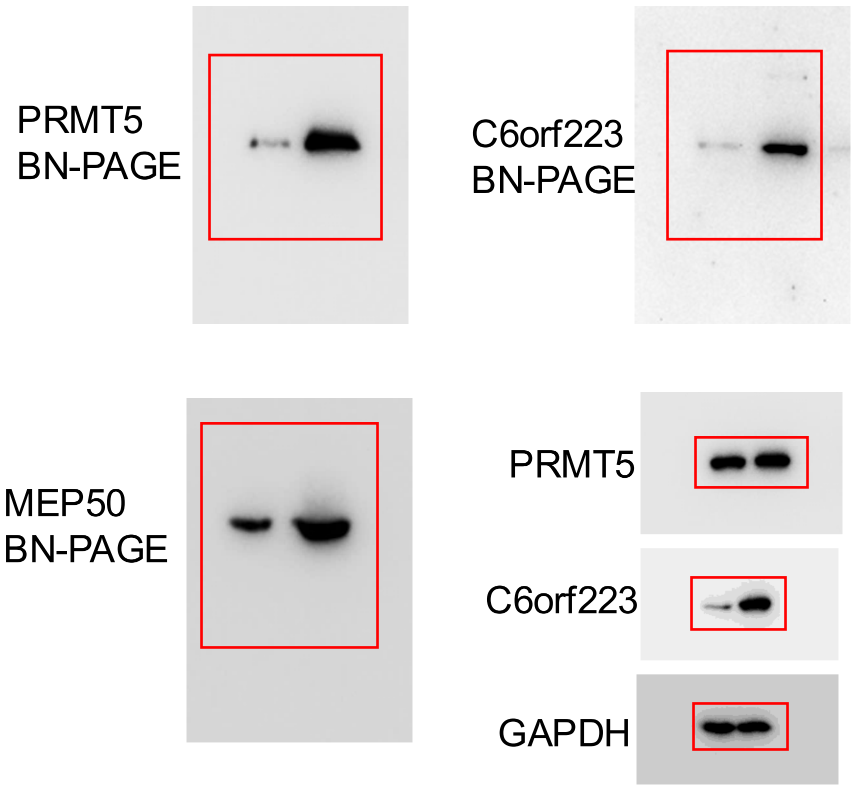

Full unedited blot/gel for Figure 2C

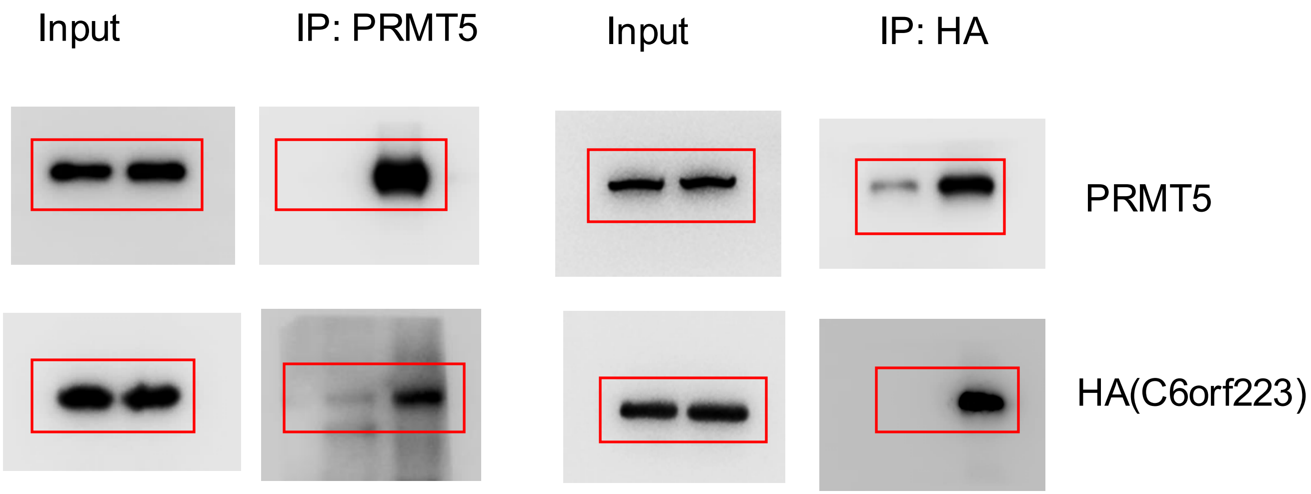

Full unedited blot/gel for Figure 2H

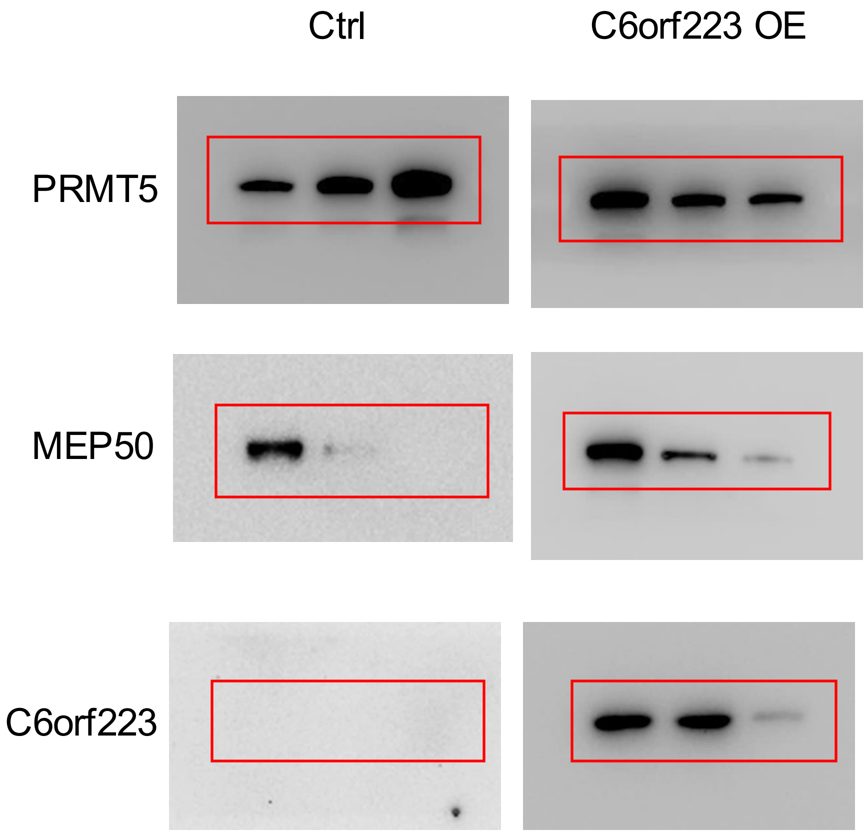

Full unedited blot/gel for Figure 2I

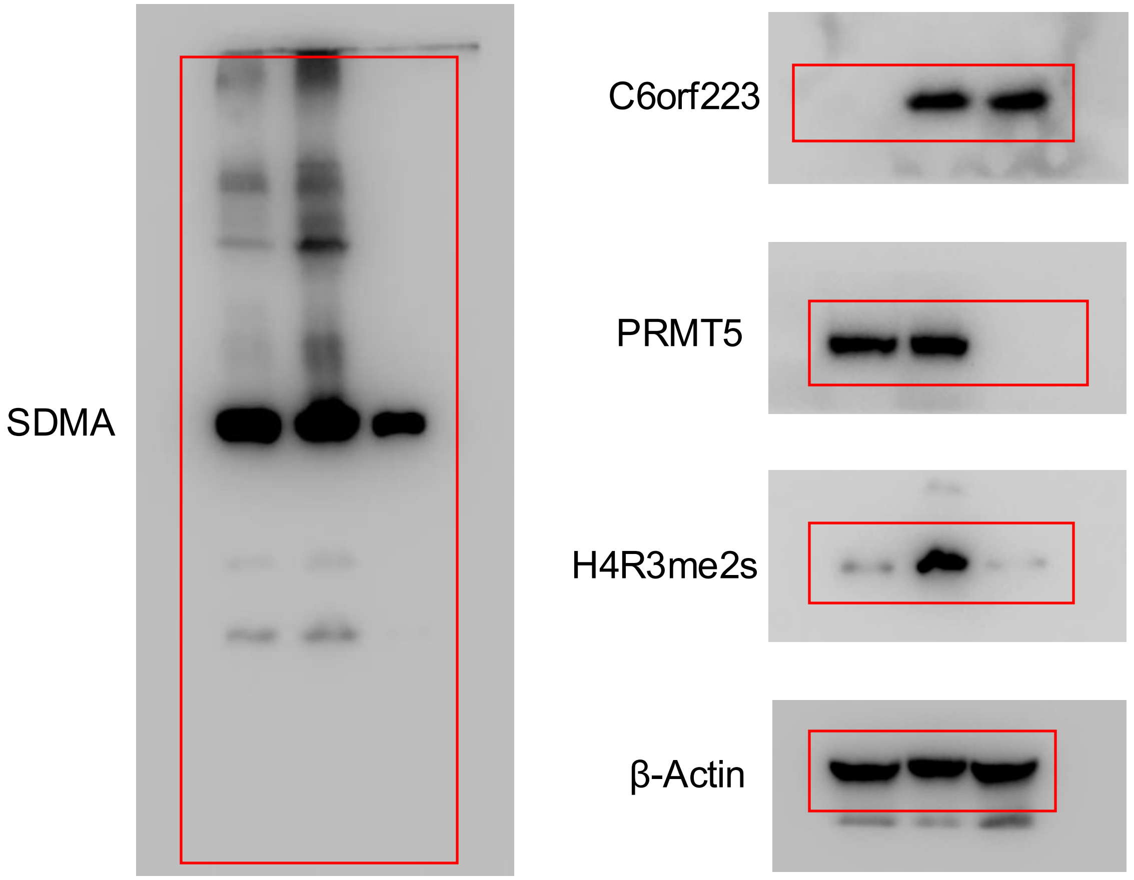

Full unedited blot/gel for Figure 3B

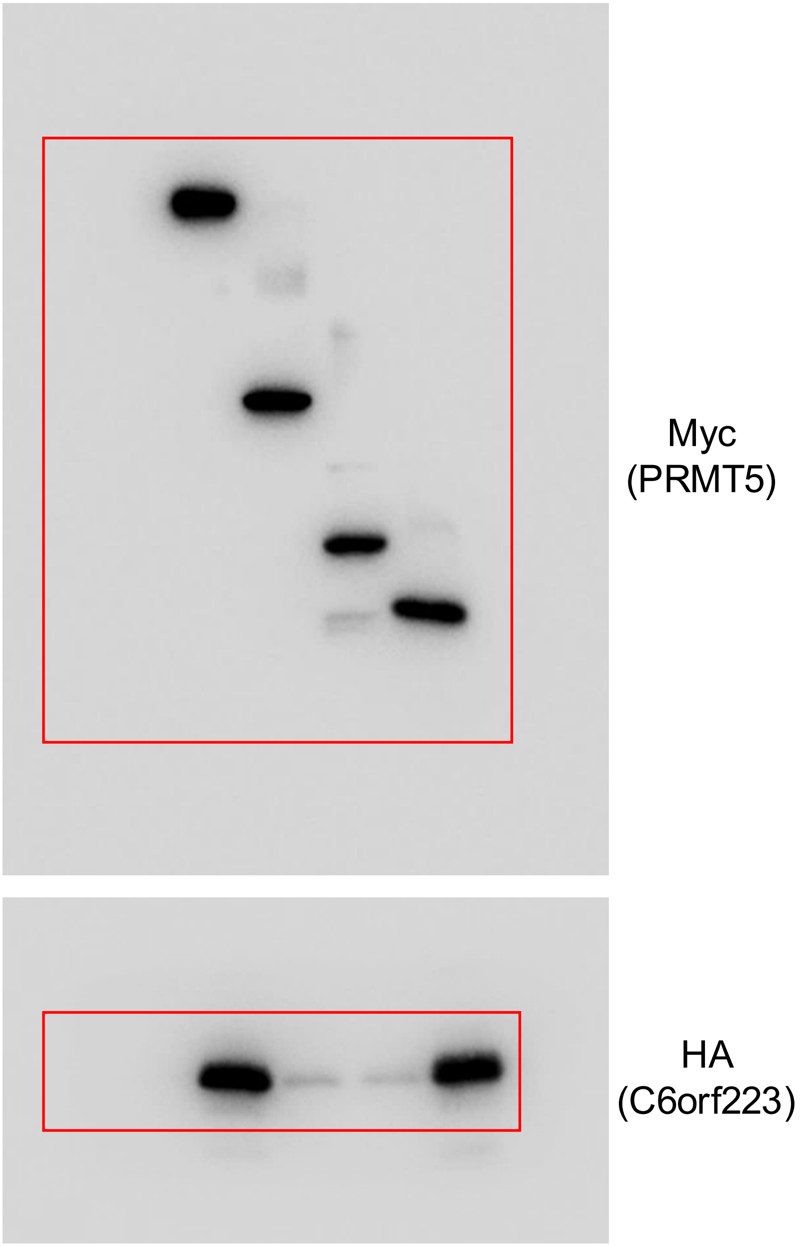

Full unedited blot/gel for Figure 3D

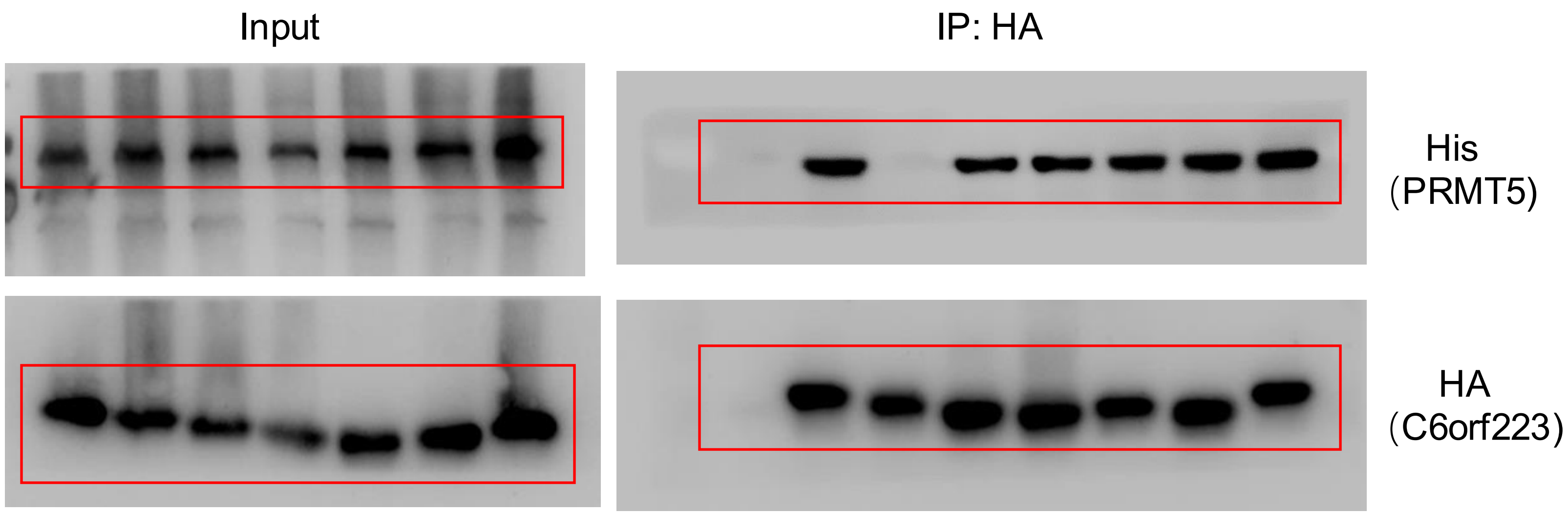

Full unedited blot/gel for Figure 3E

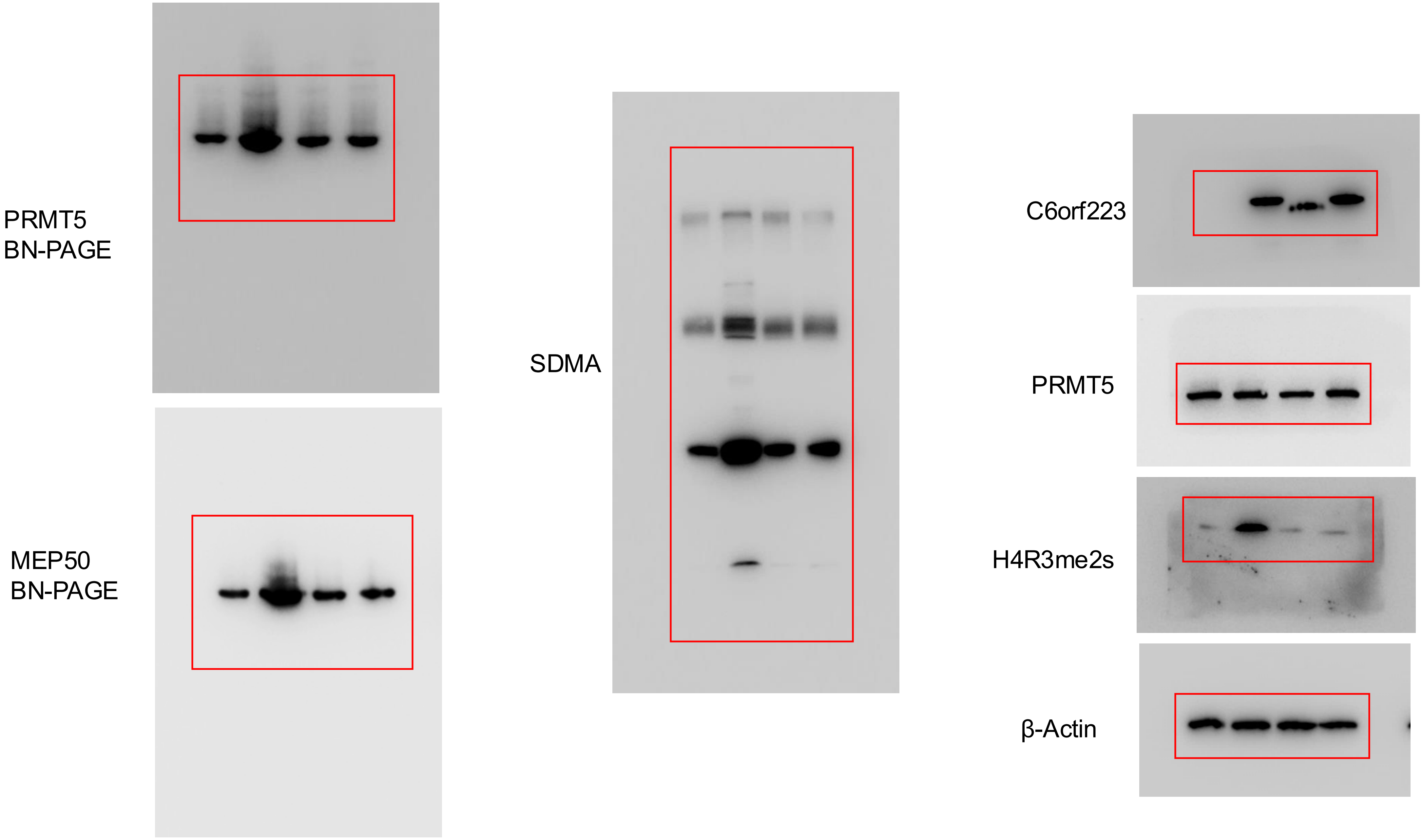

Full unedited blot/gel for Figure 3G

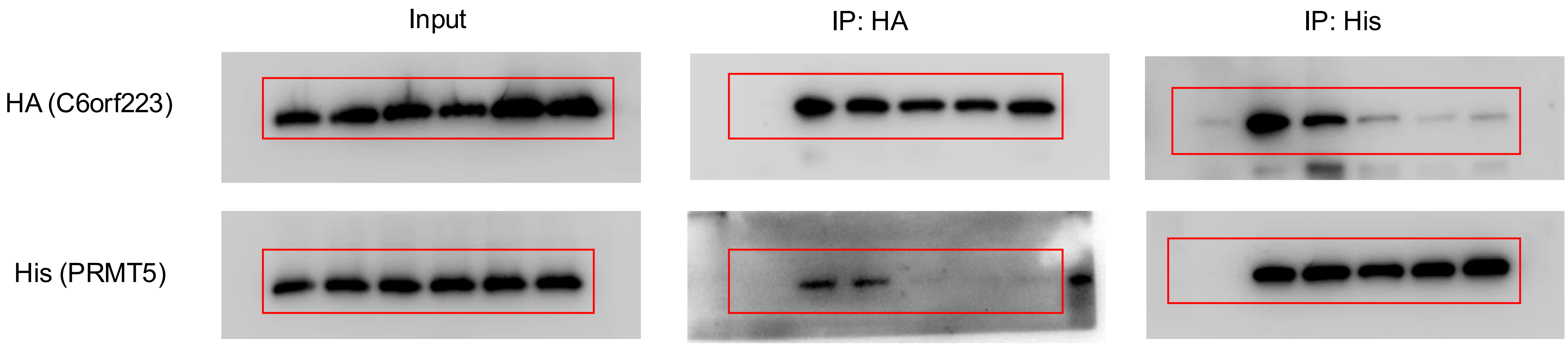

Full unedited blot/gel for Figure 4A

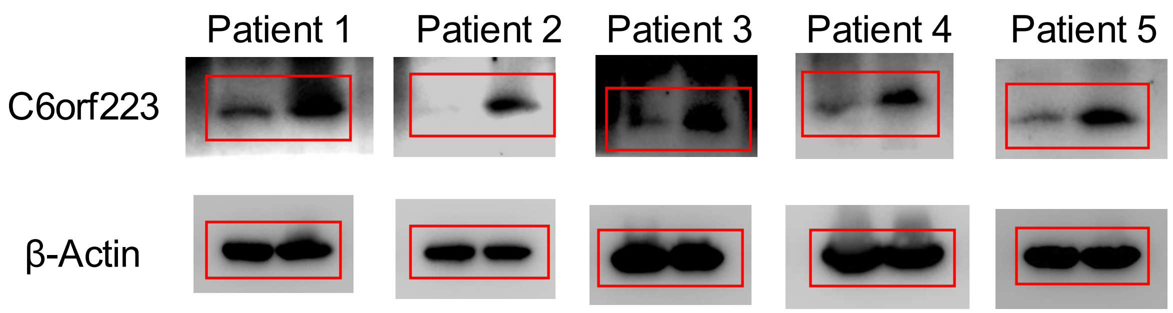

Full unedited blot/gel for Figure 4B

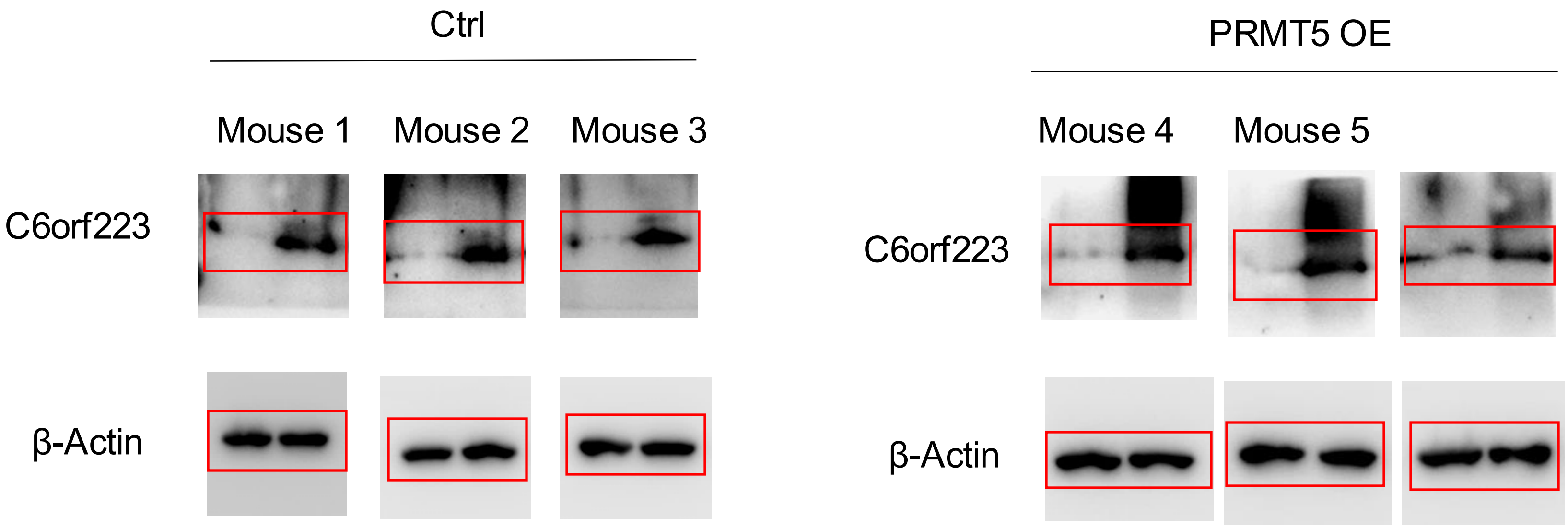

Full unedited blot/gel for Figure 6D

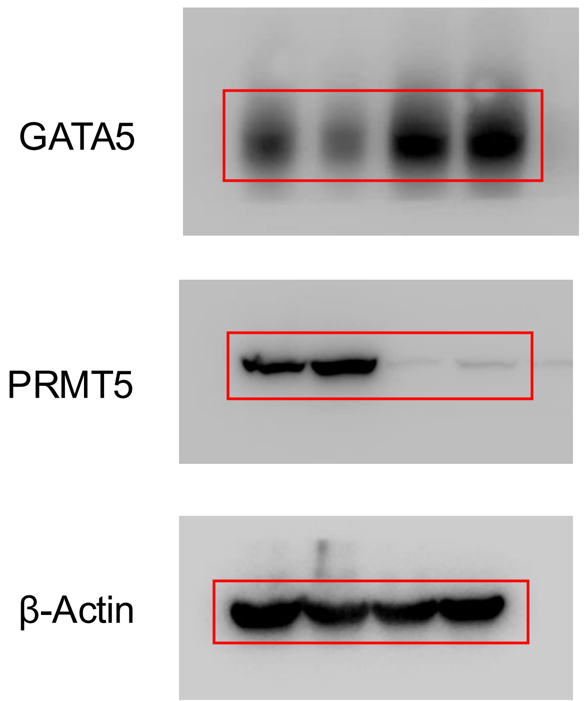

Full unedited blot/gel for Figure 6F

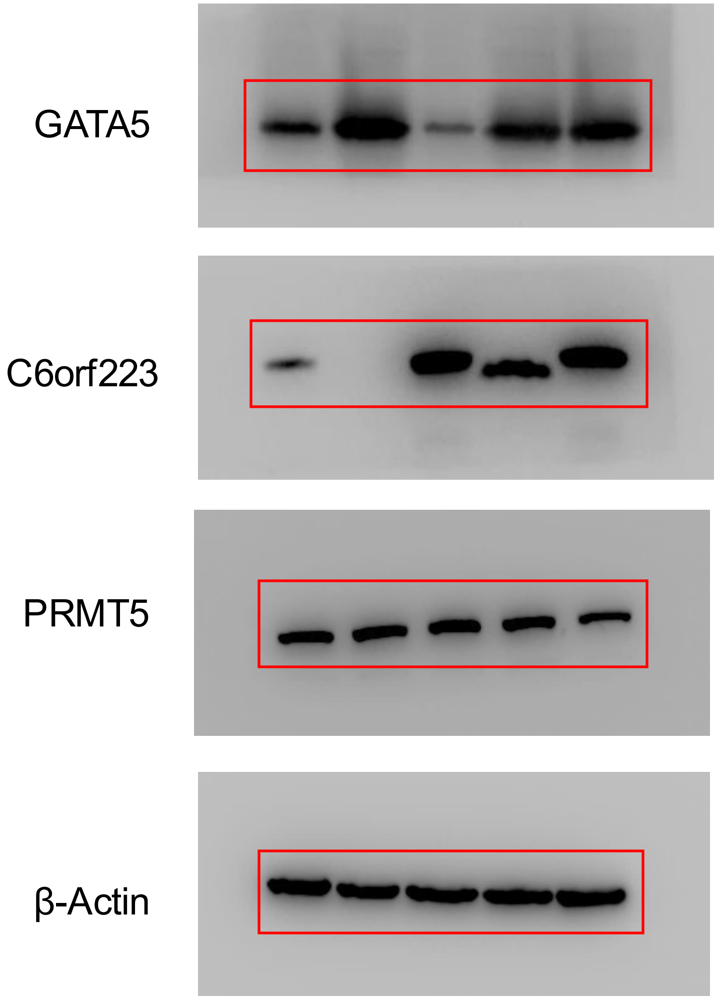

Full unedited blot/gel for Figure 8E

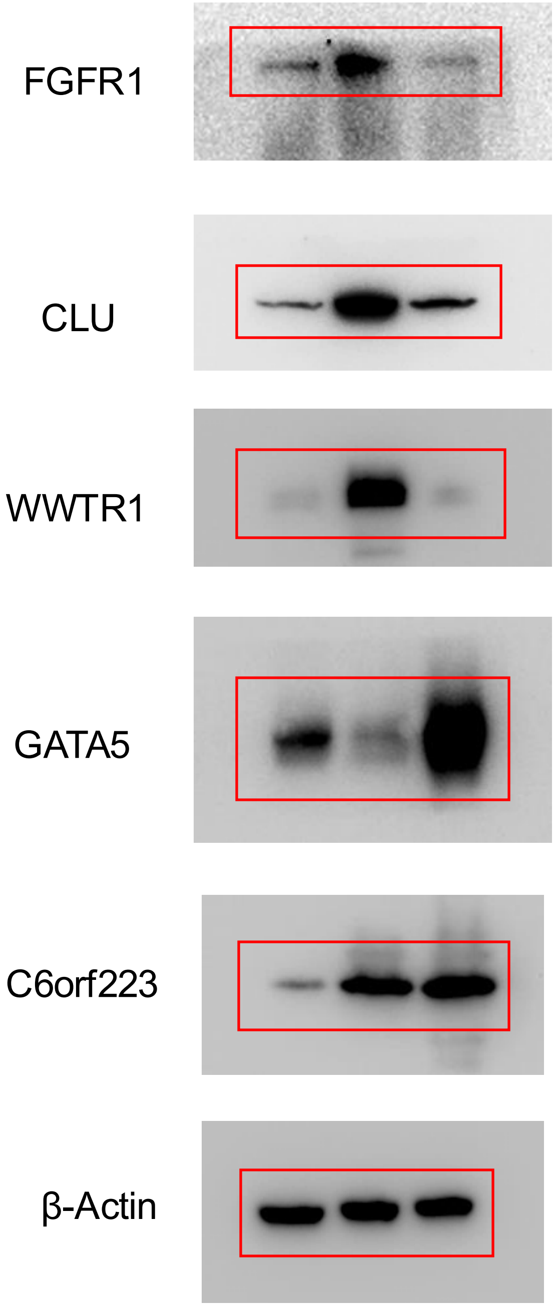

Full unedited blot/gel for Supplemental Figure 1A

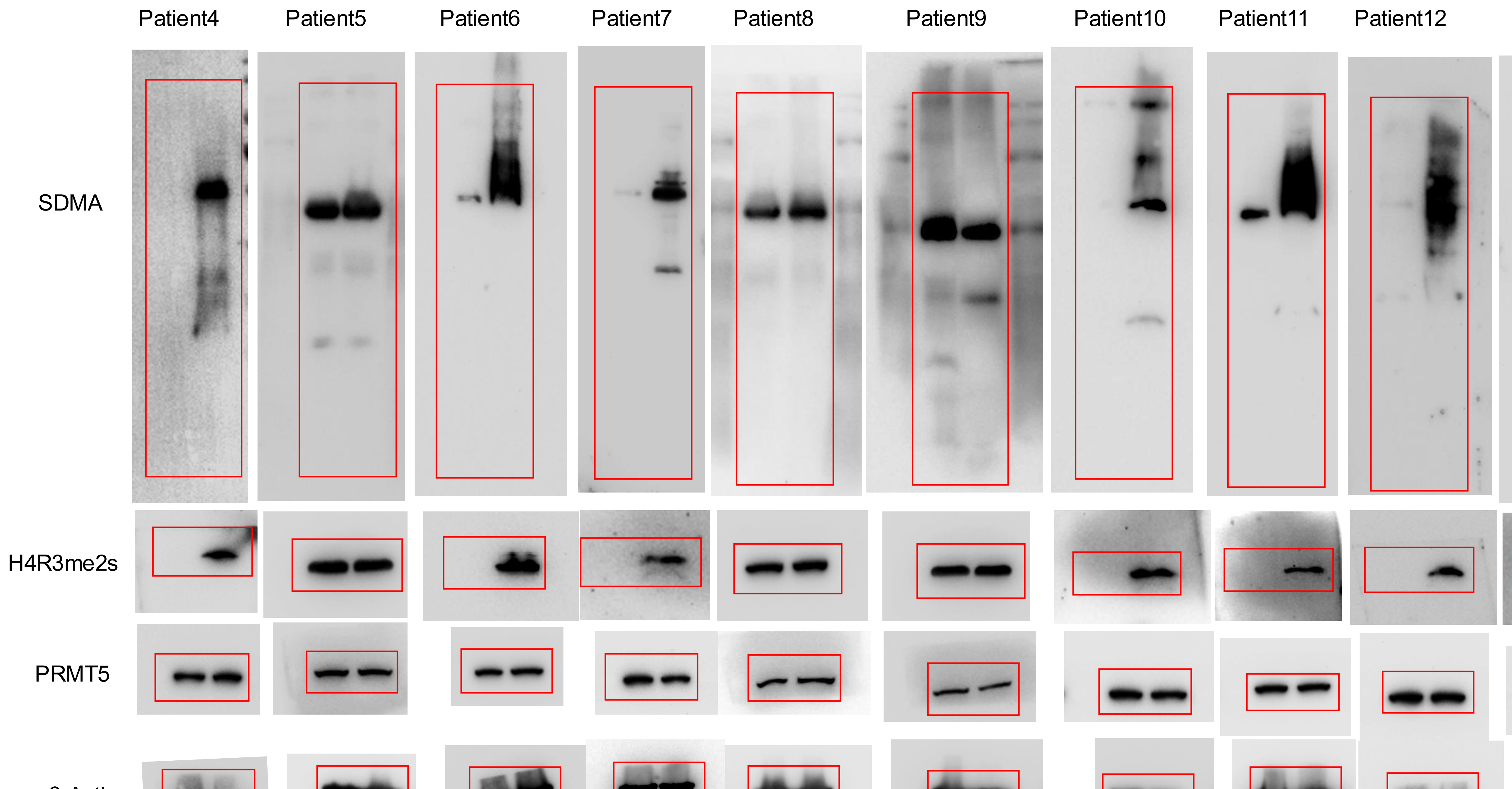

Full unedited blot/gel for Supplemental Figure 1C

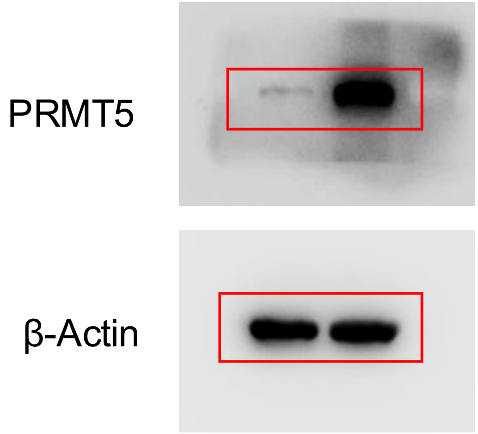

Full unedited blot/gel for Supplemental Figure 1J

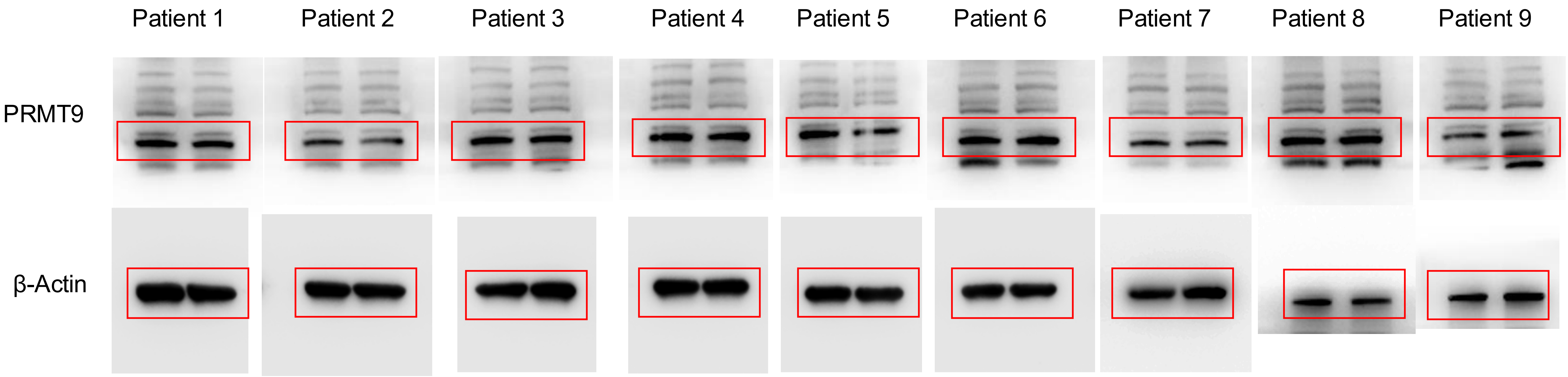

Full unedited blot/gel for Supplemental Figure 3A

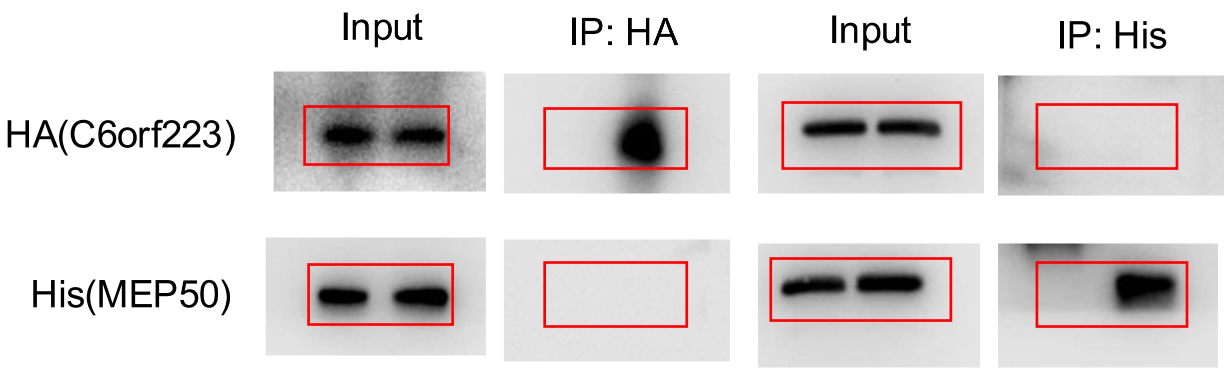

Full unedited blot/gel for Supplemental Figure 3B

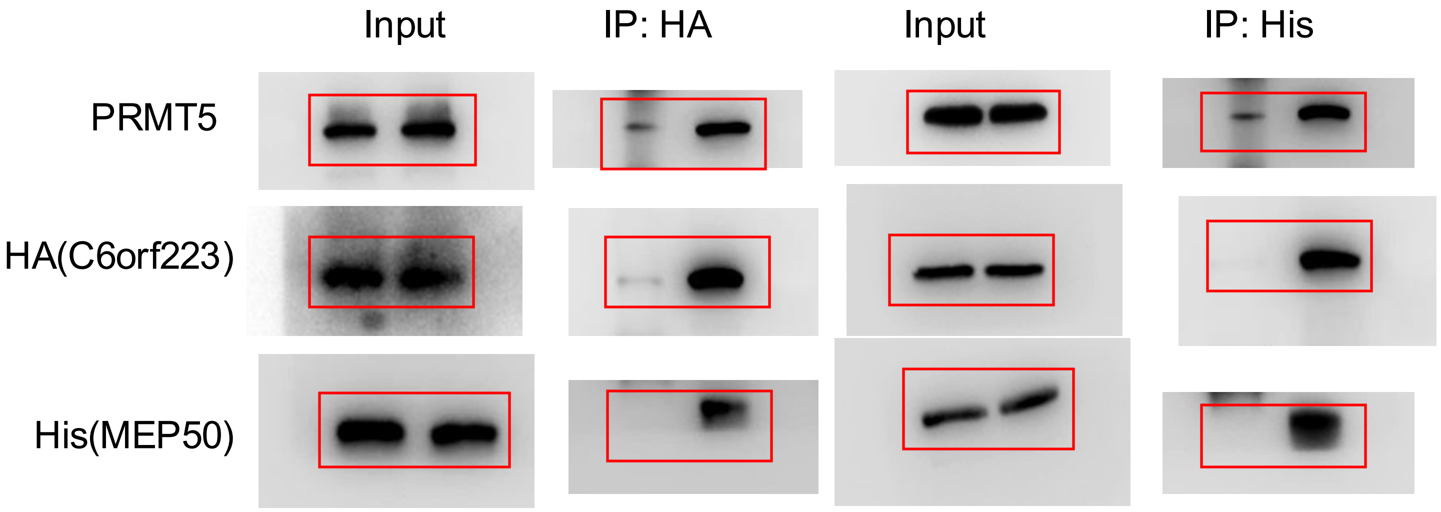

Full unedited blot/gel for Supplemental Figure 3E

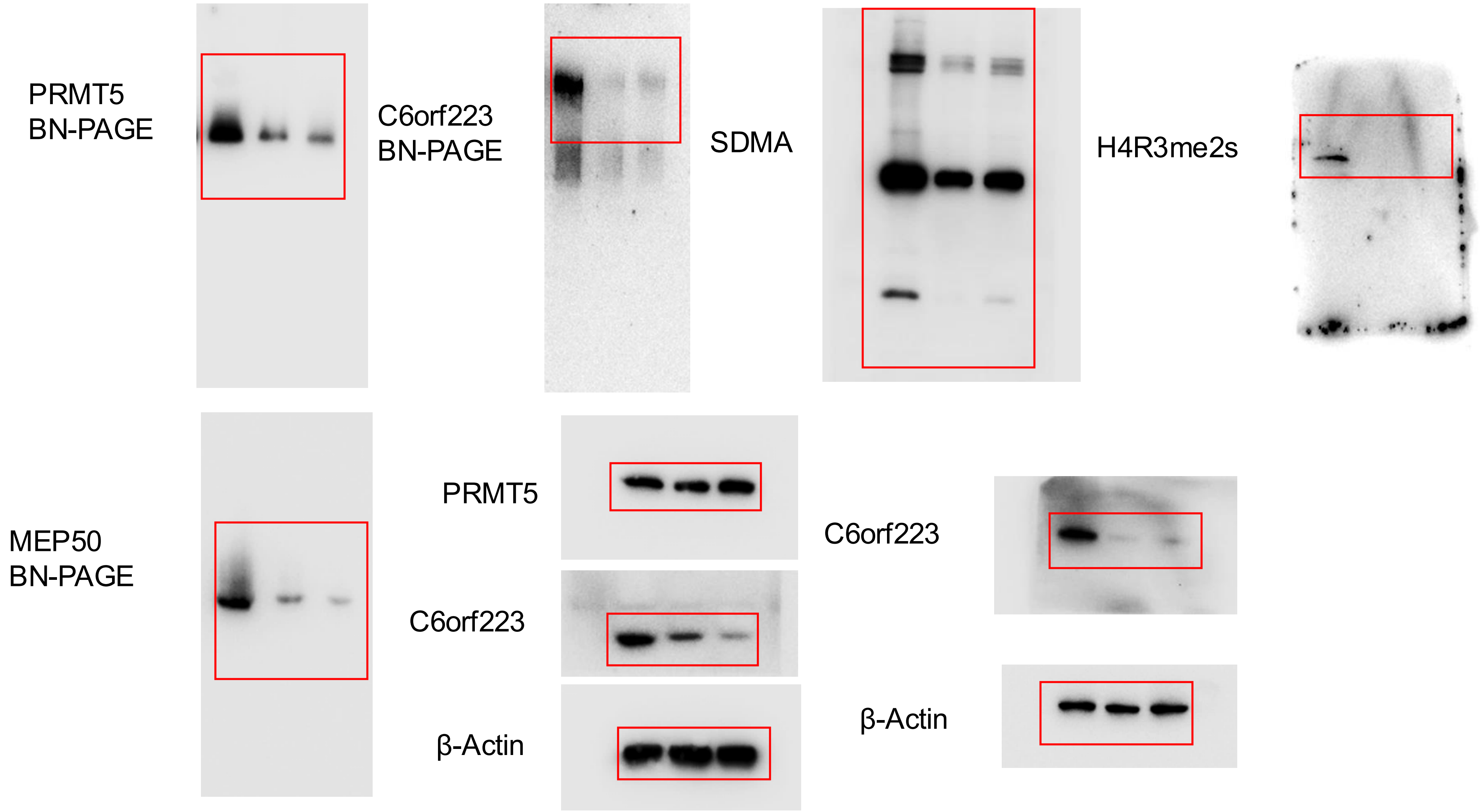

Full unedited blot/gel for Supplemental Figure 3G

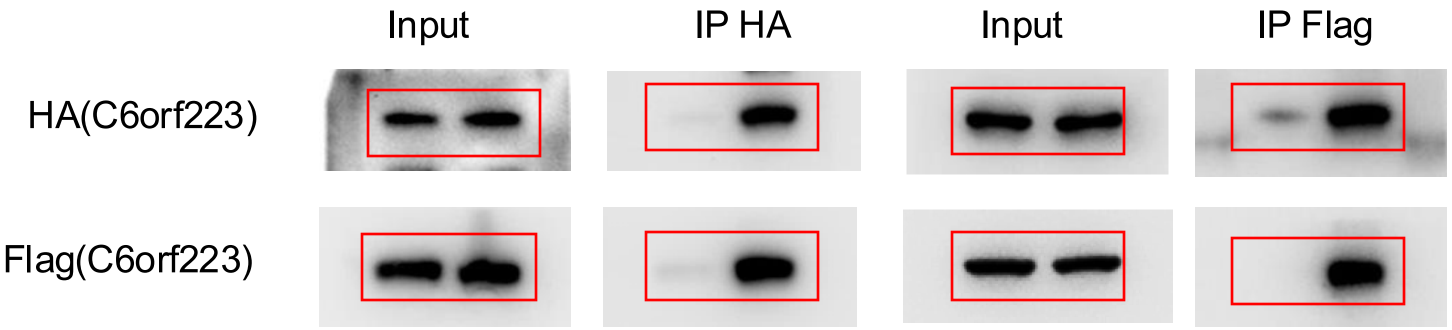

Full unedited blot/gel for Supplemental Figure 3H

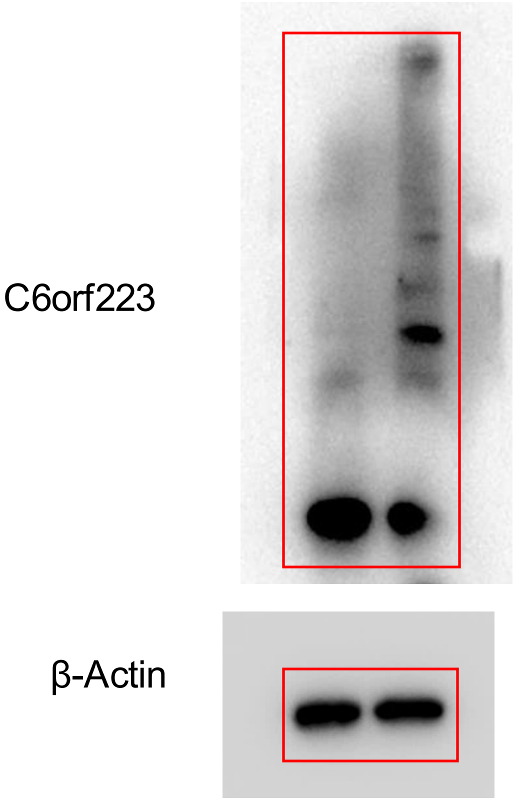

Full unedited blot/gel for Supplemental Figure 4B

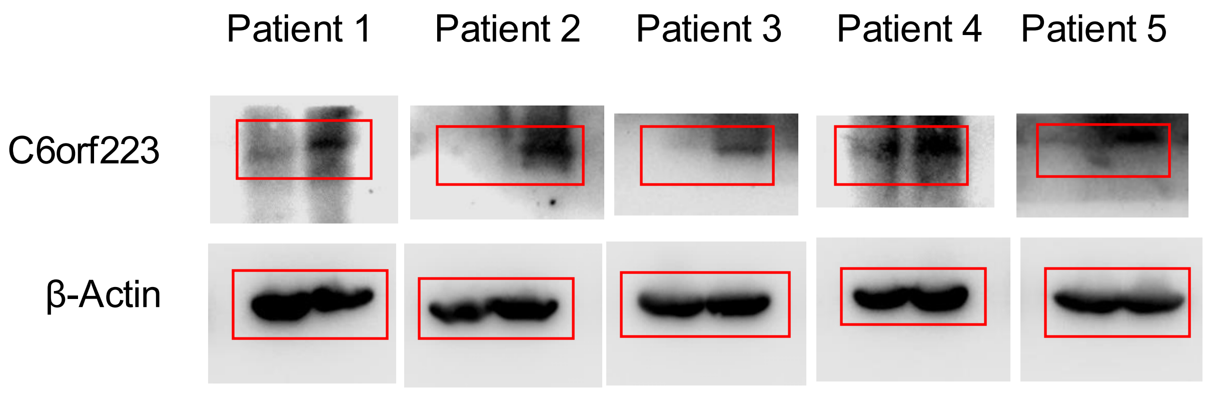

Full unedited blot/gel for Supplemental Figure 4C

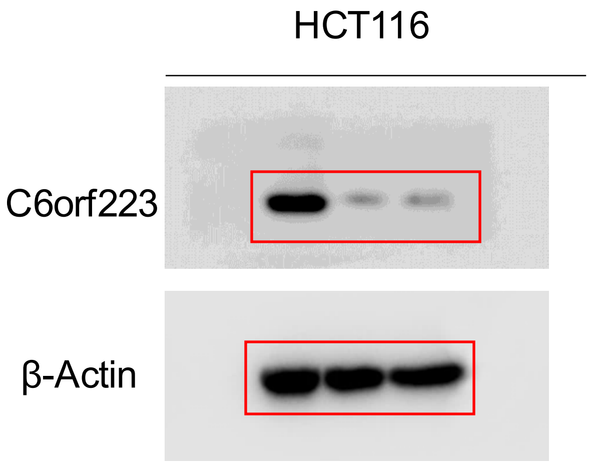

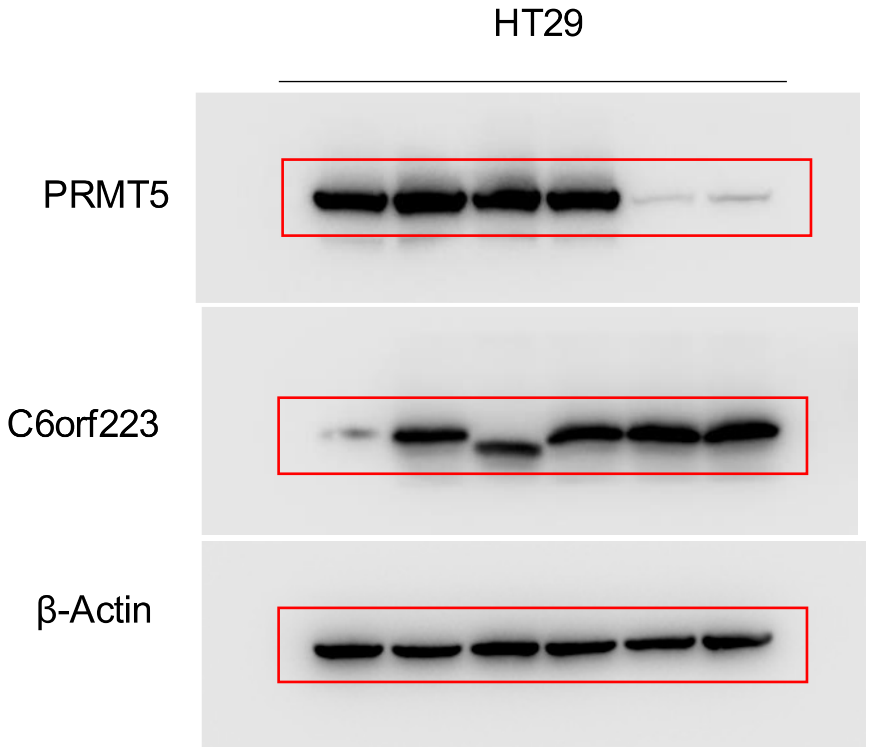

Full unedited blot/gel for Supplemental Figure 5A

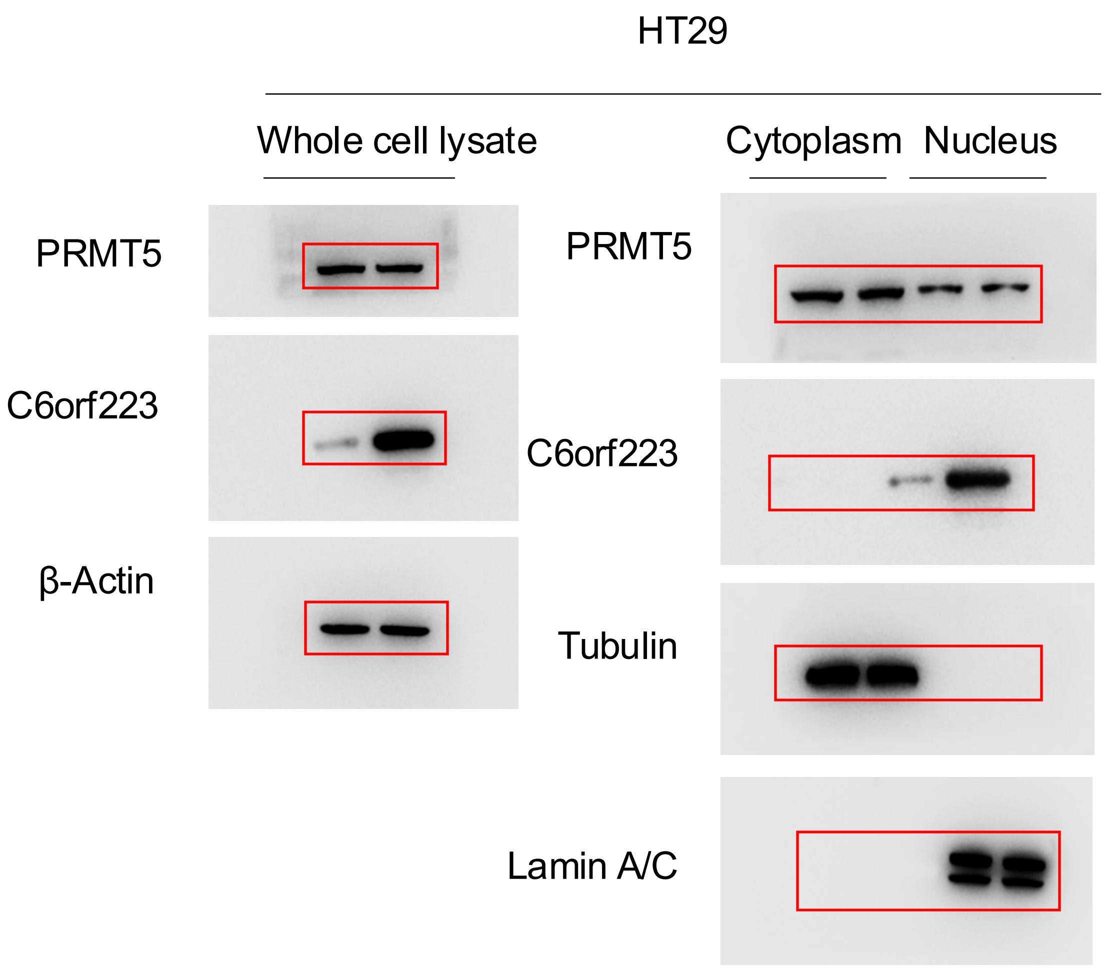

Full unedited blot/gel for Supplemental Figure 5B

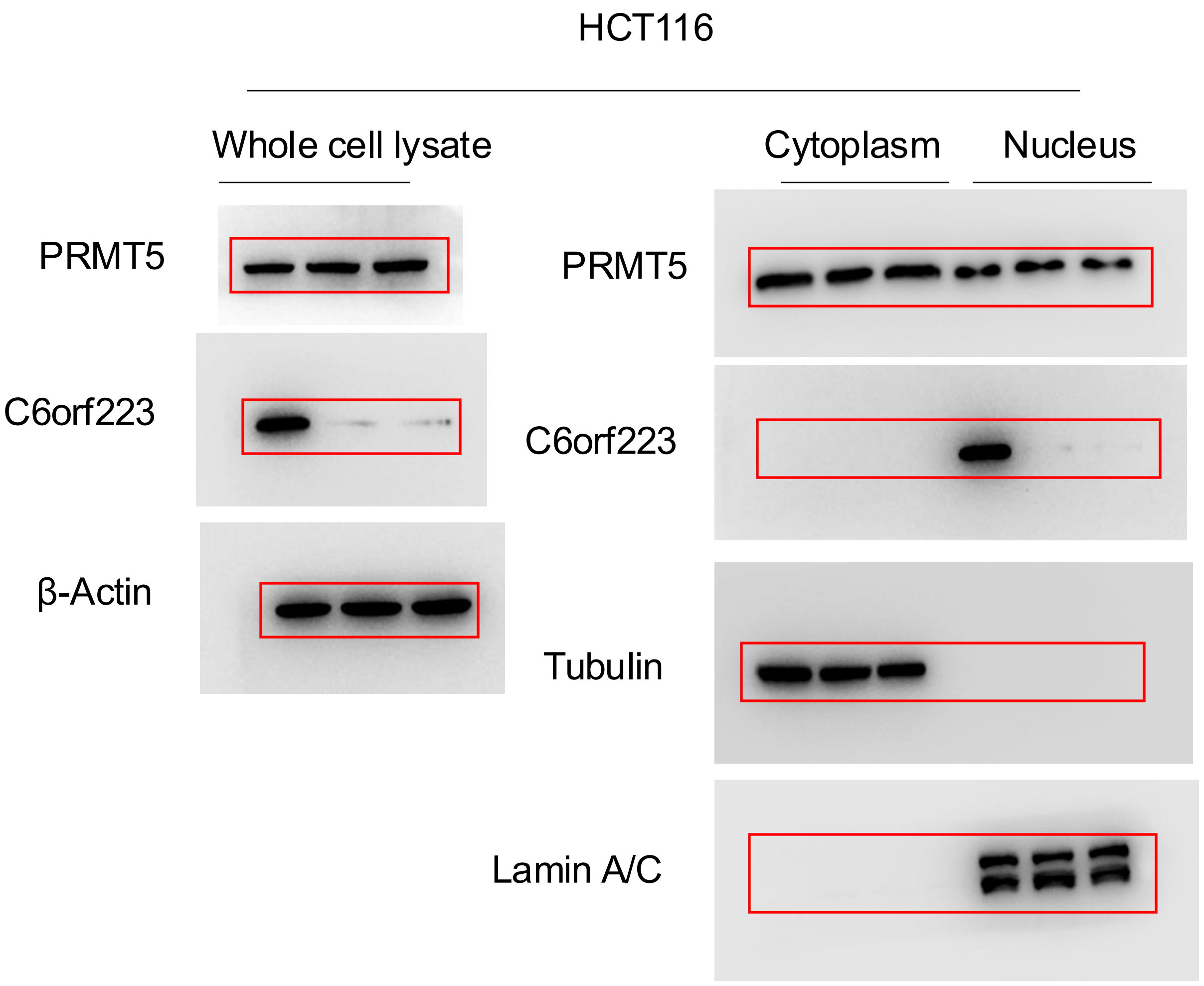

Full unedited blot/gel for Supplemental Figure 7F

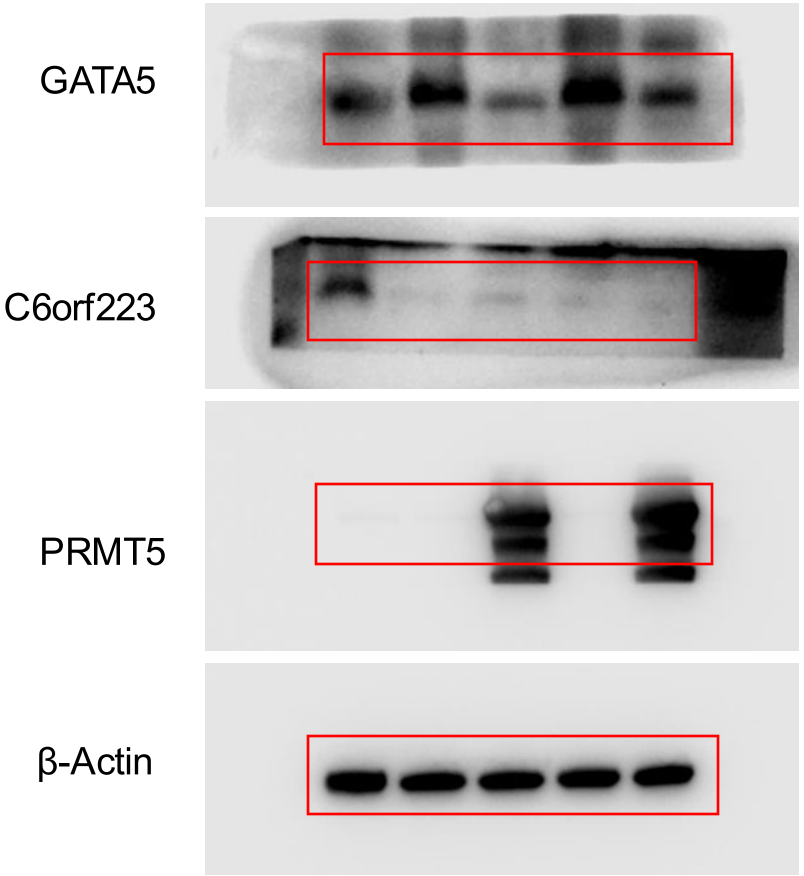

Full unedited blot/gel for Supplemental Figure 9A

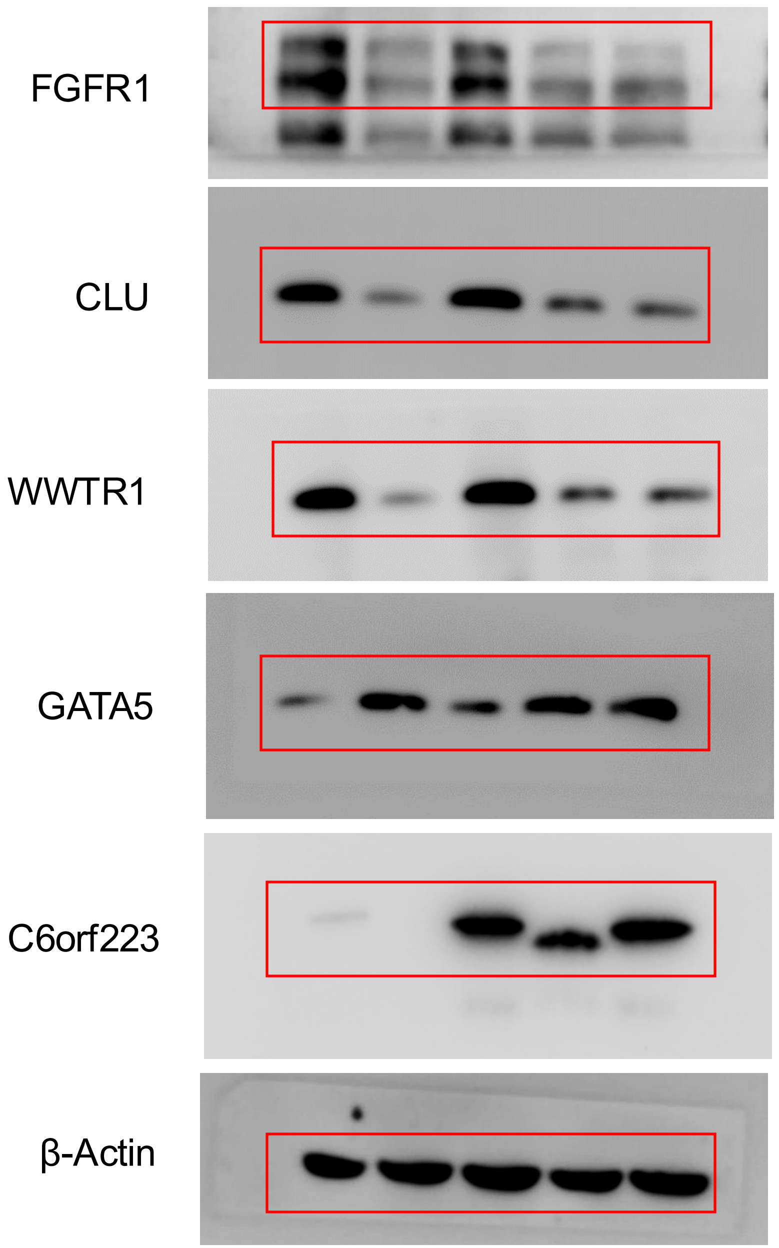

Full unedited blot/gel for Supplemental Figure 10D

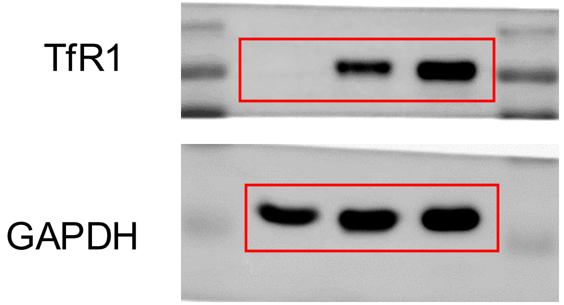

Supplement: Unedited blot and gel images [file jci-135-186052-s331.pdf]
